# Distinct transcription kinetics of pluripotent cell states

**DOI:** 10.15252/msb.202110407

**Published:** 2022-01-12

**Authors:** Rui Shao, Banushree Kumar, Katja Lidschreiber, Michael Lidschreiber, Patrick Cramer, Simon J Elsässer

**Affiliations:** ^1^ Science for Life Laboratory Department of Medical Biochemistry and Biophysics Division of Genome Biology Karolinska Institutet Stockholm Sweden; ^2^ Ming Wai Lau Centre for Reparative Medicine Stockholm node Karolinska Institutet Stockholm Sweden; ^3^ Department of Biosciences and Nutrition Karolinska Institutet Huddinge Sweden; ^4^ Department of Molecular Biology Max Planck Institute for Biophysical Chemistry Göttingen Germany

**Keywords:** mouse pluripotent stem cells, transcription termination, transcription unit annotation, transcription velocity, transient transcriptome sequencing, Chromatin, Transcription & Genomics, Stem Cells & Regenerative Medicine

## Abstract

Mouse embryonic stem cells (mESCs) can adopt naïve, ground, and paused pluripotent states that give rise to unique transcriptomes. Here, we use transient transcriptome sequencing (TT‐seq) to define both coding and non‐coding transcription units (TUs) in these three pluripotent states and combine TT‐seq with RNA polymerase II occupancy profiling to unravel the kinetics of RNA metabolism genome‐wide. Compared to the naïve state (serum), RNA synthesis and turnover rates are globally reduced in the ground state (2i) and the paused state (mTORi). The global reduction in RNA synthesis goes along with a genome‐wide decrease of polymerase elongation velocity, which is related to epigenomic features and alterations in the Pol II termination window. Our data suggest that transcription activity is the main determinant of steady state mRNA levels in the naïve state and that genome‐wide changes in transcription kinetics invoke ground and paused pluripotent states.

## Graphical abstract

Genome‐wide analyses of RNA metabolism kinetics in naïve, ground and paused pluripotent mouse embryonic stem cells (mESCs) indicate that the three states show markedly different global transcriptional kinetics.

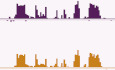

## Introduction

Pluripotency in the pre‐implantation embryo is of transient nature *in vivo*, but embryonic stem cells can be cultured long term in stable and interconvertible pluripotent states *in vitro*: in serum/leukemia inhibitory factor (LIF) media (SL, serum‐naïve state), serum‐free media containing LIF, Mek1/2 and GSK3*β* inhibitors (2i, naïve ground state), or serum/LIF media with mTOR inhibitor (mTORi, paused state). *In vitro* cultures of mESC provide facile model systems for understanding the molecular underpinnings of pluripotency and self‐renewal. The 2i‐induced ground state resembles naïve pre‐implantation E4.5 epiblast cells (Ying *et al*, 2008; Ghimire *et al*, 2018). Rewiring of signaling, metabolism, and epigenome have been observed in the SL–2i transition. CpG methylation is dramatically decreased genome‐wide, concomitant with a broad increase in H3K27me3 (Walter *et al*, 2016; Kumar & Elsässer, 2019). Further, a reduction of global H3K4me3 levels results in diminished promoter bivalency of developmentally regulated genes, where H3K27me3 and H3K4me3 are thought to set up a poised state (Marks *et al*, 2012; Sachs *et al*, 2013; Atlasi & Stunnenberg, 2017; Kumar & Elsässer, 2019). Enhancer activity is rewired during the SL–2i transition via Esrrb binding and H3K27ac activation (Atlasi *et al*, 2019). In contrast, mTOR inhibition suppresses cell growth and division while retaining pluripotency, resembling the diapaused blastocysts *in vivo* (Bulut‐Karslioglu *et al*, 2016).

Although most studies have focused on rewiring of regulatory circuits, activating, or disengaging individual enhancers and transcripts, globally reduced transcription activity in 2i and mTORi states was suggested by cell‐level 5‐Ethynyl Uridine (EU) incorporation (Bulut‐Karslioglu *et al*, 2016). Given that transcript levels are balanced by rates of RNA synthesis and degradation (Schwalb *et al*, 2016; Herzog *et al*, 2017), to which extent transcription activity itself determines transcript abundance in the pluripotent states remains to be addressed. Recent progress of quantitative RNA labeling techniques enable the absolute measurements of the rates of RNA synthesis and degradation (Rabani *et al*, 2011; Schwanhäusser *et al*, 2011; Schwalb *et al*, 2016; Herzog *et al*, 2017; Muhar *et al*, 2018; Schofield *et al*, 2018). TT‐seq combines a short 4‐thiouridine (4sU) RNA labeling pulse with an RNA fragmentation step to capture newly synthesized RNA, and thereby detects also short‐lived transcripts and allows estimation of the kinetics of RNA metabolism (Schwalb *et al*, 2016; Michel *et al*, 2017; Choi *et al*, 2021; Lidschreiber *et al*, 2021). To study transcriptional regulation in SL‐2i and SL–mTORi transitions, we measured total RNA and newly synthesized RNA with TT‐seq, annotated transcription units (TUs) *de novo*, and examined how transcription of different TU classes responds to state transitions. We also combined TT‐seq with quantitative RNA polymerase (Pol) II ChIP to estimate elongation velocities at the level of TUs. Our results led to a description of RNA metabolism in three states of pluripotent cells and to several unexpected findings.

## Results

### Transcription unit annotation in mESC pluripotent states

**Figure 1:**
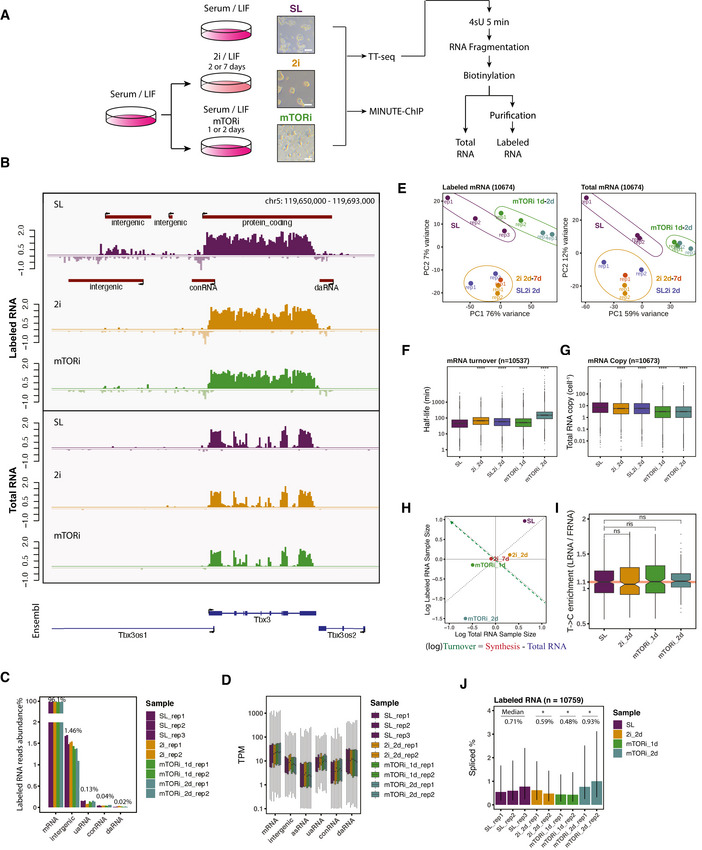
Mouse ES cells transcript annotation with TT‐seq AAn experimental scheme for transcription kinetics measurement in mESC samples under serum/LIF (SL), 2i/LIF (2i), serum/LIF/mTORi (mTORi) pluripotent states. Image scale bar is 100 μm.BTT‐seq‐labeled RNA and total RNA spike‐in normalized profiles (in log‐scale) on Tbx3 gene, with annotated TUs (red), and Ensembl gene references (blue) in mm10 genome.CLabeled RNA reads distribution by TU types in percentage (y axis breaks from 2 to 40%).DLabeled RNA TPM (transcript per million) by TU types of each sample and replicate. Boxplots are with central bands at the median, 0.25 and 0.75 quartiles box area, 1.5× interquartile range (IQR) whiskers; outliers are hidden.EPrincipal component analysis of labeled RNA (left) and total RNA (right) log RPK by sample replicates. SL‐naïve, 2i‐ground, and mTORi‐paused states are indicated. Serum LIF medium with 2i supplement was used to confirm the majority of 2i‐induced expression changes (Fig EV2G).F, GSpike‐ins scale estimated mRNA half‐life and copy number per cell (Two‐tailed unpaired Student's *t*‐test ****P < 0.0001, in comparison with SL state). Boxplots are with central bands at the median, 0.25 and 0.75 quartiles box area, 1.5× interquartile range (IQR) whiskers; outliers are hidden.HLabeled and total RNA sample sizes distribution of the mESC pluripotent states. Relative sample sizes are represented by the log‐transformed DESeq's size factors after spike‐in normalization and replicates combined (left). Cell‐level RNA turnover approximation by the contrast of labeled and total size factors, which equals the projection on the green diagonal dashed line.IFrequency enrichment ratio of T‐>C conversions in labeled (LRNA) versus total fragmented RNA (FRNA) reads in the different cell culture conditions. Two‐tailed unpaired Student's *t*‐test was performed, in comparison with SL state. Boxplots are with central bands at the median, 0.25 and 0.75 quartiles box area, 1.5× interquartile range (IQR) whiskers; outliers are hidden.JDistribution of spliced labeled RNA reads percentages (CIGAR ‘N’) on Ensembl protein‐coding genes. The error bar shows the (0.25, 0.75) quantile of spliced ratio, and the bar height represents the median spliced rate. Two‐tailed unpaired Student's *t*‐test *P < 0.05, in comparison with SL state.

**Figure EV1:**
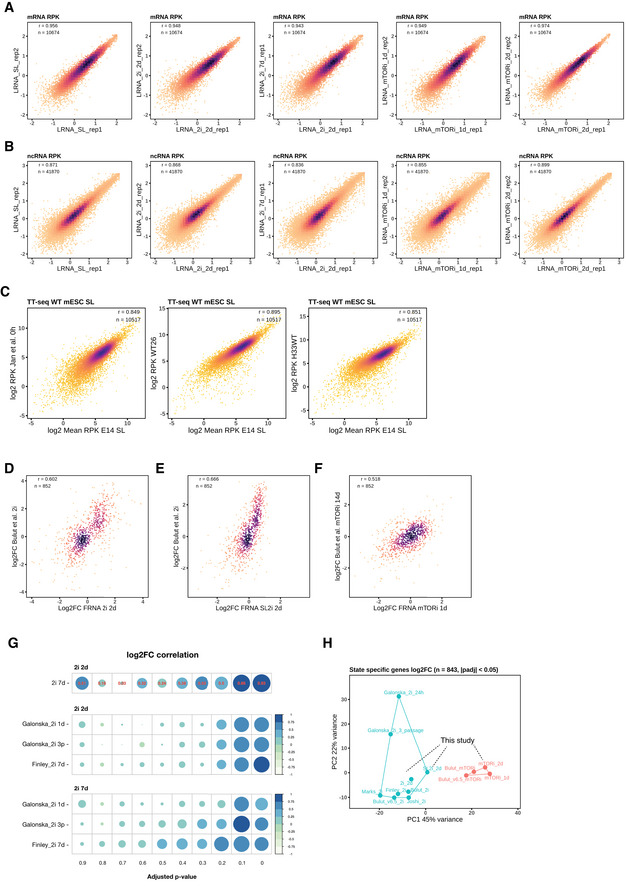
Reproducibility evaluation of TT‐seq in the pluripotent states ATT‐seq mRNA log RPK correlation between replicates. Spearman's rank correlation coefficient was performed, the same as below.BTT‐seq annotated ncRNA log RPK correlation between replicates.CTT‐seq‐labeled RNA gene RPK correlation among wild‐type mESCs in SL state. RW4 E14 is the strain used in this study (male, 129X1/SvJ), compares with TX1072 (female, C57BL/6) (Żylicz *et al*, 2019), WT26 (E14, male, C57Bl/6J) (Elsässer *et al*, 2015), and H33WT (E14, male, 129×C57Bl/6J) (Elsässer *et al*, 2015). Spearman's correlation coefficient is shown.D–FTT‐seq fragmented total RNA (FRNA) log_2_FC of 2i 2d, SL2i 2d, and mTORi 1d correlations with published 2i and mTORi changes (Bulut‐Karslioglu *et al*, 2016). Significantly changed genes (*P*.adj < 0.05) in any of our state transitions are compared (*n* = 852), the same for H.GComparison of DESeq2's log_2_FC correlation between total RNA 2i 2 days changes with public RNA‐seq data (Galonska *et al*, 2015; Finley *et al*, 2018). Both our data and the public data were counted with Kallisto and tested with DESeq2, and Pearson's correlation was performed at each degree of average *P*.adjust values in the five samples.HPCA plot with RNA‐seq log_2_FC of differentially expressed genes (state specific genes), called from our ground and paused state transitions, including published studies (Marks *et al*, 2012; Galonska *et al*, 2015; Joshi *et al*, 2015; Bulut‐Karslioglu *et al*, 2016; Finley *et al*, 2018).

**Figure EV2:**
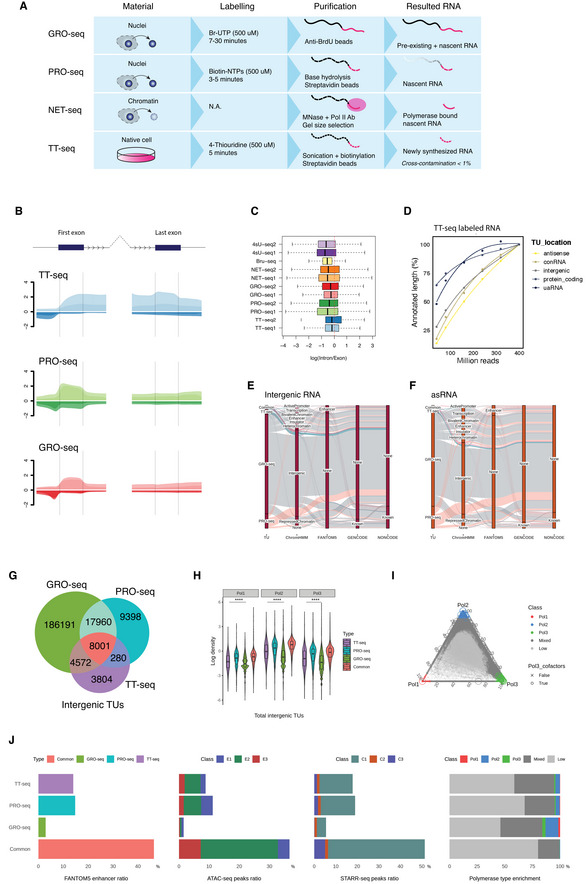
Nascent RNA‐seq methods comparison AA brief scheme of GRO‐seq, PRO‐seq, NET‐seq, and TT‐seq workflows of RNA labeling and extraction.BRead coverages on the first and the last exons with 2‐kb flanking regions and the adjacent introns (arrows). Exon and intron coverages are resized to the same dimension and plotted by log mean.CThe ratios of intron versus exon with labeled RNA reads density on Ensembl protein‐coding genes across the nascent RNA‐seq methods. Boxplots are with central bands at the median, 0.25 and 0.75 quartiles box area, 1.5× interquartile range (IQR) whiskers; outliers are hidden. (TT‐seq1, this study; TT‐seq2, Żylicz *et al*, 2019); (GRO‐seq1, Flynn *et al*, 2016; GRO‐seq2, Wang *et al*, 2015); (PRO‐seq1, Engreitz *et al*, 2016; PRO‐seq2, Lloret‐Llinares *et al*, 2018); (NET‐seq1, Mylonas & Tessarz, 2018; NET‐seq2, Tuck *et al*, 2018); (4sU‐seq1, Benabdallah *et al*, 2019; 4sU‐seq2, Brown *et al*, 2017); (Bru‐seq, Ardehali *et al*, 2017).DTU annotation relative total length recovery test by TT‐seq bam file random subsetting under (0.1, 0.2, 0.4, 0.6, 0.8, 1) for different TU types.E, FSankey plots compare the intergenic and the cis‐antisense RNAs annotated by GRO‐seq, PRO‐seq, and TT‐seq. Fractions of annotated TUs matching with public references (GENCODE (Frankish *et al*, 2021), FANTOM5 enhancer (Andersson *et al*, 2014), NONCODE (Zhao *et al*, 2016)) are indicated.GVenn diagram of GRO‐seq, PRO‐seq, and TT‐seq intergenic RNAs.HRNA Pol I, II, and III occupancy (Jiang *et al*, 2020) on the total intergenic TUs. Boxplots are with central bands at the median, 0.25 and 0.75 quartiles box area, 1.5× interquartile range (IQR) whiskers; outliers are hidden. Two‐tailed unpaired Student's *t*‐test is performed with the common TUs against the method‐specific TUs (****P < 0.0001).IA ternary plot of Pol I, II, and III enrichment on the GRO‐seq, PRO‐seq, and TT‐seq combined intergenic TU annotations. Pol III cofactors binding sites (Carrière *et al*, 2012) are pinpointed for Pol III class assignment cross‐validation.JCommon overlapped and method‐specific intergenic RNAs proportions with FANTOM5 enhancers (Andersson *et al*, 2014), ATAC‐seq peaks (Atlasi *et al*, 2019), STARR‐seq peaks (Peng *et al*, 2020), and Pol I‐III occupancy in H.

To capture transcriptional responses to different pluripotent state transitions, we switched ES cells from SL medium to 2i medium for 2 or 7 days, mTORi medium for 1 or 2 days, and under each condition pulsed 4‐thiouridine (4sU) for 5 min and performed the TT‐seq method as previously described (Schwalb *et al*, 2016) (Fig 1A). TT‐seq data were highly reproducible between biological replicates (Fig EV1A–C), and recapitulated the reported expression changes in the early state transitions (Fig EV1D–H); TT‐seq data of cells cultured in 2i medium for 2 or 7 days correlated well (Fig EV1A) and we therefore focused on the primary responses after 2 days in all further analyses. In order to verify our labeled RNA results, we compared TT‐seq gene coverage with GRO‐seq (global run‐on sequencing) (Wang *et al*, 2015; Flynn *et al*, 2016), PRO‐seq (precision run‐on sequencing) (Engreitz *et al*, 2016; Lloret‐Llinares *et al*, 2018), NET‐seq (native elongating transcript sequencing) (Mylonas & Tessarz, 2018; Tuck *et al*, 2018), 4sU‐seq (Brown *et al*, 2017; Benabdallah *et al*, 2019), and Bru‐seq (bromouridine sequencing) (Ardehali *et al*, 2017). TT‐seq 4sU labeling was performed within a short time window (5 min) under native cell culture condition, and total RNA was fragmented before biotinylation and purification steps (Fig EV2A). Therefore, TT‐seq‐labeled RNA showed a steady coverage over the gene body, more balanced signal between the first and the last exons, as well as the highest intron/exon coverage ratio as compared to NET‐seq, PRO‐seq, and GRO‐seq (Figs 1B and, EV2B and C). The stranded TT‐seq signal, thus, provides an excellent demarcation of the transcription unit and polymerase‐independent measure of transcriptional activity.

**Figure EV3:**
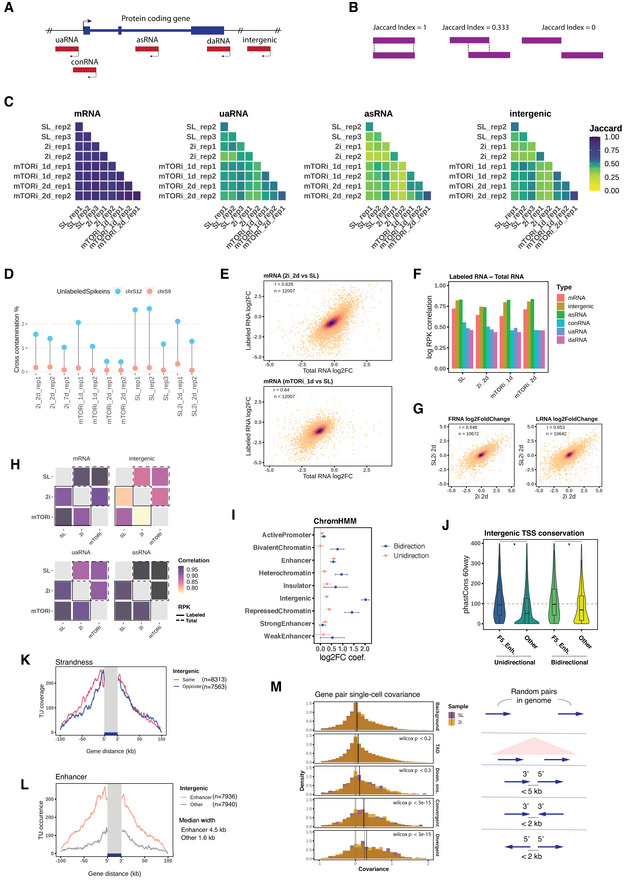
TU annotation and transcription variation of mESC pluripotent states AGene‐associated ncRNA types are classified by their TSS locations relative to the gene TSS: upstream antisense RNA (−1 kb, 0), convergent RNA (0, +1 kb), cis‐antisense RNA (within gene body), downstream antisense RNA (TES, TES + 1 kb), and intergenic RNA.BIllustration of Jaccard index for annotated TUs comparison.CJaccard indexes of mRNA, uaRNA, asRNA, and intergenic RNA intervals similarity in pairwise comparison between each sample replicate.DLabeled RNA purification cross‐contamination rates calculated as the percentage of unlabeled spike‐in reads compared to all spike‐in reads in respective labeled libraries.EPearson's correlations between mRNAs' total and labeled log_2_FC in 2i 2 days and mTORi 1‐day transition.FPearson's correlations between labeled RNA and total RNA log RPK by each TU location and pluripotent state with merged replicates and log RPK on the combined TUs, the same for H.GCorrelation between 2i and SL2i ground states changes by total (FRNA) and labeled (LRNA) mRNA log_2_FCs.HPearson's correlations between the pluripotent states by labeled and total RNA log RPK of averaged replicates.IThe coefficients of ChromHMM states are in response to the internal normalized intergenic RNA log_2_FC differential transcription in 2i 2‐day transition. Two separate logistic regression models are trained for the unidirectional and bidirectional intergenic TUs to predict their log_2_FC with ChromHMM states. Each states' coefficients are plotted with the respective logistic regression confidence intervals.JIntergenic TU promoter (−500, 200 bp) evolution conservation scores from phastCons 60way (Siepel *et al*, 2005) with the same groups as in Fig 2C (two‐tailed unpaired Student's *t*‐test *P < 2.2e‐16). Boxplots are with central bands at the median, 0.25 and 0.75 quartiles box area, 1.5× interquartile range (IQR) whiskers; outliers are hidden.KIntergenic TU intervals stacked total coverage in the ± 100 kb gene neighborhoods by relative strandedness.LIntergenic TU intervals stacked coverage by enhancer and other states in the ± 100 kb gene neighborhoods (*n* = 11,684).MSingle‐cell gene expression (Buettner *et al*, 2015) covariance distribution by the gene pairs of random background (*n* = 4,000), within a topological associated domain (TAD; *n* = 3,250), consecutive downstream sense (*n* = 2,058), 3′ ends convergence (*n* = 3,206), and promoter divergence (*n* = 3,036). Wilcoxon test is performed against the background covariance. Median covariances of each gene positioning type are indicated for both SL and 2i states.

The pluripotent ES cell genome is pervasively transcribed (Efroni *et al*, 2008). We used labeled TT‐seq signals to *de novo* define coding and non‐coding TUs in the pluripotent genome separately for each condition and replicate. An R shiny application (TU filter) was developed offering previously described algorithms for TU discovery (Schwalb *et al*, 2016) within a simple and reproducible visual workflow (Materials and Methods). Overall, 96% of the uniquely mapped TT‐seq‐labeled RNA reads could be assigned to 11,743 GENCODE protein‐coding genes, while approximately 1.5% were assigned to 20,437 new intergenic RNAs called from TT‐seq (Fig 1C). We automatically annotated TUs by their location and direction relative to transcription start site (TSS) or transcription termination site (TTS) of protein‐coding TUs: upstream antisense (uaRNA), convergent (conRNA), *cis*‐antisense (asRNA), downstream sense (dsRNA), and downstream antisense (daRNA) TUs (Fig EV3A). An example of the TU annotation is shown for the Tbx3 gene neighborhood (Fig 1B). The sensitivity of our TU discovery pipeline was majorly determined by sequencing depth (Fig EV2D), since accuracy of TU calling depends on continuous read coverage. Transcription of intergenic TUs was generally lower than that of protein‐coding genes, suggesting that their cryptic/non‐canonical promoters are generally weaker than divergent gene promoters (Fig 1C). Moreover, asRNA TUs were generally most lowly transcribed (Fig 1D) and exhibited higher interval inconsistency between replicates (Fig EV3B and C). uaRNAs, commonly arising from divergent transcription initiation at gene promoters, were most robustly called and relatively abundant amongst the non‐coding transcripts (Figs 1C–D, EV2D and EV3C).

We also applied the same annotation process to published GRO‐seq and PRO‐seq data to test if lowly expressed TUs were reproducible. GRO‐seq and PRO‐seq identified a larger number of non‐annotated and method‐specific short ncRNAs compared to TT‐seq, reflecting the different labeling preferences of metabolic and run‐on labeling approaches (Fig EV2E–G). 8,001 intergenic TUs commonly called with all three methods showed the highest proportion of FANTOM5 enhancers (Andersson *et al*, 2014), ATAC‐seq peaks (Atlasi *et al*, 2019), STARR‐seq enhancers (Peng *et al*, 2020), but did not show discernable ChIP‐seq signal enrichment for any of the three RNA polymerases (Fig EV2H–J). Hence, the integration of nascent RNA mapping techniques would allow the most robust selection of transcribed TUs with regulatory potential.

### Decreased RNA synthesis and transcript copy in 2i and mTORi conditions

Next, we assessed RNA synthesis and turnover changes associated with ground state and paused state transitions using TT‐seq total and labeled RNA from the biological replicates (Fig 1E). We used a set of labeled and unlabeled spike‐in RNAs to scale labeled and total RNA, and predicted RNA turnover and copy number (Materials and Methods). 2i and mTORi treatment slowed down RNA turnover and decreased total RNA abundance per cell (Bulut‐Karslioglu *et al*, 2016) (Fig 1F and G). Alternatively, contrasting labeled and total transcriptome size factors also demonstrated that 2i and mTORi states had a smaller labeled‐to‐total RNA ratio, hence, slower cell‐level turnover (Fig 1H). Dilution of RNA by cell division and RNA degradation jointly determine cell‐level RNA turnover, and the observed reduction of turnover in 2i and mTORi is in line with a slowdown of cell cycle progression in the respective states (Bulut‐Karslioglu *et al*, 2016). To exclude the possibility that the different states would have intrinsically different labeling efficiencies, we first confirmed the purity of labeled RNA by assessing cross‐contamination using the spike‐in RNAs (Fig EV3D). Further, we exploited the known tendency of 4sU to mispair with C (leading to a ~10% conversion rate in reverse transcription reactions) (Herzog *et al*, 2017), to compare the frequency of 4sU incorporation across the pluripotent states. Stable conversion rates suggested a similar incorporation efficiency in all states (Fig 1I). Moreover, analysis of spliced read fraction across the different states showed only minor differences (Fig 1J). In summary, these quality controls assured us that newly synthesized transcripts were labeled consistently and isolated with high purity, therefore, measuring the unbiased RNA synthesis in the pluripotent states.

### Genome neighborhood modulates local transcription variation

Next, we evaluated the manner of transcription variation in the state transitions. To assess the contribution of RNA synthesis to changes in total RNA abundance, we compared the pairwise correlation of the new states' labeled RNA and total RNA log_2_FC (log_2_ fold change), which suggests that mRNA abundance change is dominantly regulated via RNA synthesis. Further with length‐normalized read counts (log‐RPK), mRNA, intergenic RNA, and asRNA‐labeled RNA levels correlated well with their total RNA levels (Fig EV3E and F), indicating a lesser impact of degradation. On the other hand, ncRNAs, in particular uaRNAs, showed little correlation between labeled and total RNA changes, suggesting that their steady‐state levels are specifically regulated by degradation (Fig EV3F).

**Figure 2:**
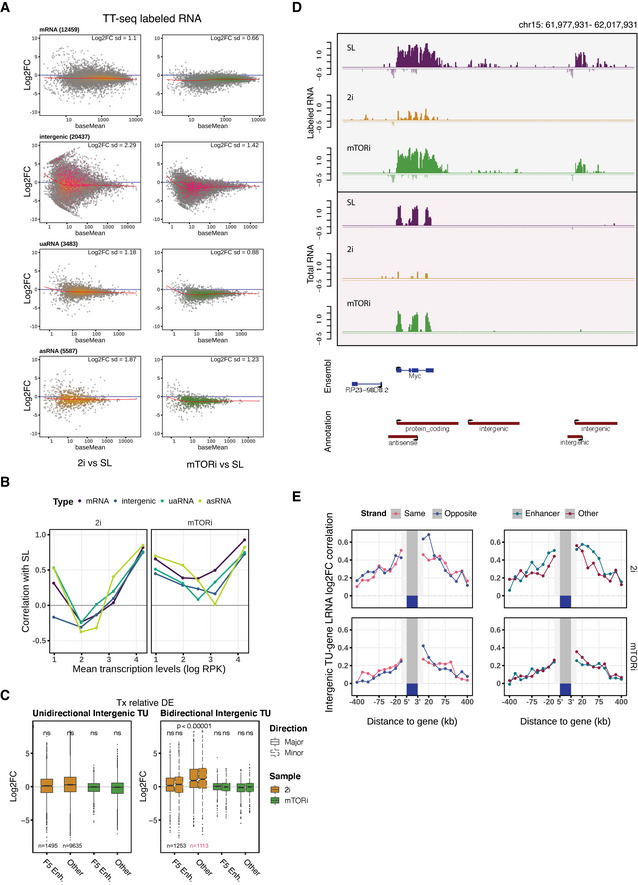
Pluripotent states transcription rewiring Spike‐in normalized MA plots contrasting 2i (2 days, yellow) and mTORi (1 day, green) to SL state. Local regression lines are in red. Bidirectional non‐enhancer intergenic TUs from (C) are highlighted in pink.Pearson's correlation of log‐labeled RNA RPK by mean transcription‐level bins in the same contrasts.DESeq2 internal normalized differential expression tests of intergenic RNA labeled RNA read counts. Major and minor TU directions are assigned by labeled RNA RPKs. FANTOM5 (Andersson *et al*, 2014) mouse enhancers are overlapped with the intergenic TUs. Boxplots are with central bands at the median, 0.25 and 0.75 quartiles box area, 1.5× interquartile range (IQR) whiskers. Statistical test is performed with two‐tailed unpaired Student's *t*‐test of log_2_ fold changes (*P < 0.00001).Spike‐in‐normalized labeled and total RNA profiles of Myc gene neighborhood (in log‐scale).Log_2_FC correlation of neighboring intergenic TUs (*n* = 14,978) and genes (*n* = 7,087) by genomic distance bins, separately for the relative strandedness to genes and the combined enhancer annotations (FANTOM5 (Andersson *et al*, 2014), ChromHMM (Pintacuda *et al*, 2017), and STARR‐seq (Peng *et al*, 2020)).

Next, we performed differential expression analysis by counting the labeled RNA reads on mRNA, intergenic RNA, uaRNA and asRNA TU intervals, and compared 2i and mTORi to SL conditions after spike‐in normalization (Fig 2A). The early transitioning from SL to mTORi was associated with a homogeneous down‐regulation of both mRNA and ncRNA synthesis (Fig 2A, right), while individual mRNAs and intergenic RNAs showed a highly variable response with both up‐ and down‐regulation in 2i 2‐day adaptation (Fig 2A, left). mRNA synthesis remained largely correlated between SL in mTORi condition, irrespective of initial expression level, while non‐coding transcripts were more variable (Fig 2B, right). In contrast, 2i induced a greater extent of rewiring for both mRNAs and ncRNAs (Fig 2B, left). We wondered if the changes amongst non‐coding TUs followed a dicernable pattern. By large, intergenic TUs transcription log_2_FC followed mRNA TUs; however, bidirectional TUs, lacking clear enhancer status, appeared to be able to “escape” the global down‐regulation in the 2i transition (Fig 2A and C). We identified the absence of active chromatin states and low evolutionary conservation as a unique characteristic of these refractory bidirectional TUs (Fig EV3I and J).

Because we observed a more facetted transcriptional response to 2i transition than mTORi (Fig EV3H), we wondered whether transcriptional changes of intergenic TUs followed a discernable mechanism. Myc gene expression is known to be strongly attenuated under 2i treatment (Marks *et al*, 2012; Galonska *et al*, 2015) but is maintained in mTORi (Bulut‐Karslioglu *et al*, 2016). Interestingly, we found the same trends on neighboring intergenic TUs (Fig 2D). Globally, intergenic TU transcription changes correlated well with transcription changes of neighboring genes in a direction‐ and proximity‐dependent manner (Figs 2E and EV3K and L). From the ± 100 kb gene neighborhood, the highest correlation was observed for transcription changes within the 20‐kb region downstream antisense, indicating that convergent transcripts to protein‐coding genes experienced stronger co‐regulation (Fig 2E, left). With gene expression covariance analysis of a single‐cell dataset (Kolodziejczyk *et al*, 2015), we confirmed the transcription positioning influence to be preserved in the SL–2i transition, and also observed correlation of expression between downstream convergent gene pairs (Fig EV3M). In the 2i transition, transcriptional changes at protein‐coding genes correlated slightly better with transcriptional changes at intergenic TUs overlapping an enhancer state than at other intergenic TUs (Fig 2E, top‐right). This is in line with recent studies demonstrating that hard wiring of promoter–enhancer interactions, thus, coordinated changes in transcriptional activity, explains changes in RNA‐seq abundance between SL and 2i states for many differentially regulated genes (Joshi *et al*, 2015; Novo *et al*, 2018; Atlasi *et al*, 2019). In contrast, mTORi induced less locally coordinated changes (Fig 2E, bottom). In summary, our analysis shows that ncTU‐gene position dependent co‐regulation is an important contribution of intergenic transcription variation in pluripotent state transitions.

### Estimating elongation velocity with TT‐seq and Pol II coverages

**Figure EV4:**
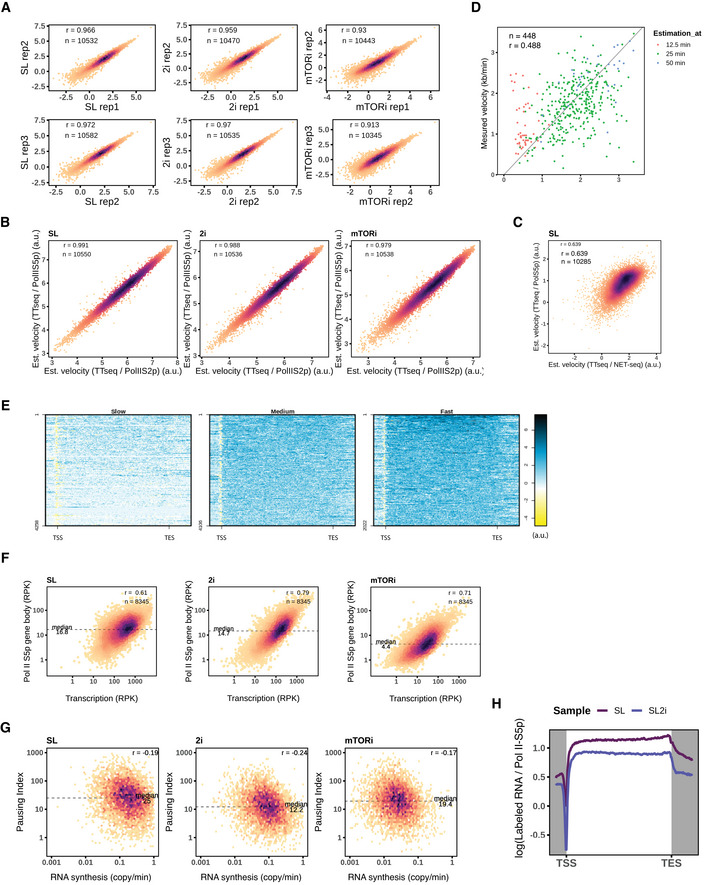
Pluripotent states transcription dynamics profiles MINUTE‐ChIP Pol II S5p gene occupancy correlation by replicates. Spearman's rank correlation coefficients are indicated.Pearson's correlation between Pol II S5p estimated gene velocity and Pol II S2p estimated velocity.Pearson's correlation between Pol II S5p estimated gene velocity and NET‐seq estimated velocity. NET‐seq signal density was calculated from gene exons.Comparison of the published elongation velocities at the specified time points (Jonkers *et al*, 2014) and recalculated multi‐time‐point velocity estimates by linear regression (Materials and Methods). Pearson's correlation is shown.K‐means grouped estimated elongation velocity gene coverages are plotted in log scale. Upstream 2 kb and downstream 4 kb are extended from Ensembl protein‐coding gene intervals.Scatter plots of Pol II S5p gene body occupancy and TT‐seq‐labeled RNA reads density. Pearson's correlation is performed on the log scale.Pausing indexes by Pol II S5p compared to RNA synthesis rates for each pluripotent condition. The medians of pausing indexes and Pearson's correlation coefficients are indicated.Mean ratio between normalized TT‐seq‐labeled RNA and Pol II S5p coverages of SL and SL2i cells with 10,674 genes. A separate batch of Pol II S5p MINUTE‐ChIP samples is used.

**Figure 3:**
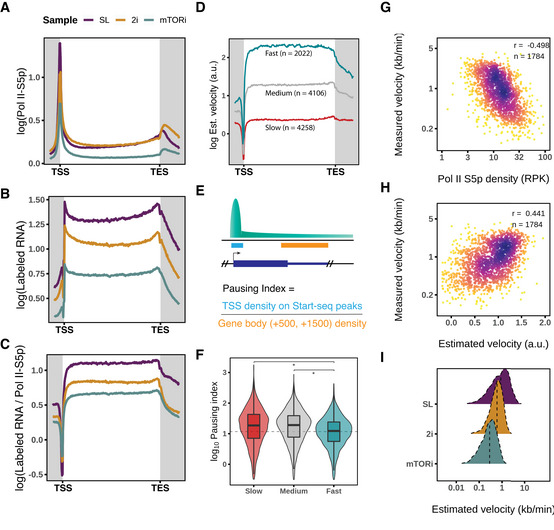
Elongation velocity estimation demarcates transcription dynamics in the pluripotent states A–CQuantitative MINUTE‐ChIP mean coverage profiles of Pol II S5p, spike‐in‐normalized TT‐seq‐labeled RNA, and their ratio as the estimated elongation velocity on 10,447 genes.DK‐means grouped average profiles of estimated velocity in the SL condition.EIllustration of pausing index calculation (Materials and Methods).FPausing indices of Pol II S5p by different elongation velocity groups in the SL condition (two‐tailed unpaired Student's *t*‐test *P < 2.2e‐16). Boxplots are with central bands at the median, 0.25 and 0.75 quartiles box area, 1.5× interquartile range (IQR) whiskers; outliers are hidden.GCorrelation of GRO‐seq measured elongation velocity (Materials and Methods) and Pol II S5p gene body occupancy.HCorrelation of GRO‐seq measured velocity and estimated velocity by TT‐seq‐labeled RNA and Pol II S5p MINUTE‐ChIP ratio.IRidge distribution of estimated elongation velocity of the pluripotent states. Medians are shown as vertical dashed lines.

TT‐seq‐labeled RNA coverage allows estimation of productive transcription initiation frequencies (Gressel *et al*, 2017), which is defined as the number of Pol II molecules that initiate and release into productive elongation, per unit of time. Meanwhile, Pol II ChIP‐seq captures the Pol II occupancy (average density in a cell population) on the DNA template, which is a function of the number of polymerases and their elongation velocity (Ehrensberger *et al*, 2013). Thus, by combining Pol II occupancy measurements with TT‐seq derived initiation frequencies, Pol II elongation velocities can be estimated (Caizzi *et al*, 2021). During transcription initiation, TFIIH complex catalyzes Pol II S5p (CTD Serine‐5 phosphorylation) to recruit RNA 5′ end capping enzyme (Søgaard & Svejstrup, 2007; Glover‐Cutter *et al*, 2009; Hsin & Manley, 2012); therefore, Pol II S5p ChIP signal corresponds to the fraction of productive Pol II amongst the total chromatin‐engaged Pol II molecules (Steurer *et al*, 2018). Therefore, we used the ratio of TT‐seq density over Pol II S5p density from native MINUTE‐ChIP collected under the same three conditions, SL, 2i, and mTORi as a proxy for relative transcription elongation velocity (Materials and Methods). We resorted to MINUTE‐ChIP for a quantitative profiling of Pol II S5p densities across the three conditions. MINUTE‐ChIP is a multiplexed method in which chromatin from multiple samples is barcoded, pooled, and immunoprecipitated in a single workflow, allowing direct quantitative comparisons (Kumar & Elsässer, 2019). We barcoded triplicate samples of each condition, yielding highly reproducible genome‐wide profiles (Fig EV4A). Integrating TT‐seq and MINUTE‐ChIP profiles, we estimated elongation velocities of 10,386 protein‐coding genes (Materials and Methods, Table EV1). The estimated elongation velocities agreed well when alternative measurements of productive Pol II occupancy (Pol II S2p MINUTE‐ChIP and total Pol II NET‐seq) were used (Fig EV4B and C). In the TT‐seq‐labeled RNA/Pol II S5p profile, a deep minimum after the TSS indicated promoter‐proximal pausing (Muse *et al*, 2007; Steurer *et al*, 2018; Bartman *et al*, 2019); a flat gene body ratio demonstrated the steady elongation velocity; and our transcription velocity estimation also recapitulated the “getting up to speed” model toward elongation termination (Fig 3A–C) (Jonkers *et al*, 2014; Jonkers & Lis, 2015).

Next, we closely examined the estimated velocity in SL, and used k‐means to classify the estimated elongation velocity profiles of 10,386 protein‐coding genes into three groups with slow, medium, and fast elongation velocity (Figs 3D and EV4E). Acceleration of elongation velocity toward the TES (transcript end site) was only observed for the slow and medium velocity groups (Fig 3D). Beyond the TES, the fast elongation group showed a steeper decline of transcription velocity (Figs 3D and EV4E). We wondered if the velocity groups were indicative of Pol II promoter‐proximal pausing. With the derived pausing intervals from Start‐seq (Dorighi *et al*, 2017), we calculated the pausing index (PI) as a ratio of Pol II S5p density within the pausing interval versus the gene body (Fig 3E, Materials and Methods). Comparing PIs of slow and medium velocity genes, we confirmed that promoter‐proximal pausing was less prevalent in the fast elongating gene group (Fig 3F).

Next, we sought to validate and calibrate our elongation velocity estimates with direct measurements. Absolute elongation velocities have previously been determined in mESC by inhibiting pause‐release using a Cdk9 inhibition (flavopiridol) followed by a GRO‐seq time course (Jonkers *et al*, 2014). In this study, performed under SL condition, velocities for 1,197 transcripts were reported; and we were able to extract velocities for a total of 1,944 transcripts by reanalyzing the dataset using our annotation and model fitting with at least two available time points (Materials and Methods). Our recalculated velocities agreed with the published velocities with minor discrepancies due to different linear models applied (Fig EV4D, Materials and Methods), confirmed a reverse relation with our Pol II S5p density (Fig 3G), and agreed with our estimated elongation velocities to a reasonable degree of correlation (Fig 3H). Using the absolute velocity measurements as reference, we were also able to scale our estimated velocities to the reference unit (kb/min). Thus, the labeled RNA/Pol II S5p ratio provides a transcriptome‐wide elongation velocity estimate. These results also highlight the close connectivity of productive Pol II occupancy with its determinants, initiation frequency, and elongation velocity (Figs 3G and EV4F).

### Interpretation of elongation velocity in mouse ES cells

**Figure 4:**
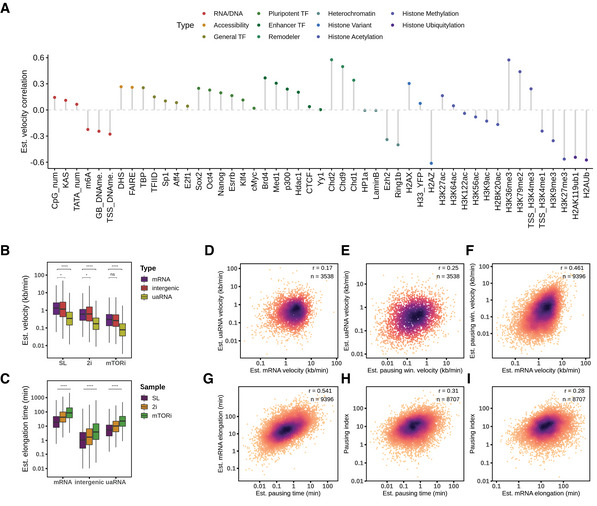
Transcription elongation velocity interpretation APearson's correlation of estimated gene elongation velocity (*n* = 10,611) with sequence and chromatin features (Materials and Methods).BEstimated velocity scaled by GRO‐seq measured velocity plotted across the culture conditions and TU types. Boxplots are with central bands at the median, 0.25 and 0.75 quartiles box area, 1.5× interquartile range (IQR) whiskers; outliers are hidden. *P*‐values are tested with two‐tailed unpaired Student's *t*‐test in log scale.CEstimated velocity scaled by GRO‐seq measured velocity plotted across the culture conditions and TU types and estimated elongation time from the respective TU lengths. Boxplots are with central bands at the median, 0.25 and 0.75 quartiles box area, 1.5× interquartile range (IQR) whiskers; outliers are hidden. *P*‐values are from a two‐tailed unpaired Student's *t*‐test in log scale.D–FEstimated velocity correlation between mRNA, paired uaRNA, and mRNA TSS pausing window (by Start‐seq peaks). Estimated elongation dynamic parameters are in SL condition, the same as below.G, HCorrelation of estimated pausing time (on Start‐seq peaks) with gene body elongation time and pausing index.ICorrelation between estimated mRNA gene body elongation time and pausing index.

To understand the control of elongation velocity, we first sought to correlate elongation velocity in SL condition with other epigenomic features. Many active histone modifications have been found to be associated with elongation velocity (Jonkers *et al*, 2014; Veloso *et al*, 2014), for instance, H3K36me3 and H3K79me2. Using our estimation, we confirmed that these two active marks positively correlated with elongation velocity, and in addition identified a positive correlation with several chromatin remodelers (Chd1, Chd2, and Chd9; Fig 4A). Notably, a recent Cryo‐EM structure implicates Chd1 in clearing the nucleosome barrier in front of the RNA polymerase traversing (Farnung *et al*, 2021). Moreover, we observed anti‐correlation with polycomb repressive marks (H3K27me3, H2Aub, Ezh2, and Ring1b), and also histone variant H2A.Z, which has been shown to be negatively associated with pause‐release (Mylonas *et al*, 2021) (Fig 4A).

We observed Pol II to travel faster through mRNA and intergenic TUs than uaRNA TUs (Fig 4B). Deriving TU elongation time estimates as elongation velocity divided by TU interval length, we found that Pol II spends the longest time traversing through mRNAs, followed by uaRNAs and intergenic TUs (Fig 4C). When looking at pairs of uaRNA and mRNA arising from a shared promoter region, we found a weak correlation in estimated elongation velocity and time (Fig 4D and E).

Next, we wondered if Pol II pause‐release dynamics were connected to elongation velocity or RNA synthesis rate. Transcription initiation frequency is known to weakly anti‐correlate with promoter‐proximal pausing (Sanchez *et al*, 2018; Gressel *et al*, 2019). Accordingly, we observed this trend consistently in all the three conditions (Fig EV4G). Intriguingly, pausing time, which corresponds to the time Pol II needs to travel through the promoter‐proximal pausing interval, also showed good correlation with gene elongation time in SL (Fig 4F and G). Further, we also confirmed that the pausing index moderately correlated with pausing time and mRNA elongation time (Fig 4H and I).

### Transcription velocity changes in mESC pluripotent states

**Figure EV5:**
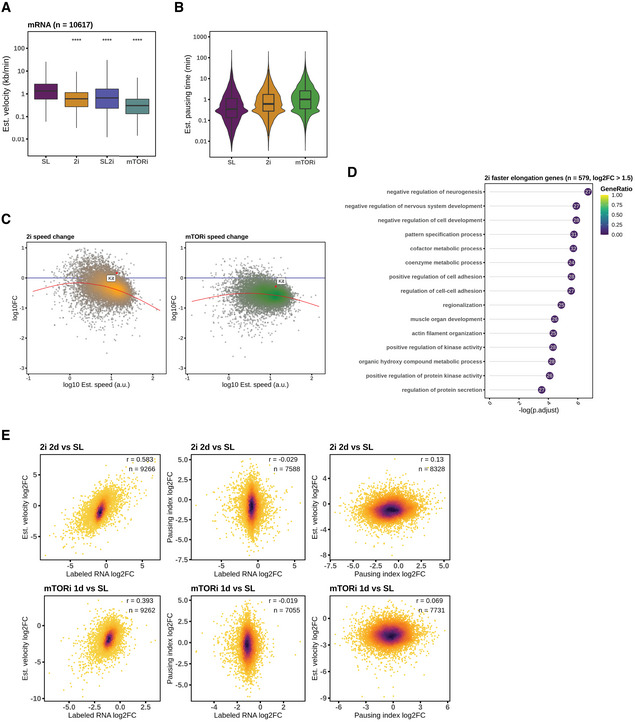
Transcription dynamics changes in aspect of elongation velocity Distribution of estimated elongation velocity of the pluripotent states after scaling with experimentally measured velocity, the same for (B). Boxplots are with central bands at the median, 0.25 and 0.75 quartiles box area, 1.5× interquartile range (IQR) whiskers; outliers are hidden. Two‐tailed unpaired Student's *t*‐test is applied (****P < 0.0001).Estimation of pausing time in STAR‐seq TSS intervals by the pluripotent states. Boxplots are with central bands at the median, 0.25 and 0.75 quartiles box area, 1.5× interquartile range (IQR) whiskers; outliers are hidden.MA plots of elongation velocity changes in 2i and mTORi conditions against estimated velocity in SL condition. Local regression lines were appended to illustrate the trend of changes. The *Kit* gene, known to be involved in proliferation and self‐renewal, is highlighted as an example of velocity change relative to global trends after state transitions.Gene ontology biological processes of top 579 genes with increased elongation velocity in 2i transition.Comparisons of the changes of RNA synthesis, elongation velocity, and Pol II pausing index in 2i and mTORi transition.

We expanded our elongation velocity estimation to 2i and mTORi states, and found 2.2 and 3.7 folds decrease in median elongation velocities relative to SL cells (Figs 3I and EV5A). With 2i supplement in serum‐LIF medium, SL2i cells confirmed that the decrease in elongation velocity was an effect of MEK/GSK3β inhibition (Figs EV4H and EV5B). Moreover, 2i cells showed a preferential reduction in fast‐elongated genes, while mTORi cells slowed down more homogeneously (Fig EV5C). For the small fraction of genes with increased velocity in 2i cells, we observed gene ontology (GO) enrichment in negative regulation of cell development (Fig EV5D). In line with the global transcription velocity reduction in 2i and mTORi states, the average pausing times increased from 21 (SL) to 37 (2i) and 61 (mTORi) seconds in the pausing interval of a median length 52 bp (Fig EV5B).

### Transcription termination is associated with elongation velocity

**Figure 5:**
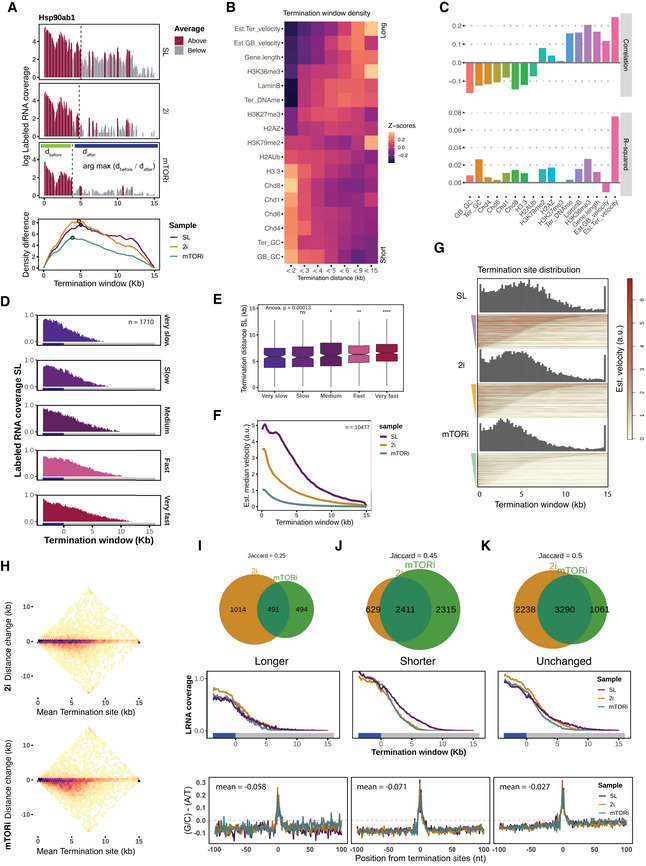
Transcription termination extension with elongation velocity AIllustration of termination site detection algorithm on gene Hsp90ab1, by maximizing the read density contrast as shown in the sliding curves (bottom; Materials and Methods). The main termination sites are indicated by dashed lines and solid points for the three cell states.BGene and chromatin feature occupancy in the called termination windows which are binned by termination distances. Density values are log‐normal transformed and standardized to z‐scores.CCorrelation of termination distances with individual chromatin features (upper); multivariate linear regression decomposed R‐squared values for the termination distance explanation (lower; Materials and Methods).DLabeled RNA median coverage in the potential termination window ordered by externally measured elongation velocity (Jonkers *et al*, 2014).ETermination distances by GRO‐seq measured elongation velocity (Jonkers *et al*, 2014), with the median sizes from slow to fast are 5.9, 5.8, 6.1, 6.3, and 6.5 kb in SL condition. Boxplots are with central bands at the median, 0.25 and 0.75 quartiles box area, 1.5× interquartile range (IQR) whiskers; outliers are hidden. Global ANOVA test *P*‐value is indicated, and two‐tailed unpaired Student's *t*‐test was performed against the “very slow” group (*P < 0.05, **P < 0.01, ****P < 0.0001).FEstimated elongation velocity median coverage in the potential termination window by the mESC states.GTermination sites distribution (above) and estimated elongation velocity coverage heatmaps (below) by the order of termination distance in each condition.HScatter plots of termination distance changes in 2i and mTORi states with mean termination distance.I–KClasses of termination distance changes in 2i and mTORi state. Top, Venn diagrams with Jaccard overlapping index; middle, labeled RNA reads coverage from the last exon (blue box) over the potential termination window (grey box); bottom, GC versus AT nucleotide mean frequency contrast in the 100 bp flanking region around the termination sites.

**Figure EV6:**
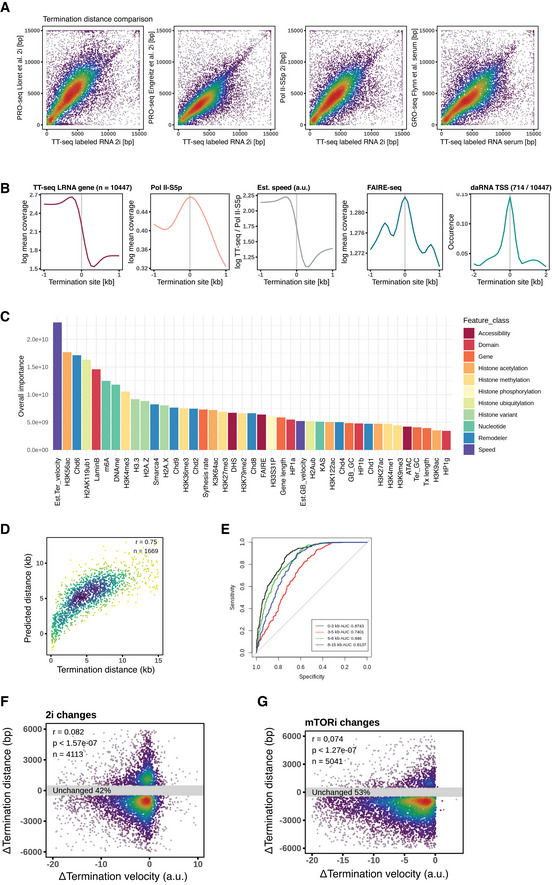
Transcription termination sites comparison and explanation ATermination distances called by TT‐seq, PRO‐seq, and Pol II S5p coverages in the potential termination window were compared by scatter plots.BAverage coverage ± 1 kb around termination sites of TT‐seq‐labeled RNA, Pol II S5p, estimated elongation velocity, FAIRE‐seq, and daRNA TSS occurrence with 10,447 protein‐coding genes.CGradient boosting machine (gbm) model's feature importance of predicting termination distance. Forty‐one genomic features are used, the same as below.DComparison of the actual termination distances and the predicted distances on the hold‐out test set by the gbm model.EA receiver‐operating characteristic curve (ROC) of showing termination distance groups prediction performance with features as described above.F, GScatter plots of elongation velocity changes and termination distance changes of 2i and mTORi transitions.

TT‐seq detects transient RNA downstream of the polyadenylation (pA) site/TES, allowing identification of transcription termination sites (TTSs) (Schwalb *et al*, 2016). To define the genome‐wide main termination sites from our TT‐Seq data, we used a one‐step global profile detection to locate the position that had the greatest difference in the density of labeled RNA before and after this site within a 15‐kb potential termination window downstream of the TES, which allows a rapid scan irrespective of the local fluctuation of read sparsity (Fig 5A, Materials and Methods). Next, we applied the same algorithm to other transcription readout methods (PRO‐seq, GRO‐seq, and Pol II S5p MINUTE‐ChIP), and found that these termination sites agreed with TT‐seq results (Fig EV6A). The observed median termination distance, defined as the interval from the TES to the determined termination site, was 5.1 kb in SL condition.

Analysis of Pol II mutants revealed that mutants with slower elongation velocity exhibited a shorter termination distance (Hazelbaker *et al*, 2013; Sheridan *et al*, 2019). To which extent the natural elongation velocity explains transcriptome‐wide termination distance has not been determined yet. Therefore, we analyzed TT‐seq‐labeled RNA coverage in the 15‐kb potential termination window downstream of the TES across groups of genes with increasing elongation velocity, and found that indeed fast elongation genes appeared to have longer labeled RNA coverages (Fig 5D), albeit genes with similar elongation velocity exhibited a considerable variation of termination distances (Fig 5E).

Hence, we wondered if elongation velocity together with other features associated with termination distance. To this end, we calculated the average velocity in the termination window of each gene and evaluated the extent of which elongation velocity versus a panel of epigenomic features can explain the termination distance. We first assumed linear responses and ranked with Pearson's correlation coefficients. Long termination distances (> 9 kb) were associated with faster estimated termination/gene body velocity, higher histone H3K36me3 occupancy, and longer gene lengths. In contrast, short termination distances (< 3 kb) were associated with histone H3.3, Chd8, Chd1 enrichment, and GC content in the called termination window (Fig 5B). A total of 25% termination distance variance could be explained by a multivariate linear model, in which the estimated termination velocity explained the largest variance of termination distance (Fig 5C). We further confirmed the first place of importance of termination velocity via a gradient boosting machine (gbm) non‐linear model, which explained 56% variance with acceptable prediction accuracy of the termination distances (Fig EV6C–E). In addition, at the termination sites (± 1 kb), we also found localization of chromatin accessibility and daRNA initiation, which suggests that antisense transcription collision may participate in the termination process for a small number of cases (Fig EV6B).

### Shorter termination distance associates with attenuated elongation velocity

To understand if altered elongation velocity in 2i and mTORi conditions also affected termination distance, we estimated termination sites in 2i and mTORi condition analogous to above, and found that slower elongation in 2i/mTORi coincided with shorter median termination distances: SL 5.1 kb, 2i 4.6 kb, and mTORi 3.9 kb. Termination sites in 2i and mTORi cells were mainly located within 5‐kb downstream of the TES (Fig 5G). Shorter termination distances matched with a higher degree of global velocity decrease in 2i/mTORi cells (Fig 5H). In line with this, the estimated elongation velocity rapidly declined in 2i/mTORi cells within 5 kb after the TES, while in SL condition, median estimated elongation velocity decreased more gradually (Fig 5F). We grouped genes according to their termination distance changes: shortening (29%; 45.2%), extending (14.4%; 9.4%), or remaining unchanged (52.9%; 41.6%), within a ± 500 bp threshold in 2i or mTORi relative to SL (Fig 5I–K). A small fraction of genes for which an extended termination distance in 2i or mTORi was called using the maximum contrast method did not show an extension of the average TT‐seq signal (Fig 5I), comparable to the unchanged termination sites in 2i/mTORi that largely overlapped (Fig 5K). Thus, this result suggests experimental noise in the extended group. On the other hand, labeled RNA coverage for genes with a shortened termination distance in 2i or mTORi dropped to background levels several kb earlier than in SL (Fig 5J).

Regardless of whether or not the termination sites shifted, a sharp local maximum in G/C richness was observed around both the old and new termination sites (Fig 5I and J). This result recapitulates the G/C‐rich motif mapped at the termination sites in K562 cells (Schwalb *et al*, 2016), and suggests a collection of termination sites to exist and respond to elongation velocity changes in a gene‐specific manner (Fig EV6F and G). Therefore, within a certain range reached by the Pol II termination velocity, sequence context was a consistent determinant for the local termination site choice.

## Discussion

With the assistance of quantitative techniques, we find that related pluripotent states of mouse embryonic stem cells exhibit markedly different global transcriptional kinetics. A global reduction of transcription serves to attenuate cell growth in the paused state (Bulut‐Karslioglu *et al*, 2016), but may also directly antagonize differentiation tendency of paused cells by limiting the accumulation of critical differentiation signaling (Cherepkova *et al*, 2016) and in a similar fashion promote mESC self‐renewal in the ground state (Stavridis *et al*, 2007; Ying *et al*, 2008; Wray *et al*, 2011). Furthermore, the correlations between total RNA and labeled RNA (Fig EV3E and F) indicate the decisive role of RNA synthesis in regulating the cellular abundance of coding RNAs and certain ncRNA types in mES cells.

Individual genes/TUs may defy the genome‐wide trend of transcriptional changes: pluripotency‐associated T‐box transcription factor *Tbx3* mRNA levels and transcription is maintained stably across the three states (Fig 1A); the *Kit* gene (encoding a receptor tyrosine kinase) transcription accelerates and expression increases in 2i state in line with its known function promoting mESC proliferation and self‐renewal (Fraser *et al*, 2013; Todaro *et al*, 2019) (Fig EV5C); and the transcription of non‐enhancer bidirectional intergenic ncTUs shows an increase in 2i cells against the global trend (Fig 2A and C). The overall transcriptional response of 2i ground state cells was mirrored when the 2i inhibitors were added to a SL culture (Fig EV3G), highlighting the important role of MEK/GSK3b signaling in antagonizing the ground state. Intriguingly, 2i‐induced transcriptional changes have been shown to occur with hardwired enhancer–promoter interactions (Atlasi *et al*, 2019; McLaughlin *et al*, 2019; Schoenfelder & Fraser, 2019), and 2i‐specific enhancer activation occurs via the loading of Esrrb upon the pre‐existing chromatin interactions (Atlasi *et al*, 2019), resulting in H3K27ac enrichment (Barakat *et al*, 2018; Schoenfelder & Fraser, 2019). However, H3K27ac *per se* appears characteristic not necessary for enhancer function (Sanchez *et al*, 2018; Zhang *et al*, 2020). In our data, intergenic ncRNAs, with or without enhancer annotations, showed a strand and distance dependent co‐regulation with neighboring genes (Fig 2E). The global rewiring of cell signaling and metabolism in the ground state, therefore, reveals the large extent of established co‐regulation of non‐coding RNAs with their adjacent genes (Figs 2C and E, and EV3K–M).

Previous estimates of transcription elongation kinetics rely on chemical inhibition and time‐series experiments, either by transcription release after the inhibitor clearance (Danko *et al*, 2013; Fuchs *et al*, 2014; Veloso *et al*, 2014), Pol II initiation/pause‐release repression (Jonkers *et al*, 2014) or RNA reporter elongation efficacy (Fukaya *et al*, 2017). Such velocity measurements still leave details of global and local elongation velocity unaddressed (Jonkers & Lis, 2015). Here, our approximation of elongation velocity provides a transcriptome‐wide measurement of several key parameters. First, we were able to estimate the unperturbed elongation velocity coverage continuously from initiation to termination, due to TT‐seq/Pol II flat gene body coverage/occupancy (Fig EV4E). Second, we were able to compare global elongation velocity across different cell states, due to the simplicity of sample normalization compared to the other velocity estimation methods (inhibitor+GRO‐seq or NET‐seq; Fig 3I). Third, we mapped the local transcription velocity in the intervals of specific transcription stages. For average expressed genes, a continuous velocity estimation might not be possible due to the relatively low coverages of TT‐seq‐labeled RNA and Pol II ChIP, but velocity estimation on the entire TU proved reliable across methods (Fig 3H). Beyond the observed association between transcription velocity and chromatin features, our quantitative strategy of transcription kinetics estimation could facilitate the future study via single‐molecule perturbation for dissecting the enigma of local transcription velocity control.

**Figure EV7:**
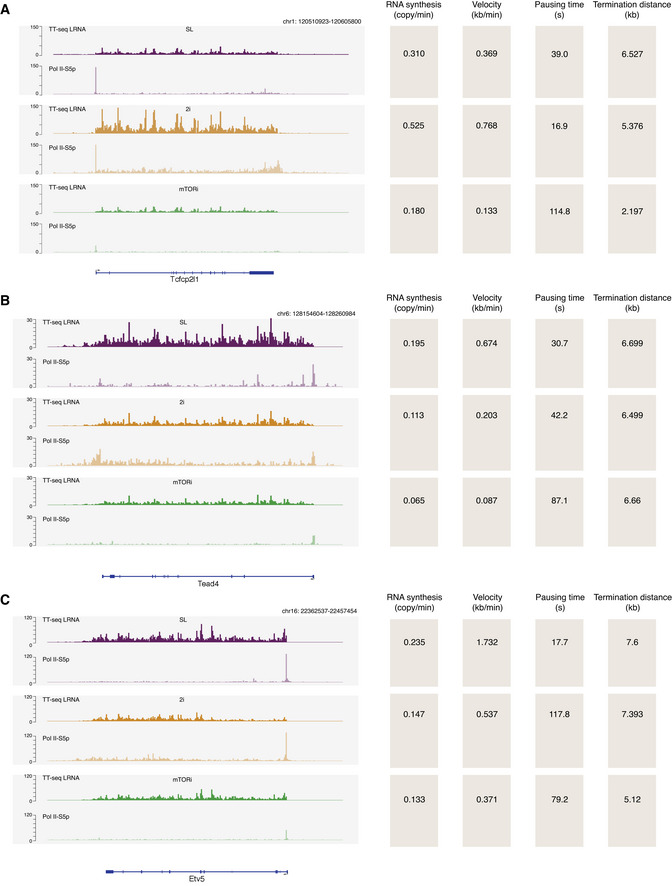
Pluripotent genes estimated transcription kinetics A–CExample genes (Tfcp2l1, Tead4, and Etv5) with the kinetic changes of RNA synthesis, elongation velocity, pausing time, and termination distance in the pluripotent states, showing with TT‐seq‐labeled RNA and Pol II‐S5p coverages in mm9 genome (in linear scale).

Finally, elongation velocity influences termination site choice to a greater extent than other chromatin features (Fig 5B and C). Through studying conditions with slower global elongation velocities, we reveal that the optimum termination site usage is responsive to elongation velocity in some genes but unresponsive in others (Fig 5J and K). Hence, termination site usage is determined by multiple factors and the exact changes of termination site usage are gene specific and may depend on GC‐rich motif occurrence in the potential termination window (Fig EV6F and G). Our observations are in line with a model where RNA Pol II traverse encounters termination roadblocks imposed by DNA sequence or antisense transcription collision, and the remaining velocity at the encounter stochastically determines if the polymerase comes to a complete stop (Figs 5J and K, and EV6B). Our model is also compatible with the “torpedo” model in which the 5′–3′ exonuclease XRN2 degrades the cleaved nascent transcript and leads to eviction of Pol II once it caught up to Pol II (West *et al*, 2004; Brannan *et al*, 2012; Nojima *et al*, 2015; Baejen *et al*, 2017). Termination site choice may underlie some stochasticity, and multiple termination sites may be used in a given instance within a population of cells (Fig EV7A–C).

In sum, our findings reveal transcription kinetics changes in the inhibitor‐induced pluripotent state transitions. Our data support a model in which inhibition of MEK/GSK3β or mTOR signaling pathways by 2i (CHIR99021/PD0325901) or mTORi (INK128) decreases global transcriptional output, total RNA abundance, RNA turnover, RNA polymerase II elongation velocity, and termination distance.

## Materials and Methods

### Reagents and Tools table

**Table:** 

Reagent/Resource	Reference or Source	Identifier or Catalog Number
**Experimental Models**		
129X1/SvJ (*Mus musculus*)	Karolinska Center for Transgene Technologies	RW4
**Antibodies**		
Rabbit anti‐RNA Pol II S5p	Santa Cruz	sc‐47701
**Chemicals, Enzymes and other reagents**		
Knockout™ DMEM	ThermoFisher	10829018
FBS	Sigma	F7524
ESGRO LIF	Sigma	ESG1107
GlutaMAX	ThermoFisher	10565018
Non‐Essential Amino Acid	Sigma	M7145
β‐mercaptoethanol	Sigma	M3148
ESGRO Complete Basal Medium	Millipore	SF002
CHIR99021	Sigma	SML1046
PD0325901	Sigma	PZ0162
INK128	Cayman	CAYM11811‐1
4‐thiouridine	Sigma‐Aldrich	T4509
TRIzol	ThermoFisher	15596018
HPDP‐Biotin	ThermoFisher	21341
Dimethylformamide	VWR	1.02937.0500
µMACS streptavidin beads	Miltenyi Biotec	130‐074‐101
DNase set	Qiagen	79254
Ovation Universal RNA‐Seq kit	NuGEN	0348
Ampure XP beads	Beckman Coulter	A63881
NextSeq® 500/550 High Output Kit v2	Illumina	FC‐404‐2005
PhiX Control v3	Illumina	FC‐110‐3001
4‐Thio‐UTP	Jena Bioscience, Saveen&Werner	NU‐1156S
Micrococcal nuclease	New England BioLabs	M0247S
Protein G magnetic beads	BioRad	161‐4023
Qubit RNA HS Assay Kit	ThermoFisher	Q32852
Qiagen miRNeasy Micro Kit	Qiagen	217084
DL‐DTT	Sigma	43815
Agilent RNA 6000 Pico Kit	Agilent	5067‐1513
Agilent RNA 6000 Nano Kit	Agilent	5067‐1511
Ethanol absolute	VWR	20821.296
2‐propanol	Sigma	I9516‐4X25ML
**Software**		
R	https://www.r‐project.org/	3.6.3
Ubuntu	https://ubuntu.com/	18.04.5 LTS
STAR	Dobin *et al* (2013)	2.7.3a
DESeq2	Love *et al* (2014)	1.24.0
Rsubread	Liao *et al* (2019)	1.34.7
bowtie2	http://bowtie‐bio.sourceforge.net/bowtie2/index.shtml	2.3.5.1
HOMER	http://homer.ucsd.edu/homer/	4.11
rtracklayer	Bioconductor	1.46.0
bamsignals	Bioconductor	1.18.0
Rsamtools	Bioconductor	2.2.3
STAN	Bioconductor	2.14.0
caret	CRAN	6.0‐88
gbm	CRAN	2.1.8
**Other**		
Illumina NextSeq 500	Illumina	
Bioruptor	Diagenode	
Bioanalyzer	Agilent	
TC20™ Cell Counter	Biorad	
Countess® Cell Counter	ThermoFisher	
Qubit	ThermoFisher	
Simpliamp thermal cycler	ThermoFisher	

### Methods and Protocols

#### Cell culture

Mouse embryonic stem cell RW4 (male, 129X1/SvJ) were cultured in 0.1% gelatin‐coated dishes with serum medium: Knockout DMEM medium with 15% FBS (Sigma, F7524), 0.1 mM ESGRO LIF (Sigma, ESG1107), 2 mM GlutaMAX (ThermoFisher, 10565018), 0.1 mM Non‐Essential Amino Acid (Sigma, M7145), 0.1 mM β‐mercaptoethanol (Sigma, M3148); 2i medium: ESGRO Complete Basal Medium (Millipore, SF002), 3 μM GSK3β inhibitor CHIR99021 (Sigma, SML1046), 1 μM Mek 1/2 inhibitor PD0325901 (Sigma, PZ0162), 0.1 mM LIF. Inhibition of mTOR was in serum‐LIF (SL) medium supplemented with 200 nM INK128 (CAYM11811‐1).

#### TT‐seq extraction protocol

TT‐seq labeling steps were performed as described before (Schwalb *et al*, 2016; Gressel *et al*, 2019) with minor modifications. In short, cells in the different pluripotent media were cultured for 1–2 days in four 15‐cm dishes. One dish was used for cell number counting, and the remaining were supplemented with 500 µM of 4‐thiouridine (4sU; Sigma‐Aldrich, T4509) for 5 min at 37°C and 5% CO2, then immediately quenched by adding TRIzol (ThermoFisher, 15596018) for RNA extraction after mixing with cell number proportional spike‐in RNAs (0.4 ng/million cells). Total RNA was fragmented to an average of 1,000 nt with Bioruptor (Diagenode), then coupled with HPDP‐Biotin (ThermoFisher, 21341) dissolved in dimethylformamide (VWR, 1.02937.0500). A small aliquot was saved as fragmented total RNA (FRNA) and the rest was purified with µMACS streptavidin beads (Miltenyi Biotec, 130‐074‐101). Purified labeled RNA (LRNA) was subjected to DNase digestion (Qiagen, 79254). Total fragmented RNA and labeled RNA libraries were prepared using Ovation Universal RNA‐Seq kit (NuGEN, 0348). The pooled library was size‐selected by Ampure XP beads (Beckman Coulter, A63881) before sequencing with NextSeq® 500/550 High Output Kit v2 (Illumina, FC‐404‐2005, 75 cycles).

#### Reads alignment and TU annotation

Paired‐end short reads were aligned to mm9 and mm10 genome references (GENCODE) by STAR 2.7.3a with setting:

‐‐outFilterMismatchNoverReadLmax 0.02 ‐‐outFilterMultimapScoreRange 0 ‐‐alignEndsType EndToEnd.

Mapped reads were subjected to transcription unit annotation as described before (Schwalb *et al*, 2016), processed in parallel by “TU filter” (R shiny app). Briefly, paired‐end reads midpoints were binned into 200‐bp genome coverage matrices by both strands (adding up the matrices if the sample had multiple replicates), then subjected to the binary hidden Markov state calling with R package “GenoSTAN” with “PoissonLog” method. Next, the active state intervals were aggregate as the raw TUs and joined by exons per gene. Non‐coding TU locations were named by their relative position to the nearby coding TUs (Fig EV3A).

TU differential expression analysis was performed by DESeq2 (1.24.0) (Love *et al*, 2014) with read counts collected from the annotated TU intervals by featureCounts (Rsubread 1.34.7) (Liao *et al*, 2019). TU correlation tests were performed with kallisto estimated tpm (transcript per million) by indexed transcript references of non‐coding TU (ncTU) genomic sequences together with GENCODE transcriptome vM21 and ERCC spike‐in sequences.

#### Spike‐in RNA design

ERCC synthetic spike‐in RNAs were used as external references for total RNA and labeled RNA sample size normalization as described before (Schwalb *et al*, 2016; Gressel *et al*, 2019), with minor modifications. Briefly, 6 pairs of spike‐in RNAs with 4sU labeled/unlabeled mixture were prepared as below:

**Table:** 

ERCC spike‐in RNA	Concentration (ng/µl)	Labeled rate (%)
Sp2 (ERCC‐00043)	1	100
Sp4 (ERCC‐00136)	0.1	100
Sp5 (ERCC‐00145)	1	10
Sp8 (ERCC‐00092)	0.1	10
Sp9 (ERCC‐00002)	1	0
Sp12 (ERCC‐00170)	0.1	0

For every million cells, 0.4‐ng spike‐in mix was added into the TRIzol (ThermoFisher, 15596018) cell lysis to eliminate technical errors retained during the steps of biotinylating, RNA purification, and library preparation.

#### Sample size estimation

We quantified GENCODE transcripts, *de novo* annotated ncTUs and spike‐in transcripts using the alignment‐free mapper (kallisto 0.46.2). For normalization, we calculated the size factors of all spike‐ins in the total transcriptome and labeled spike‐ins (2, 4, 5, 8) in the labeled transcriptome according to DESeq's method (Love *et al*, 2014). The normalized transcriptomes were subjected to size factor calculation to be presented as the relative sample sizes of both total and labeled RNA abundance (Fig 1H).

#### RNA synthesis rate estimation

The estimated labeled/total RNA read counts of GENCODE transcripts and spike‐ins from kallisto were first normalized by respective spike‐ins sizes from each sample. The gene‐level estimated counts took the sum of transcript variants. A linear model was trained only with the normalized labeled spike‐ins (Sp2, Sp4, Sp5, and Sp8) log_2_ labeled (*X*_L_) and total (*X*_F_) read counts in response to the respective label rates *r*: log_2_(*r*) ~ *X*_F_ + *X*_L_. The evaluation was performed with five‐fold cross‐validation with 10 times subsampling, and the predictions on the hold‐on set were examined. Then, a final model with spike‐in counts of all samples was trained, which resulted in an adjusted *R*‐squared 0.9917. The labeling rate was predicted from transcript's labeled/total read counts. RNA half‐life was extrapolated from the predicted labeled rate in 5 min to replacing half of the current total RNA amount. In the same way, a second model was trained to predict all spike‐in weights per cell w: log_2_(*w*) ~ *X*_F_ + *X*_L_, which resulted in an adjusted *R*‐squared 0.991. Then, the copy number per cell was transformed from weight with transcript effective length from kallisto. Finally, RNA synthesis rate (cell^−1^ min^−1^, or copy/min per cell) was calculated by multiplying labeled rate and transcript copy number.

#### MINUTE ChIP library preparation

For each growth condition (SL, 2i, and SL + mTOR inhibitor), three pellets of 1 × 10^6^ cells were collected and samples were processed according to the MINUTE‐ChIP workflow (Kumar & Elsässer, 2019). Briefly, native cell pellets were lysed and DNA was digested to mono‐ to tri‐nucleosome fragments using micrococcal nuclease (New England BioLabs,M0247S) in Lysis buffer (100 mM Tris‐HCL [pH 8.0], 0.2% Triton X‐100, 0.1% sodium deoxycholate, 10 mM CaCl_2_, and 1× PIC). Subsequently, dsDNA adaptors (containing T7 promoter, 8bp sample barcode, and a 6 bp unique molecular identifier [UMI]) were ligated onto the fragmented chromatin in the same pot supplemented with blunting and ligation reagents. Barcoded samples were then pooled and aliquoted for ChIP (saving 5% as Input) for 4 h at 4°C with Protein G magnetic beads (BioRad; 161‐4023) pre‐coupled with 5 µg of RNA Pol II S5p antibody (Santa Cruz; sc‐47701). Next, ChIP and input DNA were purified and final libraries were generated through sequential steps of *in vitro* transcription, RNA 3′ adapter ligation, reverse transcription, and PCR amplification. Post library cleanup and quality assessment, libraries were combined at 4‐nM concentration and sequenced on the Illumina NextSeq500 platform.

#### Read coverage

Short reads genome coverage was processed with R/Bioconductor packages “rtracklayer”, “bamsignals”, and “rsamtools” by piling up only the uniquely mapped and paired‐end reads with insertion size < 2 kb. Sample‐wise coverage of each genomic interval was normalized by the respective size factors. For TT‐seq sample size factors were generated from spike‐in RNA read counts; for MINUTE‐ChIP samples, scaling factors were from the respective ChIP and input sizes after combining sample replicates. In the heatmaps and coverage profile plots, each coverage vector of different lengths was resized by “spline” function to the same number of positions.

#### Pol I, Pol II, and Pol III TU classification

RNA polymerases ChIP‐seq datasets ((Jiang *et al*, 2020), GSE145791) were aligned to mm10 genome by bowtie2 v2.3.5 with “‐‐local” setting. Each TU's Pol I, Pol II, and Pol III density were divided by the sum to obtain relative occupancies, and a threshold of 90% enrichment was used for the classification indicated in the ternary plot (Fig EV2I). For validation, ChIP‐seq data of Pol III subunits PRC1, PRC4, and the cofactors BRF1, TFIII ((Carrière *et al*, 2012), E‐MTAB‐767) were aligned and subjected to peak calling with MACS2 default setting at “−*q* 0.01” cutoff, and overlapped on the ternary plot of the combined intergenic TUs (GRO‐seq, PRO‐seq, and TT‐seq).

#### Epigenome feature extraction

Promoter DNA sequences were extracted around the TSS (−1,000 to +50 nt) on the sense strand. CpG number was by CG dinucleotide, TATA number was by the “TATA” pattern. For counting ChIP‐seq signal density, TSS intervals (−500 to 500 bp) and Ensembl (GRCm38.79) gene body intervals were used, with the samples as below:

**Table:** 

Features	Types	Gene parts	Catalogue	Reference
TSS DNAme.	DNA	TSS	GSM1127953	Galonska *et al* (2015)
GB DNAme.	DNA	Gene body	GSM1127953	Galonska *et al* (2015)
DHS	DNA	TSS	GSM1014154	Vierstra *et al* (2014)
E2f1	General TF	TSS	GSM288349	Chen *et al* (2008)
TBP	General TF	TSS	GSM1816104	Langer *et al* (2016)
TFIID	General TF	TSS	GSM958503	Ku *et al* (2012)
Sp1	General TF	TSS	GSM3258754	Hartl *et al* (2019)
Aff4	General TF	Gene body	GSM749810	Lin *et al* (2011)
cMyc	Pluripotent TF	TSS	GSM2417145	Chronis *et al* (2017)
Esrrb	Pluripotent TF	TSS	GSM2417188	Chronis *et al* (2017)
Klf4	Pluripotent TF	TSS	GSM2417144	Chronis *et al* (2017)
Nanog	Pluripotent TF	TSS	GSM2417187	Chronis *et al* (2017)
Oct4	Pluripotent TF	TSS	GSM2417142	Chronis *et al* (2017)
Sox2	Pluripotent TF	TSS	GSM2417143	Chronis *et al* (2017)
CTCF	Enhancer Activity	TSS	GSM3615255	Atlasi *et al* (2019)
p300	Enhancer Activity	TSS	GSM2417169	Chronis *et al* (2017)
Hdac1	Enhancer Activity	TSS	GSM2417173	Chronis *et al* (2017)
Med1	Enhancer Activity	TSS	GSM3084070	Sabari *et al* (2018)
Brd4	Enhancer Activity	TSS	GSM3084073	Sabari *et al* (2018)
Yy1	Enhancer Activity	Gene body	GSM2645362	Weintraub *et al* (2017)
Chd1	Remodeler	Gene body	GSM1581288	de Dieuleveult *et al* (2016)
Chd2	Remodeler	Gene body	GSM1581290	de Dieuleveult *et al* (2016)
Chd9	Remodeler	Gene body	GSM1581298	de Dieuleveult *et al* (2016)
HP1a	Domain	Gene body	GSM2582363	Ostapcuk *et al* (2018)
LaminB	Domain	Gene body	GSM2579539	Poleshko *et al* (2017)
Ezh2	Heterochromatin	TSS	GSM2805185	Ardehali *et al* (2017)
Ring1b	Heterochromatin	Gene body	GSM2393579	Kundu *et al* (2018)
H2AZ	Histone	Gene body	GSM1287699	Surface *et al* (2016)
H2AX	Histone	Gene body	GSM1847704	Wu *et al* (2016)
H33 YFP	Histone	Gene body	GSM2582412	Chen *et al* (2018)
H2BK20ac	Acetylation	Gene body	GSM1874093	Kumar *et al* (2016)
H3K9ac	Acetylation	Gene body	GSM2417092	Chronis *et al* (2017)
H3K27ac	Acetylation	Gene body	GSM2417096	Chronis *et al* (2017)
H3K56ac	Acetylation	Gene body	GSM3747805	Etchegaray *et al* (2019)
H3K64ac	Acetylation	Gene body	GSM3143869	Martire *et al* (2019)
H3K122ac	Acetylation	Gene body	GSM3143871	Martire *et al* (2019)
H3K4me1	Methylation	TSS	GSM2417088	Chronis *et al* (2017)
H3K4me3	Methylation	TSS	GSM2417080	Chronis *et al* (2017)
H3K9me3	Methylation	Gene body	GSM2417112	Chronis *et al* (2017)
H3K27me3	Methylation	Gene body	GSM2417100	Chronis *et al* (2017)
H3K36me2	Methylation	Gene body	GSM3772688	Weinberg *et al* (2019)
H3K36me3	Methylation	Gene body	GSM2417108	Chronis *et al* (2017)
H3K79me2	Methylation	Gene body	GSM2417104	Chronis *et al* (2017)
H2AUb	Ubiquitination	Gene body	GSM2393583	Kundu *et al* (2017)
H2AK119ub1	Ubiquitination	Gene body	GSM2865672	Yao *et al* (2018)

Log1p‐transformed ChIP read densities were trimmed at 99.5% quantile to remove technical outliers, and standardized to normal distribution N(0, 1). Each feature explaining a particular response (e.g., termination distance, Fig 5C) was decomposed for *R* ^2^, by a multivariate linear regression through the origin.

#### Pol II pausing index

Pol II TSS pausing intervals were generated from the closest 5′ capped RNA peaks (Start‐seq (Dorighi *et al*, 2017), GSM2586572). Start‐seq reads were aligned by STAR 2.7.3a and called peaks by HOMER 4.11 (Heinz *et al*, 2010) with the following setting: findPeaks ‐style groseq ‐size 20 ‐fragLength 20 ‐inputFragLength 40 ‐tssSize 5 ‐minBodySize 30 ‐pseudoCount 1. Pausing intervals of the active genes were assigned by the closest non‐redundant capped RNA intervals, which were used for TSS peak density calculation. Pol II S5p MINUTE‐ChIP bam coverages, processed as described before (Kumar & Elsässer, 2019), were subjected to pausing interval density and gene body density extraction from (+500, +1,500 bp) interval. The resulting ratios were used as the pausing index (Fig 3F).

#### Transcription elongation velocity estimation

The time course Cdk9 inhibition GRO‐seq samples (Jonkers *et al*, 2014) were annotated by TU filter to capture ongoing transcription events. The current travel distance from gene TSS was retrieved by the nearest TU fragment annotation, and was subjected to a linear regression model in response to Cdk9 inhibition times where the slope coefficient represented reversed elongation velocity and the intersection term adjusted for response time delay. The resulting elongation velocities for 1,944 genes were used as the “measured elongation velocity” for cross‐validation. Elongation velocities *v* can also be estimated from the ratio of the number of polymerases released into elongation, as measured by TT‐seq, over the Pol II occupancy (Gressel *et al*, 2019; Caizzi *et al*, 2021). Thus, to derive “estimated elongation velocity” from our multi‐omics data, we combined TT‐seq LRNA coverage with Pol II S5p MINUTE ChIP‐seq coverage as follows:

$$\hat{v}\;\approx \;\frac{{\text{RPK}}_{\text{TT-seq}\;\text{LRNA}}}{{\text{RPK}}_{\text{Pol-II}\;\text{S5p}}}.$$

This approximation allows relative comparison between different conditions as long as the numerator and denominator terms, TT‐Seq and Pol II S5p signals, are quantitative. To this end, TT‐Seq normalization by external spike‐ins is required, and MINUTE‐ChIP quantitative scaling is carried out as described before (Kumar & Elsässer, 2019). MINUTE‐ChIP is a multiplexed ChIP method in which input chromatin is fragmented using MNase and then barcoded and pooled from the different conditions. The native ChIP is performed on an aliquot of the pooled chromatin input, and the resulting Illumina library is demultiplexed by the sample barcode, enabling quantitative comparison amongst all samples in the pool. One important advantage of MINUTE‐ChIP over a standard ChIP‐seq approach with added spike‐in for normalization (e.g., ChIP‐Rx) is that the pooled samples experience the identical IP and wash conditions, removing sample‐to‐sample and batch variation that can confound even spike‐in ChIP. A table of all estimated velocities is provided as Table EV1.

#### Calling termination sites

The potential termination windows were scanned in the 15‐kb genome interval extending from the last exon ends of each gene, before the TSS of downstream consecutive genes. TT‐seq LRNA reads coverages were piled up with only the first read in pair, scaled with respective spike‐ins, and averaged by sample replicates. To capture the tipping point of RNA synthesis as transcription passes the termination site, we determined the position that gave the maximum contrast of the log‐transformed labeled RNA densities before and after this site. In practice, TT‐seq‐labeled RNA read coverage in the potential termination window was scaled (mean = 0) and their cumulative sums from the beginning to the end of the potential termination window were calculated. The maximum position was defined as the termination site (Fig 5A).

#### Non‐linear model prediction of termination distance

We used a tree‐based gradient boosting model (gbm, R package) to evaluate the non‐linear response of termination distance with 41 chromatin features. The model training control was 10‐fold cross‐validation, and with the tuned depth 40 and 1,000 trees under 0.1 shrinkage. Test set was split by the ratio 0.2 of total cases (*n* = 8,348). The trained model of predicting numerical termination distance was applied to the test set, and compared to the actual distances (Fig EV6D). Feature importance was extracted from this model as complement to the R‐squared linear explanation (Fig 5C). We evaluated gbm performance with binned termination distance groups (3, 5, 8, 15 kb) and ROC (receiver‐operating characteristic) curves were then plotted for evaluation (Fig EV6E).

## Author contributions

**Rui Shao**: Conceptualization; Resources; Data curation; Software; Formal analysis; Validation; Investigation; Visualization; Methodology; Writing – original draft; Writing – review & editing. **Banushree Kumar**: Investigation; Methodology; Writing – original draft; Writing – review & editing. **Katja Liedschreiber**: Supervision; Investigation; Methodology. **Michael Lidschreiber**: Conceptualization; Data curation; Formal analysis; Supervision; Validation; Methodology; Writing – original draft; Writing – review & editing. **Patrick Cramer**: Conceptualization; Supervision; Funding acquisition; Validation; Project administration; Writing – review & editing. **Simon J Elsässer**: Conceptualization; Data curation; Supervision; Funding acquisition; Investigation; Methodology; Writing – original draft; Project administration; Writing – review & editing.

In addition to the CRediT author contributions listed above, the contributions in detail are:

RS and SJE conceived study. RS performed TT‐seq and BK performed MINUTE‐ChIP experiments. RS analyzed all data. KL, ML, and PC advised on TT‐seq. RS generated figures. RS and SJE wrote the manuscript with input from all authors.

## Disclosure statement and competing interests

The author declares that he has no conflict of interest.

## Supporting information



Expanded View Figures PDF

Table EV1

Table EV2

## Data Availability

Primary and processed data generated for this study have been submitted to the Gene Expression Omnibus under GSE168378 (http://www.ncbi.nlm.nih.gov/geo/query/acc.cgi?acc=GSE168378; TT‐seq) and GSE126252 (http://www.ncbi.nlm.nih.gov/geo/query/acc.cgi?acc=GSE126252; MINUTE‐ChIP). Transcription unit annotation R shiny app local workflow can be found on GitHub https://github.com/shaorray/TU_filter. Data analysis steps are also available on Github: https://github.com/shaorray/TT‐seq_mESC_pluripotency.
