# Supplementary material for: Distinct transcription kinetics of pluripotent cell states
Source: Mol Syst Biol. 2022 Jan 12;18(1):e10407. doi: 10.15252/msb.202110407 (PMC8754154; doi:10.15252/msb.202110407)
Supplement: Supplementary file 1 — Expanded View Figures PDF [file MSB-18-e10407-s004.pdf]

Expanded View Figures

Figure EV1. Reproducibility evaluation of TT-seq in the pluripotent states.

A TT-seq mRNA log RPK correlation between replicates. Spearman's rank correlation coefficient was performed, the same as below.
B TT-seq annotated ncRNA log RPK correlation between replicates.
C TT-seq-labeled RNA gene RPK correlation among wild-type mESCs in SL state. RW4 E14 is the strain used in this study (male, 129X1/SvJ), compares with TX1072 (female, C57BL/6) (Żylicz *et al*, 2019), WT26 (E14, male, C57Bl/6J) (Elsässer *et al*, 2015), and H33WT (E14, male, 129×C57Bl/6J) (Elsässer *et al*, 2015). Spearman's correlation coefficient is shown.
D–F TT-seq fragmented total RNA (FRNA) log_2FC of 2i 2d, SL2i 2d, and mTORi 1d correlations with published 2i and mTORi changes (Bulut-Karslioglu *et al*, 2016). Significantly changed genes (P.adj < 0.05) in any of our state transitions are compared ($n = 852$), the same for H.
G Comparison of DESeq2's log_2FC correlation between total RNA 2i 2 days changes with public RNA-seq data (Galonska *et al*, 2015; Finley *et al*, 2018). Both our data and the public data were counted with Kallisto and tested with DESeq2, and Pearson's correlation was performed at each degree of average *P*.adjust values in the five samples.
H PCA plot with RNA-seq log_2FC of differentially expressed genes (state specific genes), called from our ground and paused state transitions, including published studies (Marks *et al*, 2012; Galonska *et al*, 2015; Joshi *et al*, 2015; Bulut-Karslioglu *et al*, 2016; Finley *et al*, 2018).

 © 2022 The Authors

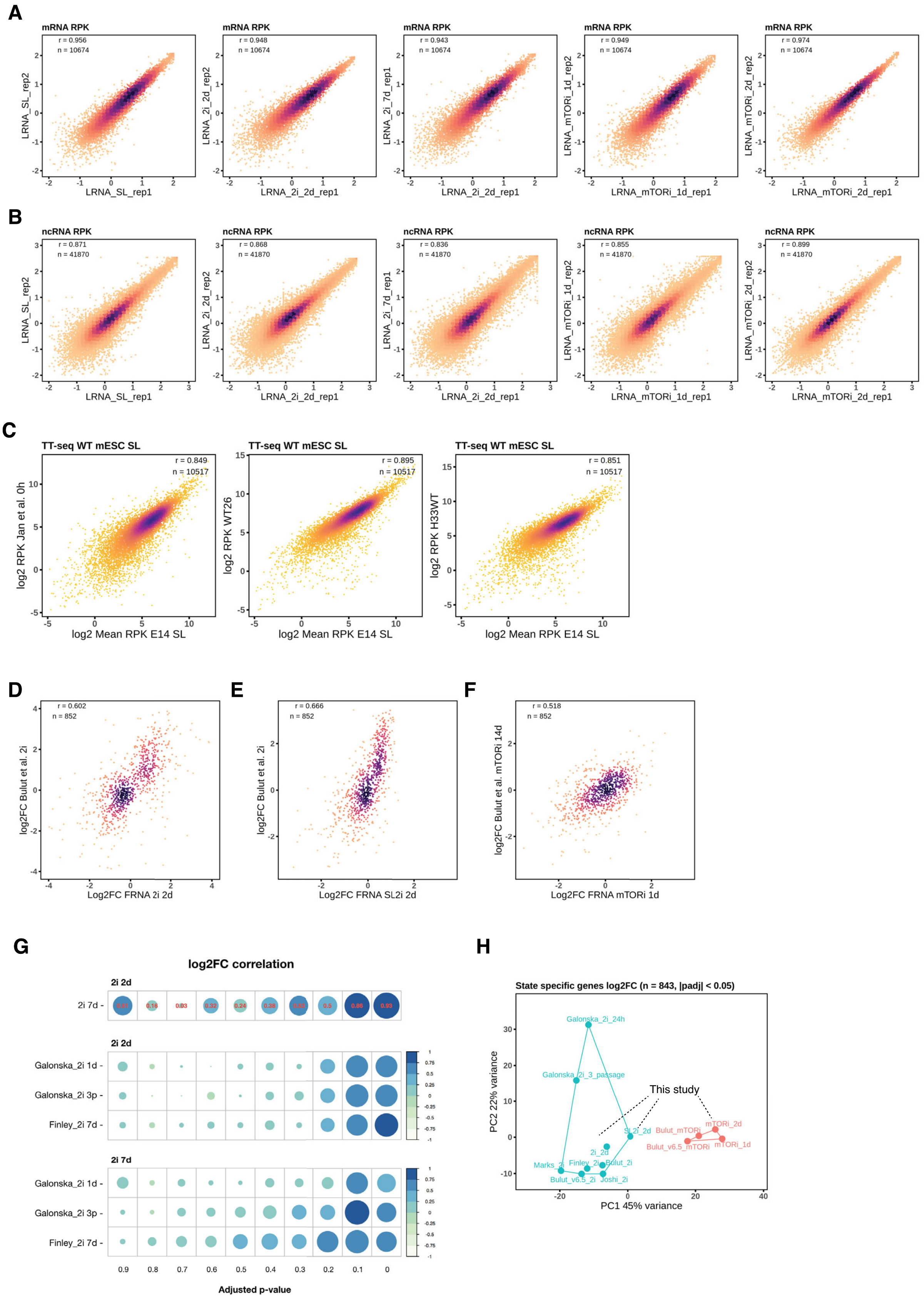
A
mRNA RPK
r = 0.956
n = 10674
LRNA_SL_rep2
LRNA_SL_rep1
r = 0.948
LRNA_2i_2d_rep2
LRNA_2i_2d_rep1
r = 0.943
LRNA_2i_7d_rep1
r = 0.949
LRNA_mTORi_1d_rep2
LRNA_mTORi_1d_rep1
r = 0.974
LRNA_mTORi_2d_rep2
LRNA_mTORi_2d_rep1
B
ncRNA RPK
r = 0.871
n = 41870
r = 0.868
r = 0.836
r = 0.855
r = 0.899
C
TT-seq WT mESC SL
r = 0.849
n = 10517
log2 RPK Jan et al. 0h
log2 Mean RPK E14 SL
r = 0.895
log2 RPK WT26
r = 0.851
log2 RPK H33WT
D
r = 0.602
n = 852
log2FC Bulut et al. 2i
Log2FC FRNA 2i 2d
E
r = 0.666
Log2FC FRNA SL2i 2d
F
r = 0.518
log2FC Bulut et al. mTORi 14d
Log2FC FRNA mTORi 1d
G
log2FC correlation
2i 2d
2i 7d
Galonska_2i 1d
Galonska_2i 3p
Finley_2i 7d
Adjusted p-value
H
State specific genes log2FC (n = 843, |padj| < 0.05)
Galonska_2i_24h
Galonska_2i_3_passage
This study
SL2i_2d
Bulut_mTORi
mTORi_2d
mTORi_1d
Bulut_v6.5_mTORi
2i_2d
Marks_2i
Finley_2i
Bulut_2i
Bulut_v6.5_2i
Joshi_2i
PC2 22% variance
PC1 45% variance

Figure EV1.

Figure EV2. Nascent RNA-seq methods comparison.

A A brief scheme of GRO-seq, PRO-seq, NET-seq, and TT-seq workflows of RNA labeling and extraction.

B Read coverages on the first and the last exons with 2-kb flanking regions and the adjacent introns (arrows). Exon and intron coverages are resized to the same dimension and plotted by log mean.

C The ratios of intron versus exon with labeled RNA reads density on Ensembl protein-coding genes across the nascent RNA-seq methods. Boxplots are with central bands at the median, 0.25 and 0.75 quartiles box area, 1.5× interquartile range (IQR) whiskers; outliers are hidden. (TT-seq1, this study; TT-seq2, Żylicz *et al*, 2019); (GRO-seq1, Flynn *et al*, 2016; GRO-seq2, Wang *et al*, 2015); (PRO-seq1, Engreitz *et al*, 2016; PRO-seq2, Lloret-Llinares *et al*, 2018); (NET-seq1, Mylonas & Tessarz, 2018; NET-seq2, Tuck *et al*, 2018); (4sU-seq1, Benabdallah *et al*, 2019; 4sU-seq2, Brown *et al*, 2017); (Bru-seq, Ardehali *et al*, 2017).

D TU annotation relative total length recovery test by TT-seq bam file random subsetting under (0.1, 0.2, 0.4, 0.6, 0.8, 1) for different TU types.

E, F Sankey plots compare the intergenic and the cis-antisense RNAs annotated by GRO-seq, PRO-seq, and TT-seq. Fractions of annotated TUs matching with public references (GENCODE (Frankish *et al*, 2021), FANTOM5 enhancer (Andersson *et al*, 2014), NONCODE (Zhao *et al*, 2016)) are indicated.

G Venn diagram of GRO-seq, PRO-seq, and TT-seq intergenic RNAs.

H RNA Pol I, II, and III occupancy (Jiang *et al*, 2020) on the total intergenic TUs. Boxplots are with central bands at the median, 0.25 and 0.75 quartiles box area, 1.5× interquartile range (IQR) whiskers; outliers are hidden. Two-tailed unpaired Student's *t*-test is performed with the common TUs against the method-specific TUs (****$P < 0.0001$).

I A ternary plot of Pol I, II, and III enrichment on the GRO-seq, PRO-seq, and TT-seq combined intergenic TU annotations. Pol III cofactors binding sites (Carrière *et al*, 2012) are pinpointed for Pol III class assignment cross-validation.

J Common overlapped and method-specific intergenic RNAs proportions with FANTOM5 enhancers (Andersson *et al*, 2014), ATAC-seq peaks (Atlasi *et al*, 2019), STARR-seq peaks (Peng *et al*, 2020), and Pol I-III occupancy in H.

© 2022 The Authors

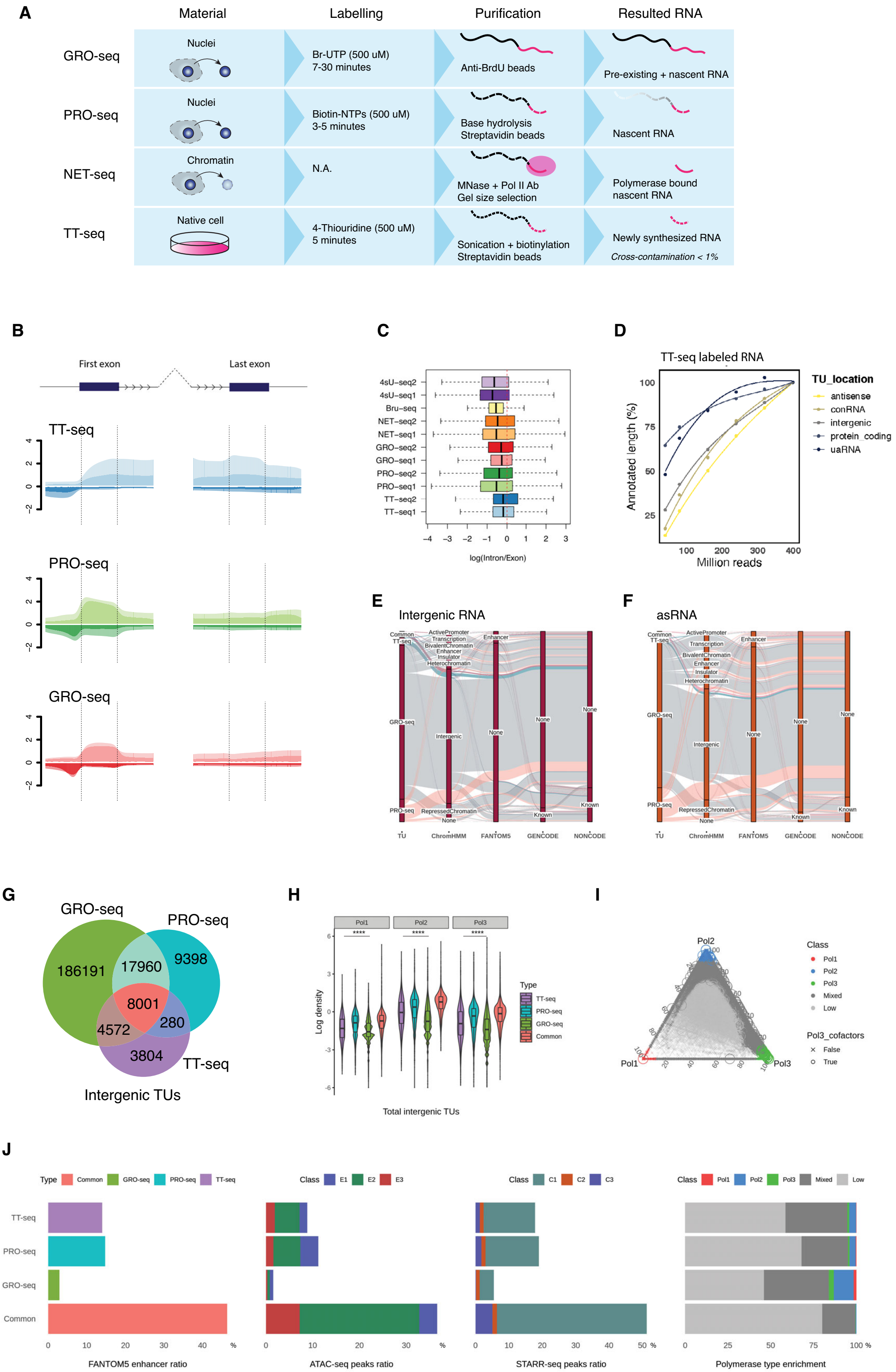
A
Material
Labelling
Purification
Resulted RNA
GRO-seq
Nuclei
Br-UTP (500 uM)
7-30 minutes
Anti-BrdU beads
Pre-existing + nascent RNA
PRO-seq
Nuclei
Biotin-NTPs (500 uM)
3-5 minutes
Base hydrolysis
Streptavidin beads
Nascent RNA
NET-seq
Chromatin
N.A.
MNase + Pol II Ab
Gel size selection
Polymerase bound
nascent RNA
TT-seq
Native cell
4-Thiouridine (500 uM)
5 minutes
Sonication + biotinylation
Streptavidin beads
Newly synthesized RNA
Cross-contamination < 1%
B
First exon
Last exon
TT-seq
PRO-seq
GRO-seq
C
4sU−seq2
4sU−seq1
Bru−seq
NET−seq2
NET−seq1
GRO−seq2
GRO−seq1
PRO−seq2
PRO−seq1
TT−seq2
TT−seq1
log(Intron/Exon)
D
TT-seq labeled RNA
Annotated length (%)
Million reads
TU_location
antisense
conRNA
intergenic
protein_coding
uaRNA
E
Intergenic RNA
F
asRNA
TU
ChromHMM
FANTOM5
GENCODE
NONCODE
G
GRO-seq
PRO-seq
TT-seq
186191
17960
9398
8001
4572
280
3804
Intergenic TUs
H
Pol1
Pol2
Pol3
Log density
Total intergenic TUs
Type
TT-seq
PRO-seq
GRO-seq
Common
I
Pol2
Pol1
Pol3
Class
Pol1
Pol2
Pol3
Mixed
Low
Pol3_cofactors
False
True
J
Type
Common
GRO-seq
PRO-seq
TT-seq
Class
E1
E2
E3
C1
C2
C3
FANTOM5 enhancer ratio
ATAC-seq peaks ratio
STARR-seq peaks ratio
Polymerase type enrichment

Figure EV2.

Figure EV3. TU annotation and transcription variation of mESC pluripotent states.

A Gene-associated ncRNA types are classified by their TSS locations relative to the gene TSS: upstream antisense RNA (−1 kb, 0), convergent RNA (0, +1 kb), cis-antisense RNA (within gene body), downstream antisense RNA (TES, TES + 1 kb), and intergenic RNA.
B Illustration of Jaccard index for annotated TUs comparison.
C Jaccard indexes of mRNA, uaRNA, asRNA, and intergenic RNA intervals similarity in pairwise comparison between each sample replicate.
D Labeled RNA purification cross-contamination rates calculated as the percentage of unlabeled spike-in reads compared to all spike-in reads in respective labeled libraries.
E Pearson's correlations between mRNAs' total and labeled $\log_2$FC in 2i 2 days and mTORi 1-day transition.
F Pearson's correlations between labeled RNA and total RNA log RPK by each TU location and pluripotent state with merged replicates and log RPK on the combined TUs, the same for H.
G Correlation between 2i and SL2i ground states changes by total (FRNA) and labeled (LRNA) mRNA $\log_2$FCs.
H Pearson's correlations between the pluripotent states by labeled and total RNA log RPK of averaged replicates.
I The coefficients of ChromHMM states are in response to the internal normalized intergenic RNA $\log_2$FC differential transcription in 2i 2-day transition. Two separate logistic regression models are trained for the unidirectional and bidirectional intergenic TUs to predict their $\log_2$FC with ChromHMM states. Each states' coefficients are plotted with the respective logistic regression confidence intervals.
J Intergenic TU promoter (−500, 200 bp) evolution conservation scores from phastCons 60way (Siepel *et al*, 2005) with the same groups as in Fig 2C (two-tailed unpaired Student's *t*-test $*P < 2.2e\text{-}16$). Boxplots are with central bands at the median, 0.25 and 0.75 quartiles box area, 1.5× interquartile range (IQR) whiskers; outliers are hidden.
K Intergenic TU intervals stacked total coverage in the ± 100 kb gene neighborhoods by relative strandedness.
L Intergenic TU intervals stacked coverage by enhancer and other states in the ± 100 kb gene neighborhoods ($n = 11{,}684$).
M Single-cell gene expression (Buettner *et al*, 2015) covariance distribution by the gene pairs of random background ($n = 4{,}000$), within a topological associated domain (TAD; $n = 3{,}250$), consecutive downstream sense ($n = 2{,}058$), 3′ ends convergence ($n = 3{,}206$), and promoter divergence ($n = 3{,}036$). Wilcoxon test is performed against the background covariance. Median covariances of each gene positioning type are indicated for both SL and 2i states.

© 2022 The Authors

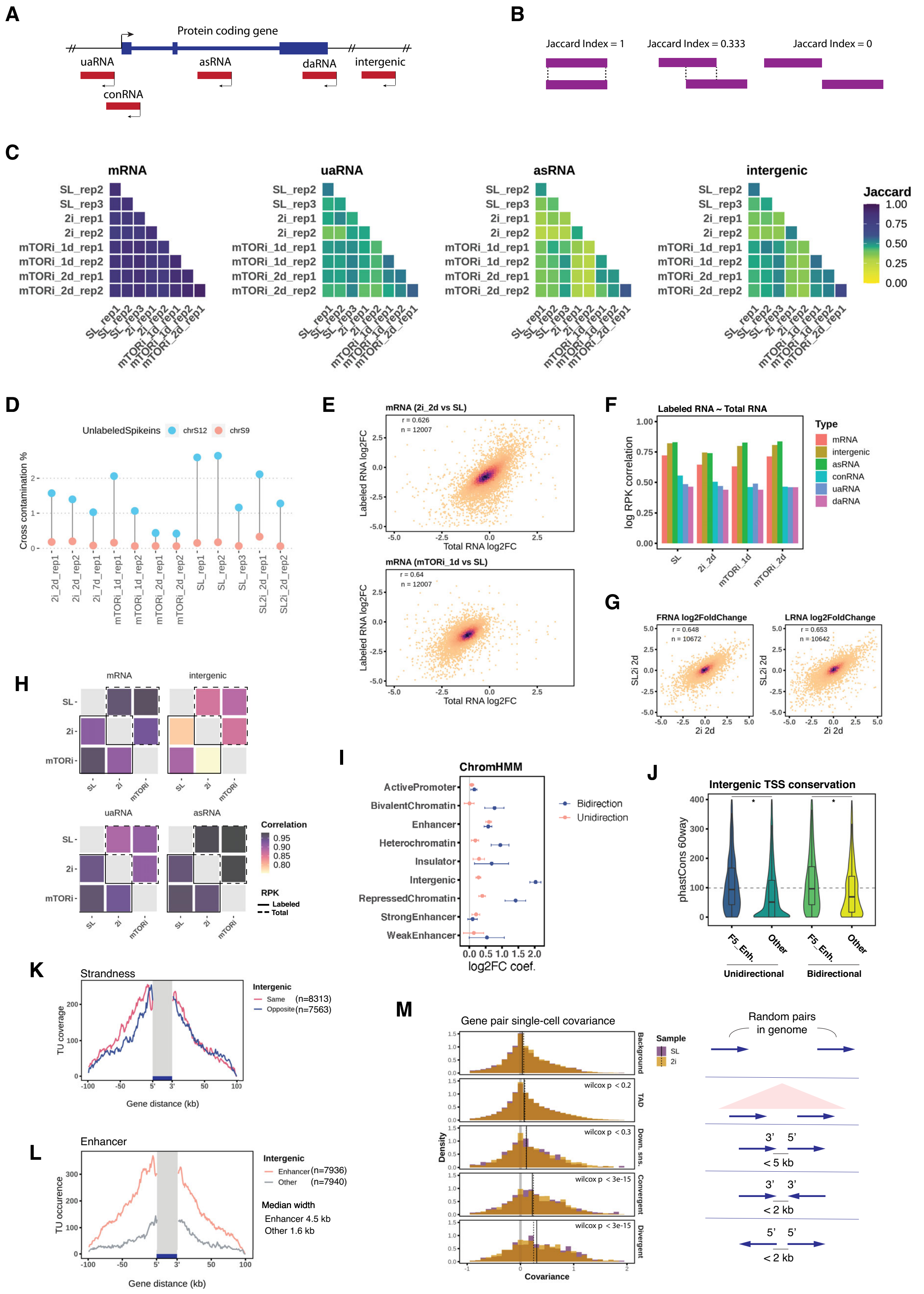
A
Protein coding gene
uaRNA
asRNA
daRNA
intergenic
conRNA
B
Jaccard Index = 1
Jaccard Index = 0.333
Jaccard Index = 0
C
mRNA
uaRNA
asRNA
intergenic
Jaccard
D
UnlabeledSpikeins chrS12 chrS9
Cross contamination %
E
mRNA (2i_2d vs SL)
Labeled RNA log2FC
Total RNA log2FC
mRNA (mTORi_1d vs SL)
F
Labeled RNA ~ Total RNA
log RPK correlation
Type
mRNA
intergenic
asRNA
conRNA
uaRNA
daRNA
G
FRNA log2FoldChange
LRNA log2FoldChange
H
Correlation
RPK
Labeled
Total
I
ChromHMM
Bidirection
Unidirection
log2FC coef.
J
Intergenic TSS conservation
phastCons 60way
Unidirectional
Bidirectional
K
Strandness
TU coverage
Gene distance (kb)
L
Enhancer
TU occurence
Median width
Enhancer 4.5 kb
Other 1.6 kb
M
Gene pair single-cell covariance
Density
Covariance
Random pairs in genome

Figure EV3.

Figure EV4. Pluripotent states transcription dynamics profiles.

A MINUTE-ChIP Pol II S5p gene occupancy correlation by replicates. Spearman's rank correlation coefficients are indicated.
B Pearson's correlation between Pol II S5p estimated gene velocity and Pol II S2p estimated velocity.
C Pearson's correlation between Pol II S5p estimated gene velocity and NET-seq estimated velocity. NET-seq signal density was calculated from gene exons.
D Comparison of the published elongation velocities at the specified time points (Jonkers *et al*, 2014) and recalculated multi-time-point velocity estimates by linear regression (Materials and Methods). Pearson's correlation is shown.
E K-means grouped estimated elongation velocity gene coverages are plotted in log scale. Upstream 2 kb and downstream 4 kb are extended from Ensembl protein-coding gene intervals.
F Scatter plots of Pol II S5p gene body occupancy and TT-seq-labeled RNA reads density. Pearson's correlation is performed on the log scale.
G Pausing indexes by Pol II S5p compared to RNA synthesis rates for each pluripotent condition. The medians of pausing indexes and Pearson's correlation coefficients are indicated.
H Mean ratio between normalized TT-seq-labeled RNA and Pol II S5p coverages of SL and SL2i cells with 10,674 genes. A separate batch of Pol II S5p MINUTE-ChIP samples is used.

© 2022 The Authors

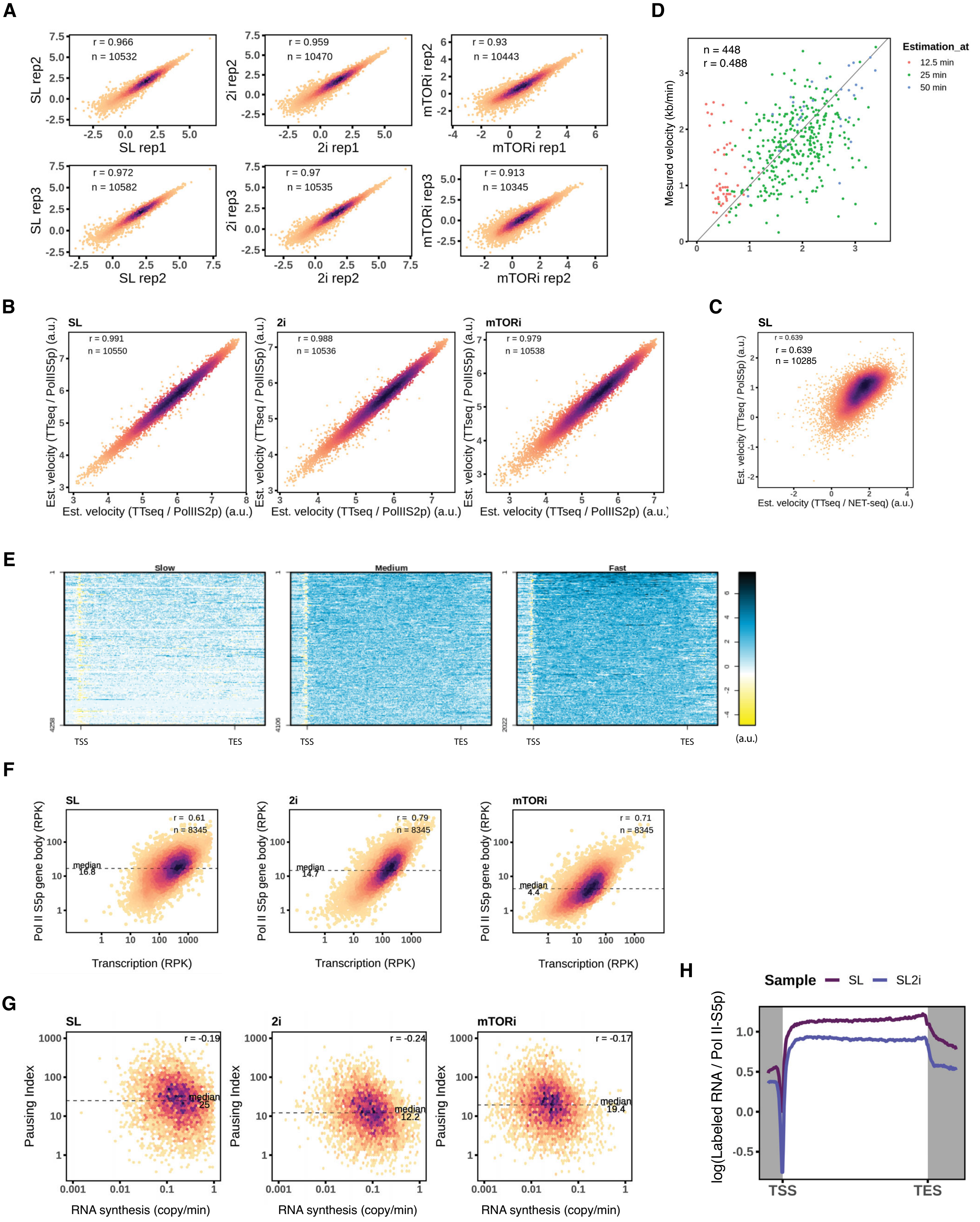

Figure EV4.

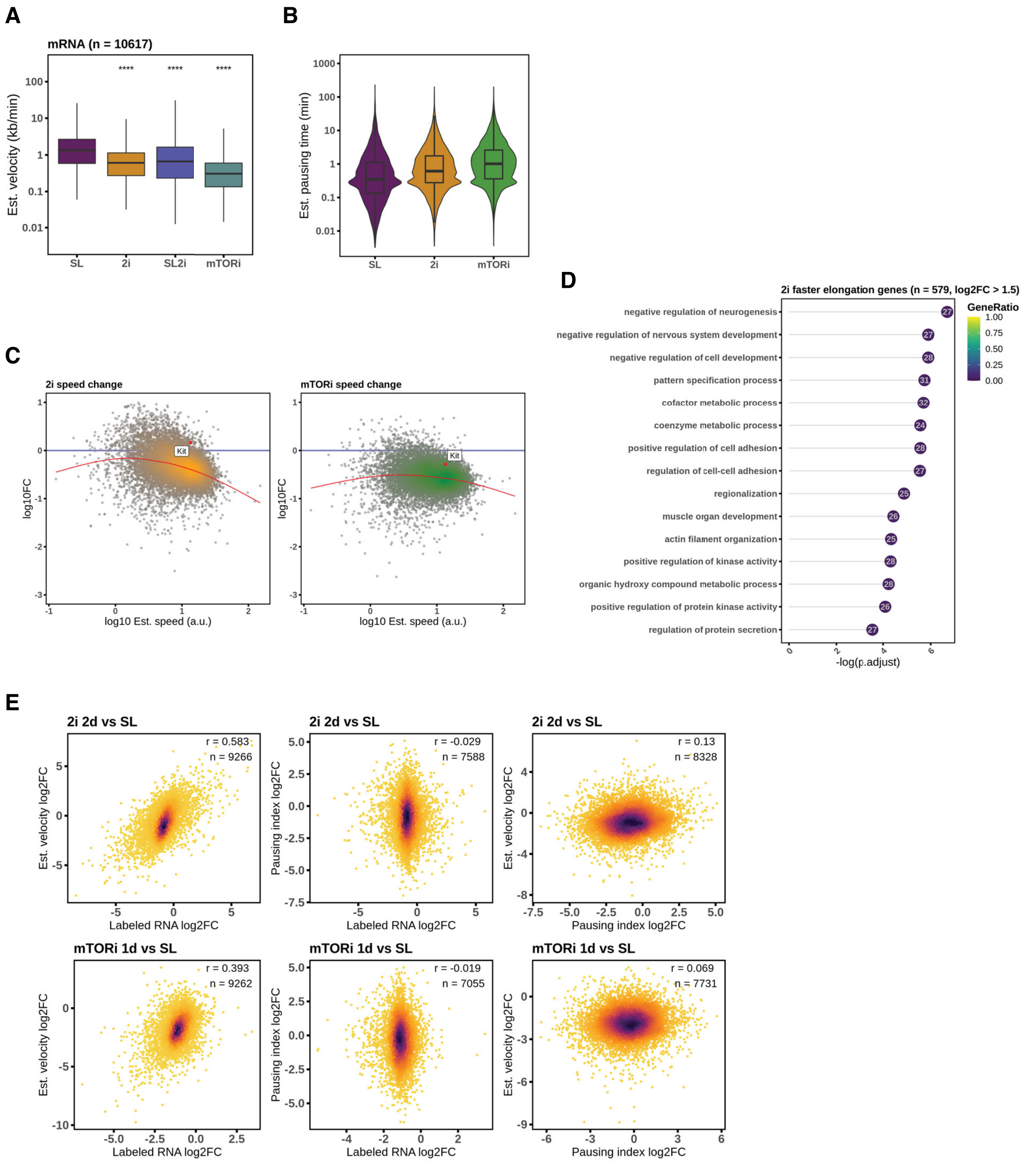

Figure EV5. Transcription dynamics changes in aspect of elongation velocity.

A Distribution of estimated elongation velocity of the pluripotent states after scaling with experimentally measured velocity, the same for (B). Boxplots are with central bands at the median, 0.25 and 0.75 quartiles box area, 1.5× interquartile range (IQR) whiskers; outliers are hidden. Two-tailed unpaired Student's *t*-test is applied (****$P < 0.0001$).

B Estimation of pausing time in STAR-seq TSS intervals by the pluripotent states. Boxplots are with central bands at the median, 0.25 and 0.75 quartiles box area, 1.5× interquartile range (IQR) whiskers; outliers are hidden.

C MA plots of elongation velocity changes in 2i and mTORi conditions against estimated velocity in SL condition. Local regression lines were appended to illustrate the trend of changes. The *Kit* gene, known to be involved in proliferation and self-renewal, is highlighted as an example of velocity change relative to global trends after state transitions.

D Gene ontology biological processes of top 579 genes with increased elongation velocity in 2i transition.

E Comparisons of the changes of RNA synthesis, elongation velocity, and Pol II pausing index in 2i and mTORi transition.

 © 2022 The Authors

Figure EV6. Transcription termination sites comparison and explanation.

A Termination distances called by TT-seq, PRO-seq, and Pol II S5p coverages in the potential termination window were compared by scatter plots.

B Average coverage ± 1 kb around termination sites of TT-seq-labeled RNA, Pol II S5p, estimated elongation velocity, FAIRE-seq, and daRNA TSS occurrence with 10,447 protein-coding genes.

C Gradient boosting machine (gbm) model's feature importance of predicting termination distance. Forty-one genomic features are used, the same as below.

D Comparison of the actual termination distances and the predicted distances on the hold-out test set by the gbm model.

E A receiver-operating characteristic curve (ROC) of showing termination distance groups prediction performance with features as described above.

F, G Scatter plots of elongation velocity changes and termination distance changes of 2i and mTORi transitions.

© 2022 The Authors

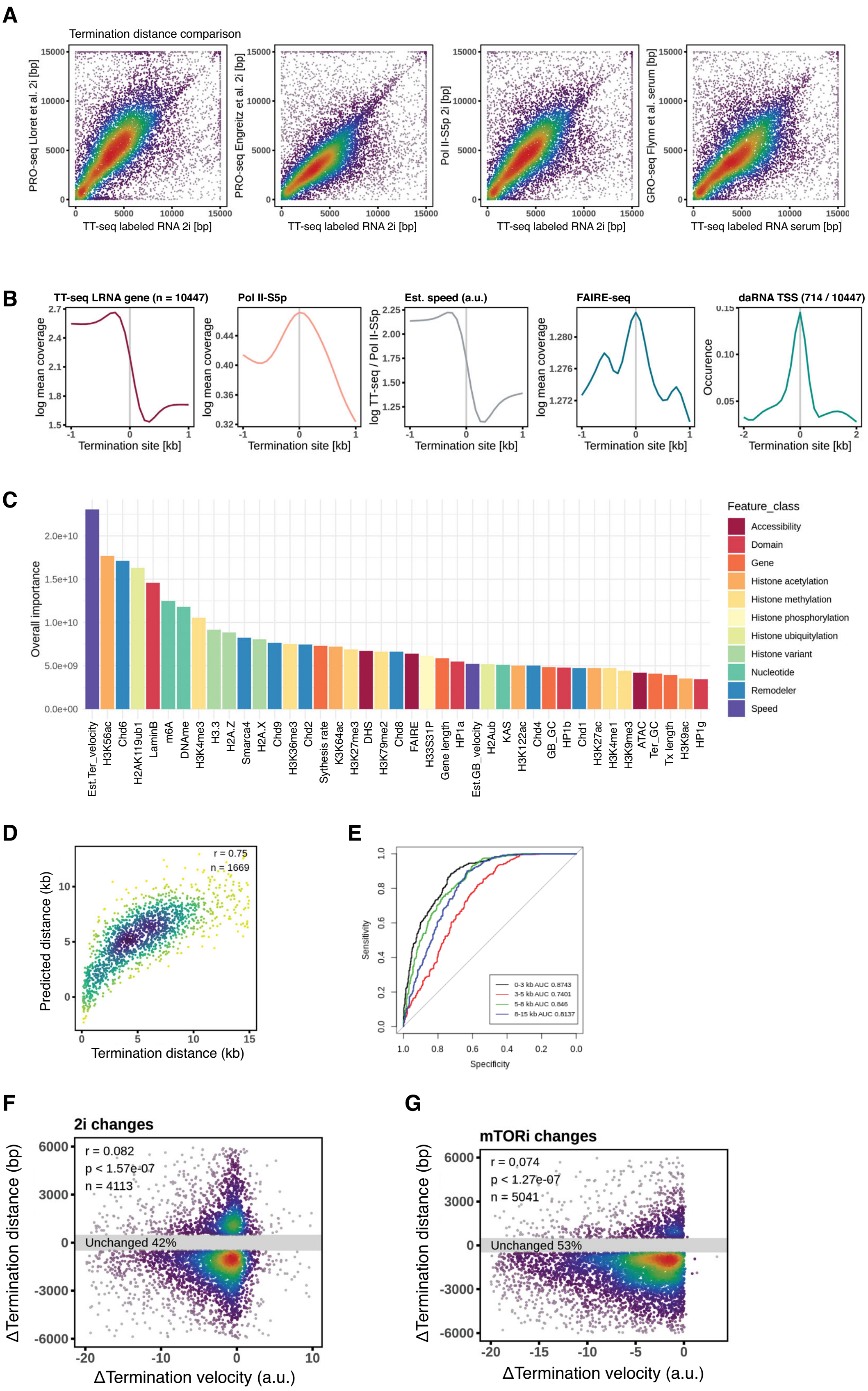

Figure EV6.

 © 2022 The Authors

Figure EV7. Pluripotent genes estimated transcription kinetics.

A–C Example genes (Tfcp2l1, Tead4, and Etv5) with the kinetic changes of RNA synthesis, elongation velocity, pausing time, and termination distance in the pluripotent states, showing with TT-seq-labeled RNA and Pol II-S5p coverages in mm9 genome (in linear scale).

© 2022 The Authors

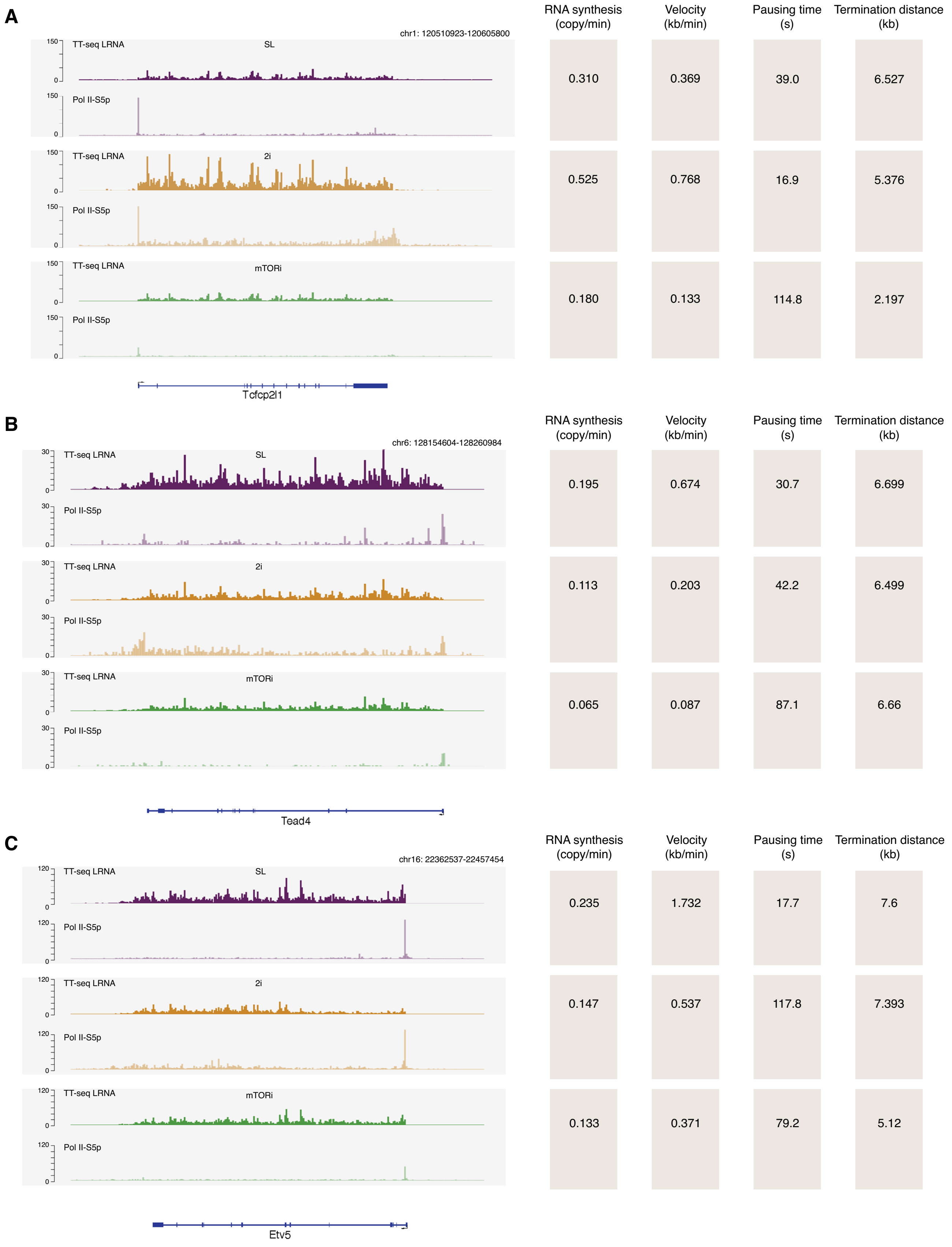

Figure EV7.

© 2022 The Authors
