# Supplementary material for: Distinct transcription kinetics of pluripotent cell states
Source: Mol Syst Biol. 2022 Jan 12;18(1):e10407. doi: 10.15252/msb.202110407 (PMC8754154; doi:10.15252/msb.202110407)
Supplement: Supplementary file 4 [file MSB-18-e10407-s003.pdf]

molecular systems biology

Distinct Transcription Kinetics of Pluripotent Cell States

Rui Shao, Banushree Kumar, Katja Liedschreiber, Michael Lidschreiber, Patrick Cramer, and Simon Elsässer
DOI: 10.15252/msb.202110407

Corresponding author(s): Simon Elsässer (simon.elsasser@scilifelab.se)

Review Timeline:

Submission Date:	10th Jun 21
Editorial Decision:	2nd Jul 21
Revision Received:	24th Oct 21
Editorial Decision:	7th Dec 21
Revision Received:	12th Dec 21
Accepted:	14th Dec 21

Editor: Maria Polychronidou

Transaction Report:

(Note: With the exception of the correction of typographical or spelling errors that could be a source of ambiguity, letters and reports are not edited. Depending on transfer agreements, referee reports obtained elsewhere may or may not be included in this compilation. Referee reports are anonymous unless the Referee chooses to sign their reports.)

Thank you again for submitting your work to Molecular Systems Biology. We have now heard back from the three referees who agreed to evaluate your study. As you will see below, the reviewers raise a series of concerns, which preclude the publication of your study in its current form.

Overall, the reviewers think that as it stands the study seems somewhat preliminary and several conclusions need to be better supported.

However, given that the reviewers acknowledge that the presented findings are potentially relevant, we have decided to offer you the chance to address the issues raised in a major revision. Without repeating all the points listed below, some of the more fundamental issues that need to be addressed are the following:

- Additional controls and further analyses are required to better support the main conclusions. As reviewer #1 mentions, this is especially important, given that most conclusions rely on a single methodology (i.e. TT-seq). The three reviewers also mention specific conclusions that need to be better supported (e.g. see reviewer #1 points #3 and #4, reviewer #2 points #1, #3, #4, #5, reviewer #3 point #5).

- Technical concerns need to be addressed and clarifications regarding the methodology and analyses need to be provided.

All issues raised by the reviewers need to be satisfactorily addressed. As you may already know, our editorial policy allows in principle a single round of major revision. It is therefore essential to provide responses to the reviewers' comments that are as complete as possible. I understand that the required revisions are quite substantive. Please feel free to contact me in case you would like to discuss in further detail any of the issues raised or if you would like to share your revision plan with me.

On a more editorial level, we would ask you to address the following points:

REFEREE REPORTS

Reviewer #1:

In the present manuscript, Shao et al. use transient transcriptome sequencing (TT-seq), an approach which relies on metabolic labeling with the nucleoside-analog 4-thiouridine (4sU), to investigate active transcription units in three distinct pluripotent states. The authors use their TT-seq data to de novo annotate active transcription units and found evidence for an attenuation of RNA synthesis in two pluripotent states (2i and mTORi) which is in line with previous observations by others. Shao et al. then use the TT-seq data in combination with RNAPII S5P occupancy data to estimate the elongation velocity of RNAPII. Genes with an increased estimated elongation velocity correlated with a longer transcription termination read-through, whereas genes with an attenuated estimated elongation velocity correlated with a shorter termination read-through length.
Overall this manuscript is hard to read and a clear logical line connecting the different findings is missing. Although some of the results of this study are of potential interest for researchers in the transcription field, the findings are mostly descriptive and in parts just confirm previous observations. Conclusions are not always supported by the data that is provided.

Major concerns:

(1) A limitation of this study is that most conclusions rely almost exclusively on one data type, obtained by TT-seq. Although correlations between specific subsets of transcription units are provided in the supplement (Fig. EV2C), a general correlative analysis between biological replicates in order to assess the reproducibility of the data is missing.
Furthermore, the authors draw quantitative conclusions from their TT-seq data. However, this relies on the assumption that the labeling efficiency of the newly synthesized RNA and the enrichment of the labeled RNA was high. Unfortunately, quality controls that would support this assumption are lacking. Providing additional controls would be particularly important here since the TT-seq method was originally developed and optimized for standard human cell lines including K562 cells that are easier to handle and probably also easier to label with 4sU as compared to mES cells used in the present study. Along these lines, since main conclusions rely on comparisons of TT-seq data between three pluripotent states the author should provide more evidence that the labeling efficiency was similarly high between all three conditions. Although the authors provide some evidence that the fraction of spliced transcripts is similar in all states (Fig. 1H), this is not the same as to directly measure the labeling efficiencies for all states.
Fig. 1H: please clarify if the differences between the 4 different states that are shown in this figure are indeed not significant?

(2) The way how the authors estimated the elongation velocity raises several questions. The calculation of velocity relies on RNAPII S5P ,MINUTE ChIP-seq data'? How this data was generated and the correlation between replicate data sets is not clear. What is the difference between MINUTE ChIP-seq and standard ChIP-seq applications? Did the authors use spike-in controls in their MINUTE-ChIP-seq experiments? I bring this up because variations in the RNAP S5P ChIP-seq data sets can impact the velocity calculation.
The spatial resolution of TT-seq (nucleotide resolution) and ChIP-seq strongly differs. How does this impact the velocity estimation?
Another potential concern is that RNAPII S5P is most reliably detected at the 5'-region of genes. S5P signals are significantly lower and are less reliably detected across the gene-body with the consequence that fluctuations between measurements can have stronger impact on the velocity estimation in these later elongation stages. Why have the authors not also included S2P ChIP-seq data which is more established to resemble the elongating form of RNAPII?

(3) I have several comments regarding the potential link between the elongation velocity and the 'transcription termination readthrough'. The authors use TT-seq data to define transcription termination sites using a method that 'determines the maximal density contrast'. This method should be briefly described in the main text since not every reader is familiar with this approach. The authors claim that 'fast elongation genes had longer read-through distances'. However, the differences in the observed read-through distances between some of the groups, for instance between the medium and fast groups (Fig. 5D) appear marginal. What is the actual median size of the termination window for the different gene groups shown in Fig. 5D? Are these differences significant?
In this context the authors also state: 'Thus, our data supports a model in which Pol II encounters termination roadblocks imposed by DNA sequence, and the remaining velocity at the encounter stochastically determines if the polymerase comes to a complete stop.' This statement is purely speculative and should be transferred to the discussion section.
Furthermore, the authors state 'In the model described above, G/C-rich motifs thus provide roadblocks to RNA polymerase II that lead to a final termination if the velocity is already sufficiently reduced.' This statement is based on correlative data but presented as a causal relationship and therefore should be softened.

(4) The authors claim 'that RNA synthesis decrease in the pluripotent state transitions is independent from elongation slowdown and neither controlled by pause release dynamics (Fig 3L, Fig EV3B).' Based on the data that is provided this conclusion appears as too premature and should be softened or removed.

Minor points:

(1) The manuscript contains many typos.
(2) The font sizes in several Figures are small and are hard to read.

(3) The distribution of estimated elongation velocities for all detected genes should be given.
(4) Fig. EV4A: how many genes are included in this analysis? The N-value should be added. What genes were included here? A clarification would be helpful.

Reviewer #2:

In their manuscript entitled "Newly synthesized RNA Sequencing Characterizes Transcription Dynamics in Three Pluripotent States" Shao et al. use transient transcriptome sequencing (TT-seq) - a previously described technique that allows measuring both nascent and total RNA - to distinguish gene expression features of mouse embryonic stem cells (mESCs) that were grown in three different culture conditions. These conditions are serum with leukemia inhibitory factor (LIF) (SL), serum-free in presence of LIF and inhibitors against MAPK signaling and GSK3 (2i) and serum with inhibition of mTOR signaling (mTORi). While SL and 2i represent the two most widely used approaches to propagate mESCs in an undifferentiated state, mTORi induces a general metabolic slow-down resulting in adaptation of a state reported to resemble embryonic diapause, a feature of murine development. After some useful validation experiments, including the comparison of TT-seq with published data generated with other approaches capturing nascent transcripts, the authors present data that clearly demonstrate that different culture conditions have profound effects on the abundance of different classes of transcripts via affecting both synthesis and turnover. In particular, they show that the previously reported reduction in total RNA in mTORi is due to a general, not locus-specific transcript reduction while the reduced RNA abundance in 2i appears locus-specific. In addition, they provide evidence for local co-regulation of transcript abundance and use TT-seq together with an optimized PolII ChIP-seq approach to compare elongation velocity and its impact on transcription termination in the different conditions. Overall, the experiments in this manuscript are well-designed and the data is well-analyzed. Also, due to their developmental flexibility and self-renewal capacity, mouse pluripotent cells represent an excellent model to study the impact of signaling and other factors on transcriptional regulation. However, while TT-seq as used by the authors clearly has the potential to identify novel molecular features of this important cell type, in my opinion the current manuscript should be further improved in this respect. I have listed my specific comments for consideration by the authors below.

1. The finding of locus-specific effects on transcript abundance in 2i is intriguing, but apart from some speculative statements the authors do not further investigate the mechanisms that underlie this observation. For example, can ectopic expression of MYC rescue the abundance of specific transcripts as the authors appear to apply? Similarly, are the observed effects downstream of MAPK inhibition, GSK3 inhibition or due to the absence of serum? A set of straightforward culture experiments could help clarifying these questions and further support the authors' claim in the Abstract that "signaling pathways ...immediately control key parameters of transcription".

2. Are there any genes that violate the slower elongation trend in 2i? If so, what are these genes?

3. The manuscript currently provides no validation of the success of inhibitor treatment, i.e. it remains uncertain that their cells indeed exhibit canonical features of SL, 2i and mTORi. Marker genes such as those identified by Bulut-Karslioglu et al 2016 and others might be helpful in this respect.

4. With respect to the experiments linking dilution by cell division to RNA turnover -Fig1G is hard to interpret without knowing what "absolute" confluency these cell densities correspond to. Representative images would be helpful. Also, without replicates, the conclusion that cell density caused the apparent differences in RNA turnover (instead of an unknown variable between the experiments) remains unconvincing. Why is rep3 SL budging the trend?

5. All the experiments seemed to be conducted with a single ESC line. It would be helpful to confirm key findings (such as those shown in Fig. 2A) with at least a second line, ideally on a different genetic background.

Minor comments
6. The authors describe SL as a "primed" condition, which is misleading since this nomenclature is commonly reserved for epiblast-stem cells (EpiSCs), which are grown in absence of LIF. Both SL and 2i are naïve conditions, with 2i often described as ground state.
7. A brief description on the principles of TT-seq and how they compare to other similar techniques would be helpful for the (non-expert) reader.
8. A brief discussion on how the molecular differences observed by the authors' might be resolved during differentiation (or how they might impact upon differentiation capacity) would be interesting.
9. Many figures have components that are too small to be easily read (examples are log2FC values in Fig. 2A).
10. The authors write in the Discussion "Spurious transcription ....may be an effect of the global CpG hypermethylation observed in 2i". I believe they might have meant to say hypomethylation.
11. The authors' claim that their experiments in addition to studying different cell states also provide insight into transitions between these states. Considering that the earliest timepoints assessed are 2 days (for SL-to-2i) or 1 day (for SL-to-mTORi) this might represent an overstatement and should be adjusted.
12. Typo "steadt" instead of "steady" in first paragraph of Results section

Reviewer #3:

Summary of findings

This manuscript presents a very comprehensive computational analysis that examines the relationship between signaling pathways, chromatin features and transcriptional dynamics in mouse embryonic stem cells (ESCs) including coding and non-coding transcriptional units. Experimentally, the authors measure nascent transcripts using TT-seq, steady state RNA levels and Pol II ser5p localization using MINUTE-ChIP in three different different pluripotency states of ESCs by changing culture conditions namely: naive (2i), primed (serum/LIF) and paused (mTORi). Their analysis suggests that RNA synthesis predominantly controls transcriptional output in these states and can explain the global RNA reduction between serum/LIF and 2i/mTORi conditions. They further present TT-seq with Pol II ser5p localization as an appropriate means to quantify transcription velocity which relates to termination read-through and is in turn attenuated in the naive and paused states.

This study highlights the importance of measuring transcriptional dynamics in different pluripotency states and contributes to understanding the interplay between signaling pathways, chromatin rearrangements and transcriptional control.

Major comments:

1. Overall the significance of studying transcriptional dynamics in the different pluripotency states should be emphasized throughout the manuscript. The authors put a lot of effort into integrating chromatin states in their analysis but this is not reflected in the abstract.
2. The authors nicely demonstrate the robustness of using TT-seq to measure RNA synthesis and validate it by comparing it to other methods used in ESCs. It would make the paper more accessible to the reader if the differences between these methods are introduced or perhaps explained via a schematic depiction especially for TT-seq. It would also be helpful for the reader to explain the complementarity between these methods. e.g: the use of GRO-seq and START-seq in Figure4 or the combination of TT-seq and NET-seq? Also, the MINUTE-chip method details are missing.
3. More information on the biology of the three ESCs pluripotency states would also help frame the significance of the research, in particular why is it important to study these states and how transcriptional dynamics changes reflect stemness? Also, the rationale behind the timing for the inhibitors in relation to cell cycle: 2 days, 7days for the 2i condition versus 1 & 2days for the mTORi condition needs more explanation. A PCA analysis of the unlabeled RNA (total RNA-seq) side by side with the originally characterized transcriptomes of naive and paused states would also be helpful to validate the culture conditions/timing.
4. It would be important to discuss whether the observed RNA synthesis dynamics in relation to chromatin regulation and RNA stability are features specific to the pluripotency state? Along these lines: are there any trends between cell identity genes and transcription velocity / read-through(Figure 3&5)? Also, since the argument is that RNA synthesis is the driver of total RNA levels in ESCs, is there a relationship between velocity and steady-state RNA levels on a gene-by-gene basis or when considering groups of fast, medium and slow genes(Figure 3&4)?
5. The authors state that "a stranded TT-seq signal provides an excellent demarcation of the nascent transcription unit and polymerase-independent measure of transcriptional activity" Since authors measure how elongation velocity affects transcriptional termination sites and readthrough, it would be interesting to implement PolII occupancy in their coding TU discovery pipeline. Similarly, what is the occupancy of either PolI, PolII and PolIII over bidirectional intergenic TUs that show upregulation in the naive condition? And how does their estimated velocity correlate with neighboring coding TUs in primed versus naive conditions?
6. The change in local chromatin features between pluripotency states is intriguing. It would be good to discuss the possibility to use TT-seq and elongation velocity measurements in the context of inhibiting chromatin modifiers or remodelers.

Minor comments
1. How do the authors handle dead cells during the naive and paused pluripotency state conversion? This could affect RNA measurements.
2. The relevance and correlation of the transcriptional elongation reference with cdk9 inhibition GRO-seq time course should be better explained/introduced.
3. The model in Figure EV6 is not referenced/explained in the main text.

Keywords:

mESC pluripotency, TT-seq, transcription unit, RNA kinetics, elongation velocity

"Stand first text"

Pluripotency renewal at different levels of transcription kinetics, depicting underneath programs of RNA turnover, non-coding transcripts, elongation velocity, and transcription termination.

"Bullet points"

- TT-seq annotates transcription units in mESC pluripotent states.
- Transcription reduction in the early state transitions delineates lower total RNA abundance and turnover rates.
- Estimated elongation velocity connects with RNA Pol II pause-release and termination distance.

"synopsis image"

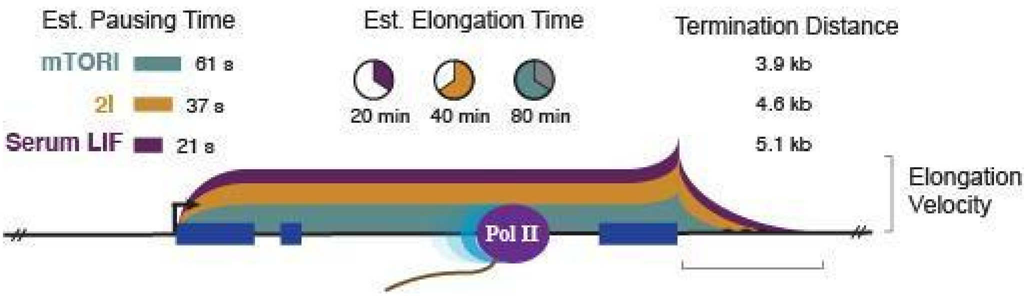

Reviewer #1:

In the present manuscript, Shao et al. use transient transcriptome sequencing (TT-seq), an approach which relies on metabolic labeling with the nucleoside-analog 4-thiouridine (4sU), to investigate active transcription units in three distinct pluripotent states. The authors use their TT-seq data to de novo annotate active transcription units and found evidence for an attenuation of RNA synthesis in two pluripotent states (2i and mTORi) which is in line with previous observations by others. Shao et al. then use the TT-seq data in combination with RNAPII S5P occupancy data to estimate the elongation velocity of RNAPII. Genes with an increased estimated elongation velocity correlated with a longer transcription termination read-through, whereas genes with an attenuated estimated elongation velocity correlated with a shorter termination read-through length.

Overall this manuscript is hard to read and a clear logical line connecting the different findings is missing. Although some of the results of this study are of potential interest for researchers in the transcription field, the findings are mostly descriptive and in parts just confirm previous observations. Conclusions are not always supported by the data that is provided.

We thank the reviewer for the comments and suggestions, we have reworked the manuscript text substantially to make it more clear and approachable. We have harmonized the terminology as below

a. transcription dynamics -> transcription kinetics
b. read-through window -> termination window
c. read-through velocity -> termination velocity
d. read-through (length) -> termination distance

We have provided more methodological details in main text and methods section, and hope to have clarified the rationale behind each analysis. We have removed some figures (seed details below) and discussions where deemed not essential for the manuscript. Following reviewer suggestions, we have also removed or toned down some conclusions.

Major concerns:

---

(1) A limitation of this study is that most conclusions rely almost exclusively on one data type, obtained by TT-seq. Although correlations between specific subsets of transcription units are

provided in the supplement (Fig. EV2C), a general correlative analysis between biological replicates in order to assess the reproducibility of the data is missing.

We have now added the correlation of TT-seq coding and non-coding TU RPKs between replicates in Appendix Fig S1A-B; and MINUTE-ChIP Pol II S5p replicate correlations in Appendix Fig S1G. Correlation and thus reproducibility is very good.

Furthermore, the authors draw quantitative conclusions from their TT-seq data. However, this relies on the assumption that the labeling efficiency of the newly synthesized RNA and the enrichment of the labeled RNA was high. Unfortunately, quality controls that would support this assumption are lacking. Providing additional controls would be particularly important here since the TT-seq method was originally developed and optimized for standard human cell lines including K562 cells that are easier to handle and probably also easier to label with 4sU as compared to mES cells used in the present study. Along these lines, since main conclusions rely on comparisons of TT-seq data between three pluripotent states the author should provide more evidence that the labeling efficiency was similarly high between all three conditions. Although the authors provide some evidence that the fraction of spliced transcripts is similar in all states (Fig. 1H), this is not the same as to directly measure the labeling efficiencies for all states.

We agree that efficient and consistent metabolic labeling, as well as clean isolation of labeled RNA, is key for RNA synthesis measurements and dependent biological conclusions. We have thus performed a series of quality controls with regard to the TT-seq experiments:

First, we confirmed that Bioanalyzer traces from the labeled RNA were consistent between all the conditions even if the amount of labeled RNA was much reduced e.g. in mTORi conditions (Author Response Figure 1A).

Second, we used labeled RNA splicing rates to confirm that our TT-seq assays are consistent with published TT-seq in mESC ("TT-seq1": our data, "TT-seq2": published data (Żylicz *et al*, 2019)) (Fig EV1B).

Third, we calculated the cross-contamination rates, that is the amount of unlabeled spike-in RNA (sp9 and sp12) reads present in the labeled TT-seq samples, since their non-zero measurement in the labeled library (Author Response Figure 1B-C). Our measurements show that unlabeled RNA average cross-contamination rate to be less than 1% of the labeled RNA (Fig EV2F).

Fourth, we calibrate the labeling rates against an external standard of spike-in RNA with defined labeling rates (0%, 10% 100%). Estimated mRNA turnover (around 60 minutes) which we derived from the labeling rates agrees well with previous estimates from TT-seq in K562 cells (Schwalb *et al*, 2016). Further, mESC TT-seq half-lives correlate with RNA decay half-lives determined by SLAM-seq and TimeLapse-seq (Author Response Figure 1D-E).

To address the reviewer's question, we now added an additional analysis that assesses labeling efficiency in all three states, confirming that they are similar:

4sU has a known mis-pairing frequency with C at a low frequency, leading to a ~10% T to C mutation frequency during reverse-transcription (Herzog *et al*, 2017). We could confirm the existence of T to C conversion in our samples: the most frequent conversion found in our labeled RNA libraries is T->C (Author Response Figure 1F-G). It is important to consider however that the Illumina platform introduces a considerable number of sequencing errors, hence it is important to confirm that the 4sU-induced mutations can be detected above the random error rate. Since total RNA contains only a negligible fraction of 4sU, we can use the total RNA samples as controls for precisely quantifying Illumina sequencing error rates. We hence analyzed mutation rates in both total and labeled RNA libraries: among the single nucleotide conversions, T->C and A->G conversions were the most abundant types in both labeled and total RNA samples (Author Response Figure 1H-I), thus attributed in large to Illumina sequencing errors. Comparing the conversion frequency of labeled to total RNA, we found a 1.1-fold enrichment of T->C conversion frequency in labeled RNA (Author Response Figure 1J). The overrepresentation of T->C conversions suggests that they arise from 4sU mis-pairing in labeled RNA. The 1.1-fold enrichment was stable across the pluripotent states (Author Response Figure 1K). Therefore, the 4sU labeling efficiency is a consistent parameter across the cell states.

Although unsaturated 4sU labeling (e.g. 90%) increases the chance of missing some newly synthesized RNA molecules, our external spike-ins allow sample size comparison within the same labeling time. Since our results are consistent with the bulk RNA synthesis measurements reported by Bulut-Karslioglu et al., we believe that labeling efficiency is not a confounding factor in our measurements.

Fig. 1H: please clarify if the differences between the 4 different states that are shown in this figure are indeed not significant?

We have added splicing rate p-values and adjusted the corresponding text in the results section: compared to serum LIF cells, 2i 2days and mTORi 1day are less spliced and mTORi 2 days spliced more with a p value < 0.05 (Fig 1I). However, the effect size is small and it is not clear if the differences are attributable to subtle differences in actual splicing rate depending on the cell state or technical variation.

---

(2) *The way how the authors estimated the elongation velocity raises several questions. The calculation of velocity relies on RNAPII S5P ,MINUTE ChIP-seq data'?*

Yes, we let the read counts of RNA Pol II S5p represent the occupancy of productive Pol II at a certain genomic position.

How this data was generated and the correlation between replicate data sets is not clear. What is the difference between MINUTE ChIP-seq and standard ChIP-seq applications? Did the authors use spike-in controls in their MINUTE-ChIP-seq experiments? I bring this up because variations in the RNAP S5P ChIP-seq data sets can impact the velocity calculation.

The MINUTE-ChIP method, including detailed experimental protocol and data analysis pipeline has been described previously (Kumar & Elsässer, 2019). It is a multiplexed ChIP method in which input chromatin is fragmented using MNase and then barcoded and pooled from the different conditions. The native ChIP is performed on an aliquot of the pooled chromatin input, and the resulting Illumina library is demultiplexed by the sample barcode, enabling quantitative comparison amongst all samples in the pool. One important advantage of MINUTE-ChIP over a standard ChIP-seq approach with added spike-in for normalization (e.g. ChIP-Rx) is that the pooled samples experience the identical IP and wash conditions, removing sample-to-sample and batch variation that can confound even spike-in ChIP.

Initially, we quality-checked public ChIP-seq datasets of total Pol II, Pol II S2p and Pol II S5p (Ferrai *et al*, 2017; Zhang *et al*, 2018; Sabari *et al*, 2018), but we observed large variance of Pol II occupancy on coding genes by pairwise correlations, which could be due to: 1

antibody, 2 ChIP protocol, 3 cell strain background, 4 growth conditions, and 5 protocol/batch-specific effects. Hence, we agree with the reviewer that traditional ChIP-Seq is problematic, but the approach we used here enables us to perform exact quantification while minimizing technical variation.

The data generated here stems from aliquots of the same pool as published (Kumar & Elsässer, 2019), hence all the QC performed there are also applicable for the current data. We have further added a correlation plot for the MINUTE-ChIP Pol II S5p replicates as Appendix Fig S1G.

We now explain the MINUTE-ChIP assay in more detail in the Methods section and also briefly in the main text, highlighting that it allows for quantitative comparisons between samples.

The spatial resolution of TT-seq (nucleotide resolution) and ChIP-seq strongly differs. How does this impact the velocity estimation?

In our transcription velocity estimation, the smallest intervals are the TSS to pausing sites (with a medium of 52 bp) defined by START-seq 5' capped areas. This detail is achievable with TT-seq (but important to note that transcription rates from TT-seq cannot be interpreted at base-pair resolution). The mean insert size of the Pol II S5p MINUTE-ChIP is ~200bp, hence it is true that the precise velocity at the pausing site cannot be determined. However, we believe that the resolution of both methods is absolutely sufficient to determine gene-level velocities and to identify trends within the gene body.

Another potential concern is that RNAPII S5P is most reliably detected at the 5'-region of genes. S5P signals are significantly lower and are less reliably detected across the gene-body with the consequence that fluctuations between measurements can have stronger impact on the velocity estimation in these later elongation stages. Why have the authors not also included S2P ChIP-seq data which is more established to resemble the elongating form of RNAPII?

Pol II S5p appears after transcription initiation, before S2p is established, and indeed peaks at the pausing site. It is important to note that Pol II S5p is a modification within the

52xYSPTSPS CTD repeats, and even a single phosphorylated epitope at any of the 52 repeats would be sufficient to immunoprecipitate a Pol II molecule (the antibody is known to recognize S5p in the presence of S2p). It is difficult to assess if and how the density of S5p would affect ChIP efficiency (given the mixed contribution of affinity and avidity to binding and retention of the molecules during the immunoprecipitation and subsequent washing). However, it is worth noting that in the MINUTE-ChIP protocol short washes were used after immunoprecipitation, hence favoring retention of all Pol II S5p species irrespective of their S5p density (avidity).

All of the above lead us to hypothesize that Pol II S5p is a promising epitope for quantitatively tracking transcribing Pol II. Our validation clearly shows that the combination of TT-seq and Pol II S5p agrees well with direct gene-level measurements of Pol II velocity (Fig 3H). Importantly, the integration of both signals generated plausible velocity profiles across the gene body, with a stable velocity across the gene body and a slight increase for some genes towards the TES.

To address the reviewer question, we calculated gene level velocities with a Pol II S2p dataset, derived also from the same MINUTE-ChIP pool, and found perfect correlations with the velocities obtained using Pol II S5p (Appendix Fig S1H). We further calculated gene level velocity combining our TT-seq data with occupancy of transcriptionally engaged Pol II obtained from published NET-seq data (Mylonas & Tessarz, 2018), and this estimate also correlated well with our original method (Appendix Fig S1I).

(3) I have several comments regarding the potential link between the elongation velocity and the 'transcription termination readthrough'. The authors use TT-seq data to define transcription termination sites using a method that 'determines the maximal density contrast'. This method should be briefly described in the main text since not every reader is familiar with this approach.

We thank the reviewer for the suggestion. We have added the following brief explanation to the main text: " To define the genome-wide main termination sites from our TT-Seq data, we used a one-step global profile detection to locate the position that had the greatest difference in the density of labeled RNA before and after this site within a 15kb potential

termination window downstream of the TES, which allows a rapid scan irrespective of the local fluctuation of read sparsity (Fig 5A, Materials and Methods). "

The authors claim that 'fast elongation genes had longer read-through distances'. However, the differences in the observed read-through distances between some of the groups, for instance between the medium and fast groups (Fig. 5D) appear marginal. What is the actual median size of the termination window for the different gene groups shown in Fig. 5D? Are these differences significant?

We plotted the distributions of termination distances for the groups in Fig 5D and as boxplot in Fig 5E to allow a better comparison. We also calculated p-values against the “very slow” group. For example, there is a statistically significant increase in readthrough distance between “medium” to “very fast” groups and the “very slow” group. The median sizes of termination distance from slow to fast are 5.9, 5.8, 6.1, 6.3, and 6.5 kb in SL condition.

In this context the authors also state: 'Thus, our data supports a model in which Pol II encounters termination roadblocks imposed by DNA sequence, and the remaining velocity at the encounter stochastically determines if the polymerase comes to a complete stop.' This statement is purely speculative and should be transferred to the discussion section. Furthermore, the authors state 'In the model described above, G/C-rich motifs thus provide roadblocks to RNA polymerase II that lead to a final termination if the velocity is already sufficiently reduced.' This statement is based on correlative data but presented as a causal relationship and therefore should be softened.

We have moved the discussion of the model to the Discussion section and also changed the statement to: "Hence termination site usage is determined by multiple factors and the exact changes of termination site usage are gene-specific and may depend on GC-rich motif occurrence in the potential termination window (Fig EV5F-G). Our observations are in line with a model where RNA Pol II traverse encounters termination roadblocks imposed by DNA sequence or antisense transcription collision, and the remaining velocity at the encounter stochastically determines if the polymerase comes to a complete stop (Fig 5J-K; Fig EV5B). Our model is also compatible with the “torpedo” model in which the 5'-3' exonuclease XRN2 degrades the cleaved nascent transcript and leads to eviction of Pol II once it caught up to

Pol II (West *et al*, 2004; Brannan *et al*, 2012; Nojima *et al*, 2015; Baejen *et al*, 2017)"

(4) The authors claim 'that RNA synthesis decrease in the pluripotent state transitions is independent from elongation slowdown and neither controlled by pause release dynamics (Fig 3L, Fig EV3B).' Based on the data that is provided this conclusion appears as too premature and should be softened or removed.

We thank the reviewer for the comment. We have removed the statement.

Minor points:

(1) The manuscript contains many typos.
We apologize for the typos and have edited them accordingly.

(2) The font sizes in several Figures are small and are hard to read.

We increased the font sizes in Fig 1, Fig 2, and Fig 3.

(3) The distribution of estimated elongation velocities for all detected genes should be given.
The table of estimated elongation velocities has been attached as a supplementary file, and uploaded to the git repository: “fig4/data/Est_transcription_velocity_mm9.txt”.

(4) Fig. EV4A: how many genes are included in this analysis? The N-value should be added. What genes were included here? A clarification would be helpful.

448 genes overlapped between our recalculated velocities (1944) and the provided velocities (1197) (Jonkers *et al*, 2014).

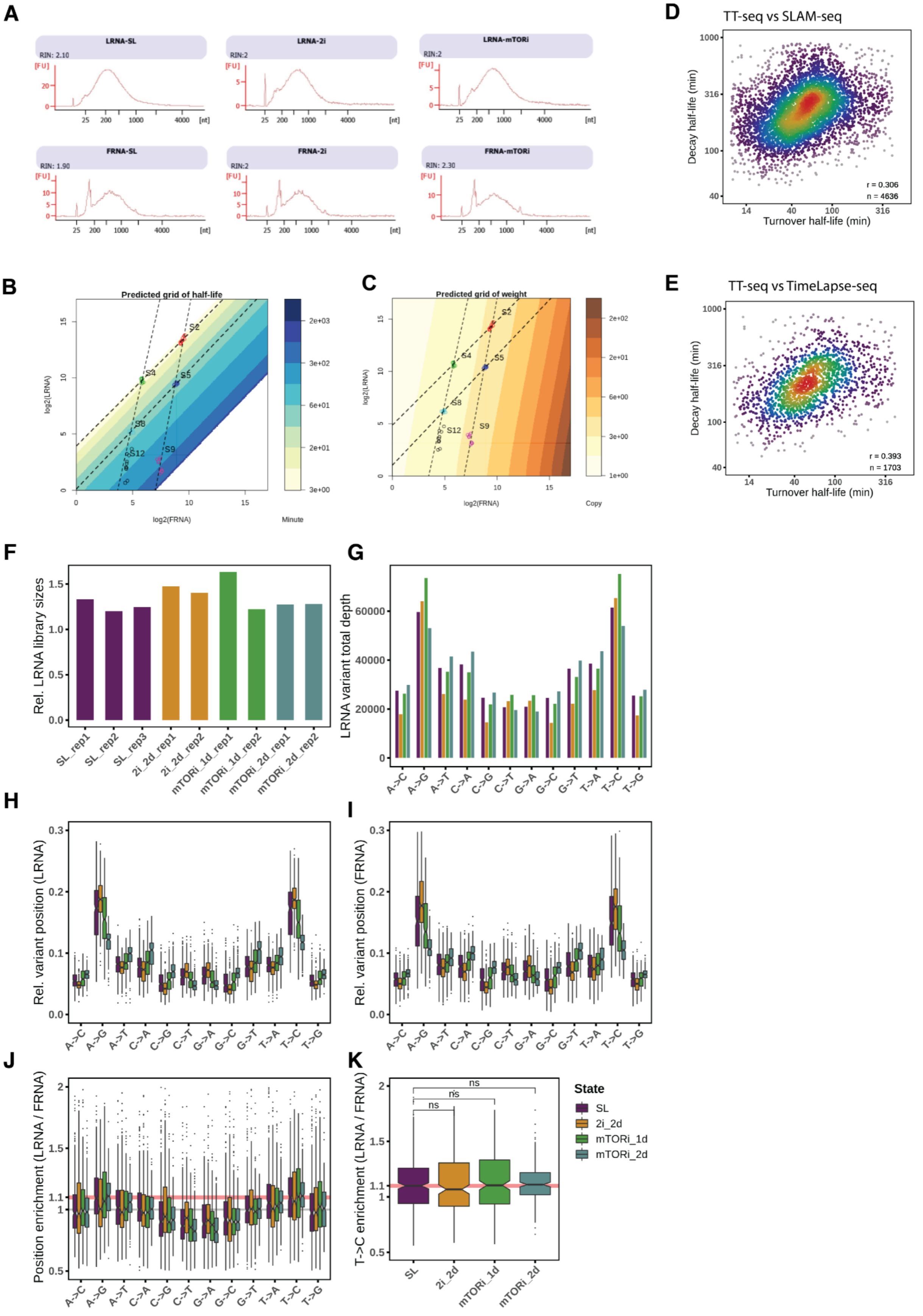
A
LRNA-SL
RIN: 2.10
LRNA-2i
RIN:2
LRNA-mTORi
RIN:2
FRNA-SL
RIN: 1.90
FRNA-2i
RIN:2
FRNA-mTORi
RIN: 2.30
[FU]
[nt]
B
Predicted grid of half-life
log2(LRNA)
log2(FRNA)
Minute
C
Predicted grid of weight
log2(LRNA)
log2(FRNA)
Copy
D
TT-seq vs SLAM-seq
Decay half-life (min)
Turnover half-life (min)
r = 0.306
n = 4636
E
TT-seq vs TimeLapse-seq
Decay half-life (min)
Turnover half-life (min)
r = 0.393
n = 1703
F
Rel. LRNA library sizes
SL_rep1
SL_rep2
SL_rep3
2i_2d_rep1
2i_2d_rep2
mTORi_1d_rep1
mTORi_1d_rep2
mTORi_2d_rep1
mTORi_2d_rep2
G
LRNA variant total depth
A->C
A->G
A->T
C->A
C->G
C->T
G->A
G->C
G->T
T->A
T->C
T->G
H
Rel. variant position (LRNA)
I
Rel. variant position (FRNA)
J
Position enrichment (LRNA / FRNA)
K
T->C enrichment (LRNA / FRNA)
ns
ns
ns
SL
2i_2d
mTORi_1d
mTORi_2d
State
SL
2i_2d
mTORi_1d
mTORi_2d

Author Response Figure 1

A. Bioanalyzer profile of labeled RNA (upper) and fragmented RNA (lower) in serum LIF, 2i 2 days, and mTORi 1 day.

B. Spike-in RNAs normalized log RPK distribution in the FRNA and LRNA space. Color scale shows the trained scale of RNA half-life.

C. Spike-in RNAs space of trained RNA copy number per cell.

D. Correlation between TT-seq estimated RNA half-lives with SLAM-seq (Herzog *et al*, 2017) measured RNA half-lives. Pearson's correlation is shown.

E. Correlation between TT-seq estimated RNA half-lives with TimeLapse-seq (Schofield *et al*, 2018) measured RNA half-lives. Pearson's correlation is shown.

F. Labeled sample sizes estimated with DESeq's size factor by reads from each chromosome.

G. Total sequencing depth of single nucleotide variant. Variant calling was performed with R package "VariantTools"; top expressed 113 genes with at least 120 single nucleotide variants were kept for downstream analysis. Positions with over 40% alternative variants were treated as non-specific variants. The summation of variant depths by each cell state were shown as above.

H. Labeled RNA variant types relative frequency on the top transcribed 200 genes by cell states.

I. Fragmented RNA (total RNA) variant types relative frequency on the top transcribed 200 genes by cell states.

J. Contrast of variant frequency between labeled and total libraries.

K. Statistical test of T->C conversion enrichment in labeled RNA over total RNA across the cell states. Unpaired two-tailed Student's t-test is performed.

Reviewer #2:

In their manuscript entitled "Newly synthesized RNA Sequencing Characterizes Transcription Dynamics in Three Pluripotent States" Shao et al. use transient transcriptome sequencing (TT-seq) - a previously described technique that allows measuring both nascent and total RNA - to distinguish gene expression features of mouse embryonic stem cells (mESCs) that were grown in three different culture conditions. These conditions are serum with leukemia inhibitory factor (LIF) (SL), serum-free in presence of LIF and inhibitors against MAPK signaling and GSK3 (2i) and serum with inhibition of mTOR signaling (mTORi). While SL and 2i represent the two most widely used approaches to propagate mESCs in an undifferentiated state, mTORi induces a general metabolic slow-down resulting in adaptation of a state reported to resemble embryonic diapause, a feature of murine development. After some useful validation experiments, including the comparison of TT-seq with published data generated with other approaches capturing nascent transcripts, the authors present data that clearly demonstrate that different culture conditions have profound effects on the abundance of different classes of transcripts via affecting both synthesis and turnover. In particular, they show that the previously reported reduction in total RNA in mTORi is due to a general, not locus-specific transcript reduction while the reduced RNA abundance in 2i appears locus-specific. In addition, they provide evidence for local co-regulation of transcript abundance and use TT-seq together with an optimized PolII ChIP-seq approach to compare elongation velocity and its impact on transcription termination in the different conditions. Overall, the experiments in this manuscript are well-designed and the data is well-analyzed. Also, due to their developmental flexibility and self-renewal capacity, mouse pluripotent cells represent an excellent model to study the impact of signaling and other factors on transcriptional regulation. However, while TT-seq as used by the authors clearly has the potential to identify novel molecular features of this important cell type, in my opinion the current manuscript should be further improved in this respect. I have listed my specific comments for consideration by the authors below.

----

1. The finding of locus-specific effects on transcript abundance in 2i is intriguing, but apart from some speculative statements the authors do not further investigate the mechanisms that underlie this observation. For example, can ectopic expression of MYC rescue the abundance of specific transcripts as the authors appear to apply?

Inhibition of MEK or GSK3 pathway is known to down-regulate Myc gene expression in many cell systems, e.g. mESC (Marks *et al*, 2012), neuroblastoma (Duffy *et al*, 2014), rhabdomyosarcoma (Marampon *et al*, 2006), and breast cancer (Duncan *et al*, 2012). The Ras signalling pathway targets the Myc gene (Bachireddy *et al*, 2005), positioning upstream of both MEK and GSK3 pathways that activate Myc expression.

We are using MYC as an example of co-regulation of neighbouring genes, but do not imply that MYC transcript itself or the encoded MYC protein mediates co-expression. Thus, we would not expect that overexpression of MYC from a transgene would activate in trans TUs neighboring the MYC locus. Which genetic elements, cis- and trans-activating factors are needed to co-regulate gene neighborhoods is a question beyond the scope of our manuscript. The gene-lncRNA co-regulation has been case studied by Engreitz et al Nature 2016, which suggests a neighboring effect of lncRNA connected to the main gene transcription and splicing. Promoter-enhancer pairing has been found to be stable in the 2i ground state transition (Atlasi et al. 2019). But in which degree the pre-established co-regulation is retained in 2i and mTORi states has been still unclear. Recently, Zuin et al. 2021 bioRxiv discovered a similar distribution of neighboring enhancer-promoter contact probability profile resembling our log2FC neighbor correlation (Fig 2E). This result fills the gap between the preservation of chromatin interaction in pluripotent state transitions and concomitant gene-ncRNA coregulation under signaling cues.

Similarly, are the observed effects downstream of MAPK inhibition, GSK3 inhibition or due to the absence of serum? A set of straightforward culture experiments could help clarifying these questions and further support the authors' claim in the Abstract that "signaling pathways ...immediately control key parameters of transcription".

We have now included new TT-seq data from SL2i cells to answer this question, and refer to it in various figures and the main text. In summary, the SL2i condition mimics 2i in many aspects, hence 2i having a dominant effect over serum as a variable (Author Response Figure 2A; Fig 1E,F; Fig EV2F; Fig EV3D; Fig EV4B).

---

2. Are there any genes that violate the slower elongation trend in 2i? If so, what are these genes?

There are 579 genes with log2FC > 1.5 speed-up in 2i state. We did a GO analysis and found that they are mainly involved in negative regulation of development. This is now presented in Fig EV4E and also addressed in the main text. We also observed gene Kit upregulation in 2i state with velocity acceleration (Author Response Figure 2C; Fig EV4D), in line with its biological function of enhancing stem cell self-renewal and proliferation.

----

3. The manuscript currently provides no validation of the success of inhibitor treatment, i.e. it remains uncertain that their cells indeed exhibit canonical features of SL, 2i and mTORi. Marker genes such as those identified by Bulut-Karslioglu et al 2016 and others might be helpful in this respect.

We thank the reviewer for the suggestion. We now provide the mESC pluripotency marker gene total RNA copy per cell in the three pluripotent states (Author Response Figure 2C). The general RNA copy number changes are coherent with the previous studies, e.g. Myc and Dnmt1l are down-regulated in 2i state (Marks *et al*, 2012), Nanog and Zfp42 are slightly down-regulated in mTORi state (Bulut-Karslioglu *et al*, 2016). The main difference of gene expression is for mTORi extreme up-regulations, which could be attributed to the long term treatment (mTORi 14 days, GSE81285, no replicate) while we measured mTORi early responses after 1 day. But in general, our differential expression results in mTORi 1 day agree with Bulut-Karslioglu et al.'s trend of changes in 14 days (Appendix Fig S1E). Moreover, we were able to reanalyze Galonska et al. and Finley et al. RNA-seq data from the comparable 2i incubation duration. Fold changes of differentially expressed genes (adjusted p-value < 0.1) correlated well with our data (Author Response Figure 2D). This result indicates that 2i induces the same program of gene differential expression in our assay and the public assays.

----

4. With respect to the experiments linking dilution by cell division to RNA turnover -Fig1G is hard to interpret without knowing what "absolute" confluency these cell densities correspond to. Representative images would be helpful. Also, without replicates, the conclusion that cell density caused the apparent differences in RNA turnover (instead of an unknown variable between the experiments) remains unconvincing. Why is rep3 SL budging the trend?

We thank the reviewer for this suggestion. Now we added representative pictures for the cell densities corresponding to the relevant datasets in Fig 1A and additional images in Author Response Figure 2E. Because mESCs grow in colonies, “confluency” per se is hard to determine.

The datasets are independent biological replicates, labeled at separate dates. TT-seq library preparation was performed in two batches:

**First batch**	**Labeling Date**	**Second batch**	**Labeling Date**
SL_rep1	3/6/2019	SL_rep3	5/15/2019
SL_rep2	3/18/2019	2i_7d	5/30/2019
2i_2d_rep1	3/18/2019	mTOR_1d_rep2	3/24/2019
2i_2d_rep2	3/14/2019	mTOR_2d_rep1	5/17/2019
mTOR_1d_rep1	3/18/2019	mTOR_2d_rep2	5/17/2019

Overall we understand the reviewers concern and have removed the panel and associated discussion from the revised manuscript.

---

5. All the experiments seemed to be conducted with a single ESC line. It would be helpful to confirm key findings (such as those shown in Fig. 2A) with at least a second line, ideally on a different genetic background.

For establishment of the method and unrelated research questions we did collect TT-seq data in two additional mESC backgrounds. A previous study (Żylicz *et al*, 2019) also performed TT-seq in a different mESC line. Amongst all of ours and published TT-seq datasets, the labeled mRNA agree very well with each other (Author Response Figure 2F). Thus, genetic background appears not to be a significant contributor to genome-wide transcriptional regulation. In addition, we have cross-validated our TT-seq total RNA data with existing RNA-seq datasets from SL, 2i and mTORi conditions, again collected in other mESC backgrounds (Appendix Fig S1C, F).

Minor comments

6. The authors describe SL as a "primed" condition, which is misleading since this

nomenclature is commonly reserved for epiblast-stem cells (EpiSCs), which are grown in absence of LIF. Both SL and 2i are naïve conditions, with 2i often described as ground state.

We thank the reviewer for the comment. We changed the nomenclature accordingly.

7. A brief description on the principles of TT-seq and how they compare to other similar techniques would be helpful for the (non-expert) reader.

We added a comparison diagram of nascent RNA-seq workflows in Fig EV1J.

8. A brief discussion on how the molecular differences observed by the authors' might be resolved during differentiation (or how they might impact upon differentiation capacity) would be interesting.

We agree this is an interesting question for the future, but we hope that the reviewers agree this would be beyond the scope of the current manuscript. We would certainly expect more extensive rewiring of RNA synthesis and decay upon differentiation. However, one interesting aspect of our study is that even closely related pluripotent states show considerable differences in transcriptional regulation.

9. Many figures have components that are too small to be easily read (examples are log2FC values in Fig. 2A).

We increased the font sizes in Fig 1, Fig 2, and Fig 3.

10. The authors write in the Discussion "Spurious transcription ....may be an effect of the global CpG hypermethylation observed in 2i". I believe they might have meant to say hypomethylation.

Correct, and we removed this sentence from the Discussion.

11. The authors' claim that their experiments in addition to studying different cell states also provide insight into transitions between these states. Considering that the earliest timepoints assessed are 2 days (for SL-to-2i) or 1 day (for SL-to-mTORi) this might represent an overstatement and should be adjusted.

We thank the reviewer for the suggestion and we edited the text accordingly.

12. Typo "steadt" instead of "steady" in first paragraph of Results section.

We edited accordingly.

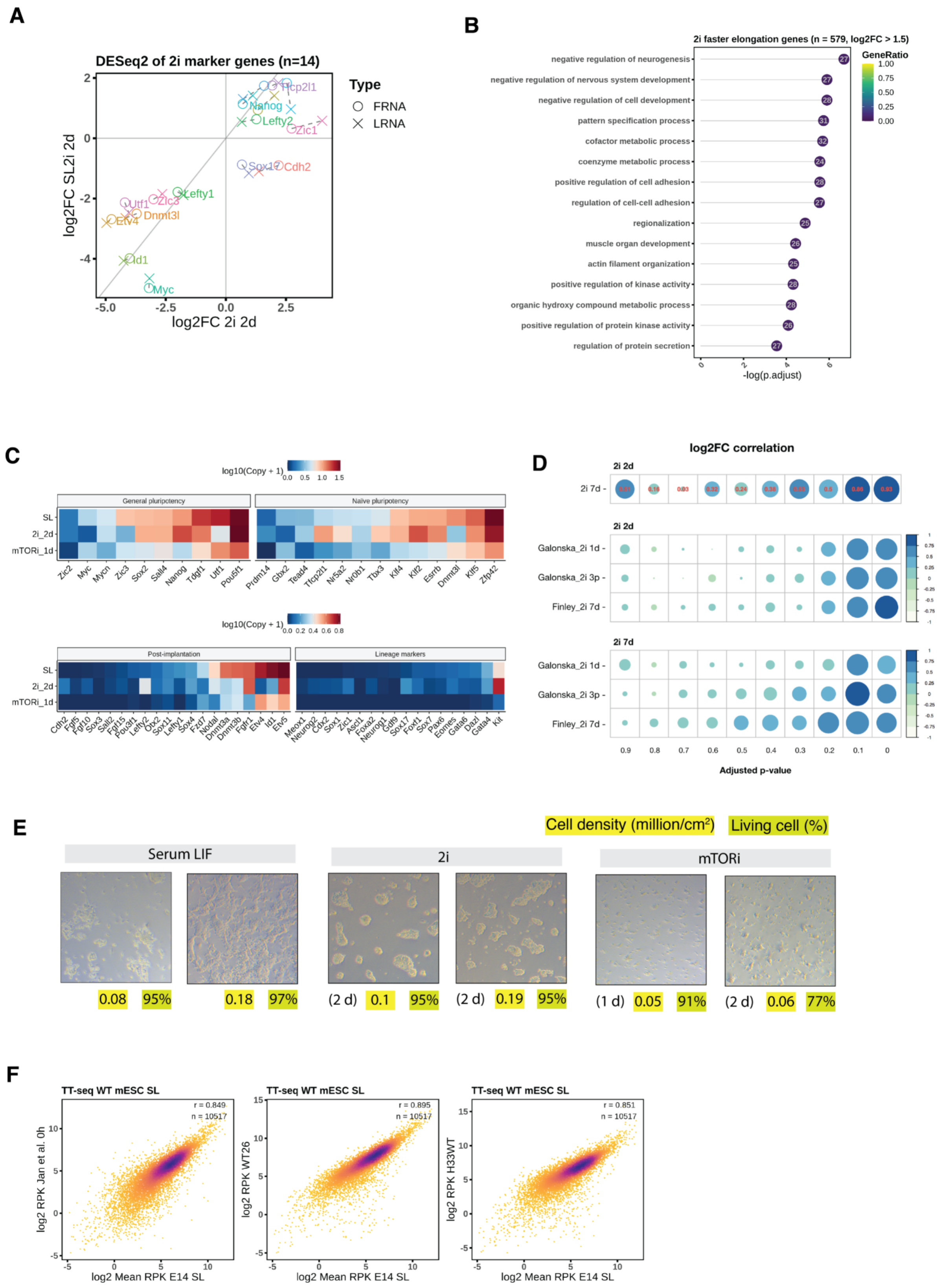
A
DESeq2 of 2i marker genes (n=14)
log2FC SL2i 2d
log2FC 2i 2d
Type
FRNA
LRNA
B
2i faster elongation genes (n = 579, log2FC > 1.5)
GeneRatio
-log(p.adjust)
negative regulation of neurogenesis
negative regulation of nervous system development
negative regulation of cell development
pattern specification process
cofactor metabolic process
coenzyme metabolic process
positive regulation of cell adhesion
regulation of cell-cell adhesion
regionalization
muscle organ development
actin filament organization
positive regulation of kinase activity
organic hydroxy compound metabolic process
positive regulation of protein kinase activity
regulation of protein secretion
C
log10(Copy + 1)
General pluripotency
Naive pluripotency
Post-implantation
Lineage markers
D
log2FC correlation
Adjusted p-value
E
Cell density (million/cm²)
Living cell (%)
Serum LIF
2i
mTORi
0.08 95%
0.18 97%
(2 d) 0.1 95%
(2 d) 0.19 95%
(1 d) 0.05 91%
(2 d) 0.06 77%
F
TT-seq WT mESC SL
log2 RPK Jan et al. 0h
log2 RPK WT26
log2 RPK H33WT
log2 Mean RPK E14 SL
r = 0.849
r = 0.895
r = 0.851
n = 10517

Author Response Figure 2

A. DESeq log2FoldChanges of labeled (LRNA) and total (FRNA) samples between 2i 2 days and serum-LIF 2i 2 days (SL2i) versus SL state. The diagonal line indicates the exact match of log2FC, and two genes in the bottom-right coordinate indicate 2i cell specific up-regulation.

B. Gene ontology biological processes of top 579 genes with increased elongation velocity in SL->2i transition.

C. ES cell general/naive/post-implantation/linage pluripotency marker genes (Kalkan *et al*, 2017; Liu *et al*, 2018) total RNA abundance in the three culture conditions.

D. Comparison of DESeq log2FC correlation between total RNA 2i 2 days changes with public RNA-seq data (Galonska *et al*, 2015; Finley *et al*, 2018). Both our data and public data were counted with Kallisto and tested with DESeq2, and Pearson's correlation was performed at each degree of average p.adjust values in the five samples.

E. Cell culture confluence in the three pluripotent states for TT-seq labeling.

F. TT-seq labeled RNA gene RPK correlation among wild-type mESCs in SL state. RW4 E14 is the strain used in this study (male, 129X1/SvJ), compares with TX1072 (female, C57BL/6) (Żylicz *et al*, 2019), WT26 (E14, male, C57Bl/6J)(Elsässer *et al*, 2015), and H33WT (E14, male, 129×C57Bl/6J)(Elsässer *et al*, 2015). Spearman's correlation coefficient is shown.

Reviewer #3:

Summary of findings

This manuscript presents a very comprehensive computational analysis that examines the relationship between signaling pathways, chromatin features and transcriptional dynamics in mouse embryonic stem cells (ESCs) including coding and non-coding transcriptional units. Experimentally, the authors measure nascent transcripts using TT-seq, steady state RNA levels and Pol II ser5p localization using MINUTE-ChIP in three different different pluripotency states of ESCs by changing culture conditions namely: naive (2i), primed (serum/LIF) and paused (mTORi). Their analysis suggests that RNA synthesis predominantly controls transcriptional output in these states and can explain the global RNA reduction between serum/LIF and 2i/mTORi conditions. They further present TT-seq with Pol II ser5p localization as an appropriate means to quantify transcription velocity which relates to termination read-through and is in turn attenuated in the naive and paused states.

This study highlights the importance of measuring transcriptional dynamics in different pluripotency states and contributes to understanding the interplay between signaling pathways, chromatin rearrangements and transcriptional control.

Major comments:

----

1. Overall the significance of studying transcriptional dynamics in the different pluripotency states should be emphasized throughout the manuscript. The authors put a lot of effort into integrating chromatin states in their analysis but this is not reflected in the abstract.

We thank the reviewer for the suggestion. We have edited the abstract, introduction and discussion accordingly.

----

2. The authors nicely demonstrate the robustness of using TT-seq to measure RNA synthesis and validate it by comparing it to other methods used in ESCs. It would make the paper more accessible to the reader if the differences between these methods are introduced or perhaps explained via a schematic depiction especially for TT-seq. It would also be helpful for the reader to explain the complementarity between these methods. e.g: the use of GRO-seq and

START-seq in Figure4 or the combination of TT-seq and NET-seq? Also, the MINUTE-chip method details are missing.

We thank the reviewer for the suggestion. We have now added a schematic diagram describing TT-seq, GRO-seq, and PRO-seq in Fig EV1J.

The complementarity of our velocity estimation method has been mentioned in the Discussion accordingly. Integration with NET-seq was also able to reproduce the Pol II-S5p measured velocity (Appendix Fig S1I). However NET-seq sample normalization with separate pull-down batches and the present of spliced reads would be concerns (Fig EV1B), as we discussed our method's advantage: "due to the simplicity of sample normalization compared to the other velocity estimation methods (inhibitor+GRO-seq or NET-seq)"

We also now explain the MINUTE-ChIP assay in more detail in the Methods section and also briefly in the main text, highlighting that it allows for quantitative comparisons between samples.

---

3. More information on the biology of the three ESCs pluripotency states would also help frame the significance of the research, in particular why is it important to study these states and how transcriptional dynamics changes reflect stemness? Also, the rationale behind the timing for the inhibitors in relation to cell cycle: 2 days, 7days for the 2i condition versus 1 & 2days for the mTORi condition needs more explanation. A PCA analysis of the unlabeled RNA (total RNA-seq) side by side with the originally characterized transcriptomes of naive and paused states would also be helpful to validate the culture conditions/timing.

Mouse embryonic stem cells are widely used in genomic studies of embryo development, epigenetic regulation, genome organization, and transcription. The three culture conditions here all define ES cells pre-implantation pluripotency; SL is the conventional condition, mTORi mimic diapause stem cells, and 2i represents ground state pluripotency. While the transcriptomic differences are well-described in the RNA-seq level, and many studies have focused on identifying epigenomic signatures, transcription regulation itself has not been systematically studied, hence the motivation for our study. We have updated the introduction to highlight more the significance of studying transcriptional kinetics changes in the three ESCs pluripotency states.

Previous studies varied 2i treatment from 1 day (Galonska *et al*, 2015), 7 days (Marks *et al*, 2012) and up to 24 days (Smallwood *et al*, 2014). We chose a 2d 2i treatment as the earliest stable manifestation of the ground state (we essentially observed the same gene expression changes after 2d and 7d, in agreement with the reported rapid rewiring of transcription (Galonska *et al*, 2015)). Our DESeq gene differential expression results are consistent with 1d to 7d data by Galonska and Finley (Author Response Figure 2D).

The paused state by INK128 mTOR inhibition was originally evaluated at 14 days (Bulut-Karslioglu *et al*, 2016) (GSE81285). However, we observed immediate cell morphological changes and proliferation stagnation, and 4sU labeled RNA was already extremely low after 2d, hence we chose 1d treatment that already captured key gene expression changes observed at 14 days with RNA-Seq (Bulut-Karslioglu, 2016) (Appendix Fig S1E).

We added the labeled and total RNA PCA plots for the three pluripotent states comparison (Fig 1E). We also performed meta-analysis with previously published mESCs naïve state RNA-seq. Using a core list of pluripotency markers to alleviate lab / method specific variation in total transcriptomes, we are able to confirm the reproducibility of the paused state and ground states (Appendix Fig S1F).

---

4. It would be important to discuss whether the observed RNA synthesis dynamics in relation to chromatin regulation and RNA stability are features specific to the pluripotency state?

First, we have evaluated the linear explanation (Pearson's correlation and R-squared) of individual chromatin features in SL condition, and the results agree with the known association (Author Response Figure 3A). But ncRNA transcription and abundance are nearly independent of chromatin features.

Second, from these correlative observations we were careful not to imply causality, since it is clearly an unresolved issue of which epigenomic features are the causes or consequences of transcription regulation (Howe *et al*, 2017; Henikoff & Shilatifard, 2011; Morgan & Shilatifard, 2020).

Third, we do believe that the transcriptional states we observe are largely under direct influence of signaling, and thus are the key characteristics of each state.

Regarding the question about RNA stability as a state-specific feature, we stress that our turnover rates are estimates from labeled and total RNA but do agree well with half-lives measured by completely different methods in mESCs (Author Response Figure 1D-E). Our data suggest that cell division is an important source of cell-level RNA turnover, together with RNA synthesis. However, regarding RNA stability, decay is only a partial determinant of steady-state transcript abundance (the other two determinants are RNA synthesis and cell division). We believe RNA stability in the pluripotent states are differently tuned, but our measurement of RNA turnover is unable to be decomposed to RNA decay estimates. We also believe cell level RNA turnover has more biological relevance than molecular level RNA decay. So, RNA synthesis and turnover decreases are features specific to the inhibitor-induced states.

Along these lines: are there any trends between cell identity genes and transcription velocity / read-through(Figure 3&5)?

We thank the reviewer for the suggestion. Pol II velocity of most pluripotency-associated genes (see Author Response Figure 2C) was not evaluated before (Jonkers *et al,* 2014) and we were also not able to extract direct measurements for these genes in our reanalysis of their data. However, the TT-seq/MINUTE-ChIP based method allowed to estimate velocity for those genes.

So we mapped our estimated transcription velocity and termination distance to pluripotency-associated genes. In Author Response Figure 3B we show gene specific responses for some example pluripotency genes comparing the three states. For instance Tbx3 expression (labeled and total RNAs) did not change upon 2i and mTORi transitions (Fig 1B), however its estimated velocity and termination distance was decreased. More evidence of gene-specific response also appeared with Myc and Pou5f1, whose termination distances were unchanged although transcription velocity decreased. In Author Response Figure 3C we grouped genes according to ES cell general, naive, post-implantation, linage pluripotency marker genes (Kalkan *et al*, 2017; Liu *et al*, 2018) and compared transcription velocity and termination distance in a combine of all three conditions. General pluripotency genes show

fast velocity and short termination distance.The *Kit* gene is specifically upregulated in 2i state, and we also observed its velocity acceleration (Author Response Figure 2C; Fig EV4D), in line with its biological function of enhancing stem cell self-renewal and proliferation. We did a GO analysis and found that 2i accelerated genes were mainly involved in negative regulation of development. This is now presented in Fig EV4E and also addressed in the main text.

Also, since the argument is that RNA synthesis is the driver of total RNA levels in ESCs, is there a relationship between velocity and steady-state RNA levels on a gene-by-gene basis or when considering groups of fast, medium and slow genes(Figure 3&4)?

Transcription velocity has a minimal association with RNA synthesis rate as previously reported (see Figure 4F in Jonkers *et al*, 2014). We assessed RNA copy number and transcription velocity, and they are independent (Author Response Figure 3D). We agree it is an interesting question how Pol II velocity is regulated in the first place and what purpose such regulation could have if it does not affect the RNA synthesis rate, but this is beyond what we can address in the present manuscript.

---

5. The authors state that "a stranded TT-seq signal provides an excellent demarcation of the nascent transcription unit and polymerase-independent measure of transcriptional activity" Since authors measure how elongation velocity affects transcriptional termination sites and readthrough, it would be interesting to implement PolII occupancy in their coding TU discovery pipeline.

We thank the reviewer for the suggestion. The current version of “TU filter” R shiny app mainly aims for accelerating TU annotation of nascent RNA signals, which needs strand specific reads, hence it does not work with non-stranded data. It is important to note that RNA Pol II ChIP generally is not very useful for annotating TU boundaries for a number of reasons:

1) The resolution of ChIP is limited by the fragment size (50-150bp using enzymatic digestion, >250bp for sonication)
2) Total RNA Pol II ChIP has lower sensitivity. The density of Pol II in transcribed gene bodies is not very high and thus the ChIP signal is too weak to reliably detect medium to low transcribed TUs due to the signal to noise issue.

3) RNA Pol II ChIP detects, especially if performed with formaldehyde crosslinking, a substantial background from non-productive Pol II molecules interacting with DNA outside of genes. Hence RNA Pol II ChIP is unsuitable in demarcate transcribed regions.

For all the reasons above, H3K36me3 is the only ChIP-Seq feature that has been used to discover novel TUs, but also this feature is not very sensitive.

- Similarly, what is the occupancy of either PolI, PolII and PolIII over bidirectional intergenic TUs that show upregulation in the naive condition?

RNA polymerases show the same degrees of occupancy on all intergenic TU classes, including non-enhancer bidirectional TUs, which were up-regulated in 2i state: bidirectional > unidirectional, enhancer > non-enhancer. (Author Response Figure 3E-F).

- And how does their estimated velocity correlate with neighboring coding TUs in primed versus naive conditions?

We didn’t find correlation between main gene and neighboring intergenic TU velocities (Author Response Figure 3G), and the non-enhancer bidirectional TUs, which were up-regulated in 2i state (Author Response Figure 3H).

---

6. The change in local chromatin features between pluripotency states is intriguing. It would be good to discuss the possibility to use TT-seq and elongation velocity measurements in the context of inhibiting chromatin modifiers or remodelers.

We agree that having a robust assay in hand it would be very interesting to test means to perturb Pol II velocity to understand which factors regulate velocity in the first place (chromatin remodelers being an excellent suggestion), and what the consequences of slower or faster velocity would be. We added a brief discussion as follows:
“Beyond the observed association between transcription velocity and chromatin features, our quantitative strategy of transcription kinetics estimation could facilitate the future study via single molecule perturbation for dissecting the enigma of local transcription velocity control.”

Minor comments

1. How do the authors handle dead cells during the naive and paused pluripotency state conversion? This could affect RNA measurements.

We washed mESCs with PBS once before trypsinization and cell number counting, and 4sU labelling also has a medium switch step to remove detached dead cells. Cell viability was indicated by cell counter (TC20™) at an average of 90% for naive and ground state cells, and 70% for paused state cells in long treatment (2 days, possibly due to mTORi smaller cell size (Bulut-Karslioglu *et al*, 2016)) (Author Response Figure 2E). It is expected that dead cells do not uphold a significant fraction of cell population, hence not influencing labeled RNA and spike-in normalization to an extent as large as the cell level transcription changes (Bulut-Karslioglu *et al*, 2016). So we believe that dead cells are not a confounding factor in our nascent transcriptome analyses.

2. *The relevance and correlation of the transcriptional elongation reference with cdk9 inhibition GRO-seq time course should be better explained/introduced.*

We thank the reviewer for the suggestion and edited the main text with "Absolute elongation velocities have previously been determined in mESC by inhibiting pause-release using a Cdk9 inhibition (flavopiridol) followed by a GRO-seq time course (Jonkers *et al*, 2014). In this study, performed under SL condition, velocities for 1197 transcripts were reported; and we were able to extract velocities for a total of 1944 transcripts by reanalyzing the dataset using our annotation and model fitting with at least two available time points (Materials and Methods)."

3. *The model in Figure EV6 is not referenced/explained in the main text.*

We intended it as a "synopsis image".

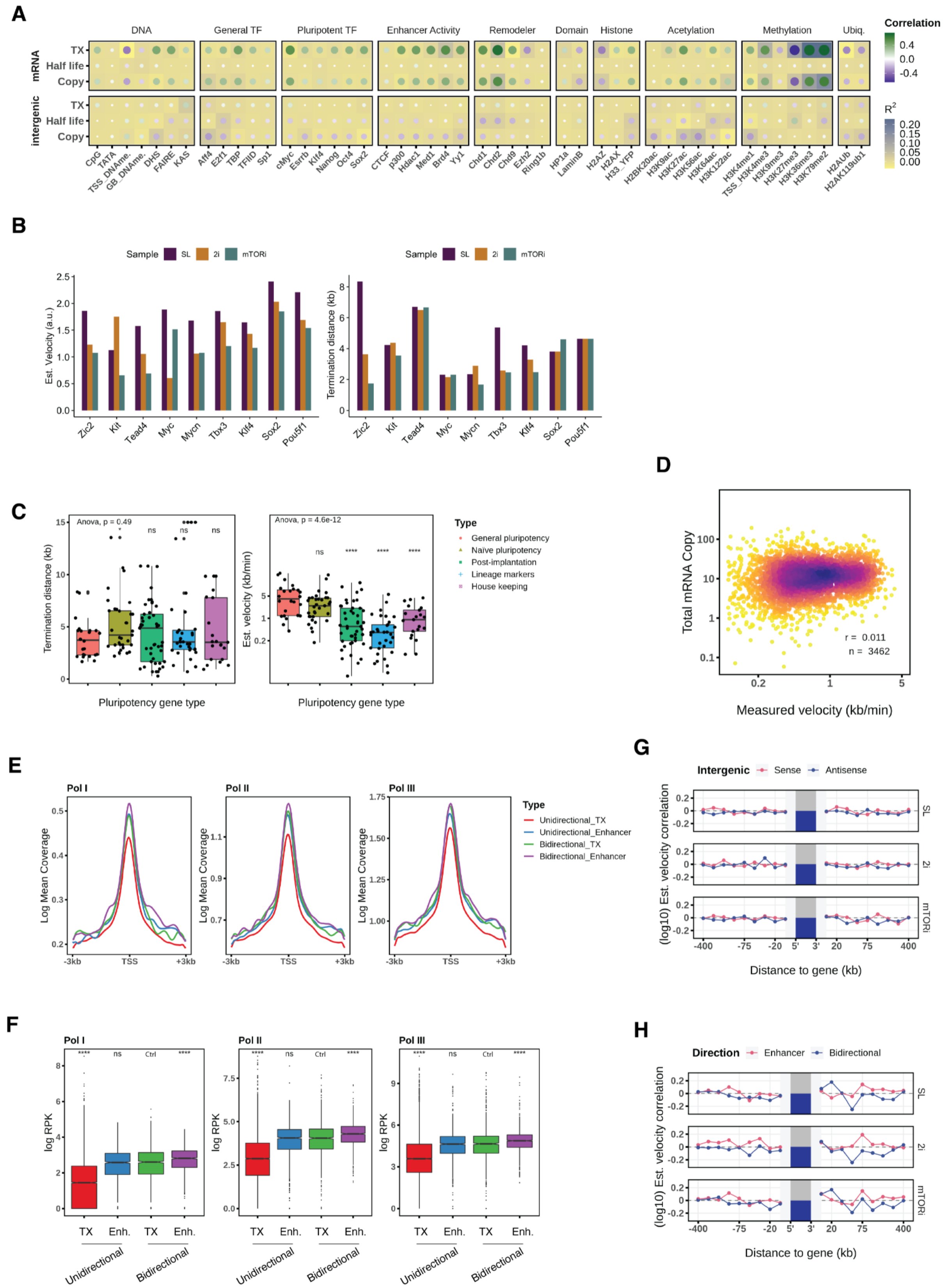

Author Response Figure 3

A. Spike-ins trained scales estimate total RNA abundance (copy/cell), RNA synthesis rate (TX, copy/min), and half-life (min). Linear explanation was performed with Pearson's correlation (dot) and R-squared (color box) between each epigenomic feature and RNA estimates in log scale.

B. Estimated transcription velocity and termination distances with examples of pluripotency genes.

C. Transcription termination distance (left), elongation velocity (right) are shown for pluripotency-associated genes. 45 genes are evaluated and grouped as described in Author Response Figure 2C, in the combination of the three states measurements for statistical tests (unpaired Student's t-test, *p=0.05, ****p=0.0001).

D. Re-calculated elongation velocity from Jonkers et al. data, does not correlate with total RNA abundance. Pearson's correlation is shown.

E. Intergenic TUs (n=14978) RNA Pol I/II/III coverage on +/- 2kb TSS intervals.

F. RNA Pol I/II/III occupancy on intergenic TUs by TU types as in E.

G. Estimated velocity of gene and intergenic TU correlation by their neighbor distance (40624 pairs).

H. Estimated velocity correlation between gene and two types of intergenic TU, enhancer (n=6183) and bi-directional non-enhancer (n=4412).

Thank you for sending us your revised manuscript. We have now heard back from the three reviewers who were asked to evaluate your revised study. As you will see below, the reviewers are satisfied with the modifications made and are supportive of publication. They do however raise a few relatively minor concerns, which we would ask you to address in a minor revision. We would also ask you to address the editorial issues listed below.

REFEREE REPORTS

Reviewer #1:

The manuscript is clearly improved and the authors have thoroughly addressed my comments. Notably, the authors added correlations between TT-seq and MINUTE-ChIP replicates to confirm the reproducibility of the data (Appendix Fig. S1A-B). Convincing evidence is provided that the labeling efficiency of the newly synthesized RNA is similar between the three pluripotent states investigated in this study. Furthermore, an extensive supplemental table with estimated elongation velocities for more than 34,000 transcription units is now included that will be a helpful resource for the transcription community. Finally, the clarifications and additional explanations that were added to the main text also improved the manuscript.

Comment:
New Fig. S1A: correlation between 2I 7Days replicates is not shown. Was only one TT-seq measurement performed for 2I 7Days? A clarification is needed.

Reviewer #2:

Shao and colleagues have addressed all major points that I raised during review of their original submission and their revised manuscript overall appears significantly improved. I have no more general concerns regarding publication of their work, but would recommend considering including the comparison of marker gene expression in the different culture conditions (Author Response Figures 2C and 2D) as well as the comparison with previous TT-seq datasets (Author Response Figure 2F) into the supplement.

Reviewer #3:

In the revised manuscript Shao and colleagues provide additional analyses of the initial TT-seq and Pol II S5p ChIP-seq profiling data for all three pluripotency states. They also include more detailed methods and new experiments in an additional naïve state in the presence of serum to demarcate the effect of the 2i small molecules.

The authors addressed all my comments. Overall the revised manuscript provides convincing data and analyses that validate their approach in measuring the transcriptional kinetics during subtle changes of pluripotency states induced by signaling molecules.

I have minor suggestion to improve the presentation of the manuscript and help the reader understand the flow of the paper:

- RNA transcription in the title should read: "DNA transcription kinetics" or "transcriptional kinetics".
- Figures are not numbered, which made it difficult to find the order while reading the main text and many figure panels are cited out of order throughout the manuscript. It would be good to rearrange panels to fit the flow of the narration.
- Provide labeling of conditions in Fig 1E for each dot or add a legend.
- Net-seq method is not described in the schematic of Fig EV1J
- Provide a table of state specific genes used in Appendix Fig S1F
- Figures 3 through 5 can benefit from showing examples of pausing, velocity and termination distance measurements together with profiles of Pol II S5p binding for representative genes shown in Author Response Figure 3C in each pluripotency state, as presented for RNA-seq in Author Response Figure 2C.

Reviewer #1:

The manuscript is clearly improved and the authors have thoroughly addressed my comments. Notably, the authors added correlations between TT-seq and MINUTE-ChIP replicates to confirm the reproducibility of the data (Appendix Fig. S1A-B). Convincing evidence is provided that the labeling efficiency of the newly synthesized RNA is similar between the three pluripotent states investigated in this study. Furthermore, an extensive supplemental table with estimated elongation velocities for more than 34,000 transcription units is now included that will be a helpful resource for the transcription community. Finally, the clarifications and additional explanations that were added to the main text also improved the manuscript.

Comment:
New Fig. S1A: correlation between 2i 7Days replicates is not shown. Was only one TT-seq measurement performed for 2i 7Days? A clarification is needed.

There is only one dataset for 2i_7d included in the study. We initially only sequenced one replicate of 7-day 2i treatment to determine if there would be further transcriptome changes between 2 and 7 days 2i culture. Transcriptomes of cells in 2i for 2 days and 7 days were very similar (Fig 1E and Appendix Fig 1A, now Fig EV1A), hence we performed all subsequent analysis with the 2i_2d replicates and did not further investigate the 7d timepoint. And the text has been edited accordingly: “); “TT-seq data of cells cultured in 2i medium for 2 or 7 days correlated well (EV1A) and we therefore focused on the primary responses after 2 days in all further analyses.”

Reviewer #2:

Shao and colleagues have addressed all major points that I raised during review of their original submission and their revised manuscript overall appears significantly improved. I have no more general concerns regarding publication of their work, but would recommend considering including the comparison of marker gene expression in the different culture conditions (Author Response Figures 2C and 2D) as well as the comparison with previous TT-seq datasets (Author Response Figure 2F) into the supplement.

Done (Fig EV1)

Reviewer #3:

In the revised manuscript Shao and colleagues provide additional analyses of the initial TT-seq and Pol II S5p ChIP-seq profiling data for all three pluripotency states. They also include more detailed methods and new experiments in an additional naïve state in the presence of serum to demarcate the effect of the 2i small molecules.

The authors addressed all my comments. Overall the revised manuscript provides convincing data and analyses that validate their approach in measuring the transcriptional kinetics during subtle changes of pluripotency states induced by signaling molecules.

I have minor suggestion to improve the presentation of the manuscript and help the reader understand the flow of the paper:

- RNA transcription in the title should read: "DNA transcription kinetics" or "transcriptional kinetics".
We thank the reviewer for the advice and edited the title accordingly.

- Figures are not numbered, which made it difficult to find the order while reading the main text and many figure panels are cited out of order throughout the manuscript. It would be good to rearrange panels to fit the flow of the narration.
We now reordered the figures and arranged the panels in the order of occurrence in the text as much as possible.

- Provide labeling of conditions in Fig 1E for each dot or add a legend.
We now add point labels in Fig 1E.

- Net-seq method is not described in the schematic of Fig EV1J
We now add an illustration of NET-seq as Fig EV2A.

- Provide a table of state specific genes used in Appendix Fig S1F
We now provide the state specific genes as a supplementary Table EV2.

- Figures 3 through 5 can benefit from showing examples of pausing, velocity and termination distance measurements together with profiles of Pol II S5p binding for representative genes shown in Author Response Figure 3C in each pluripotency state, as presented for RNA-seq in Author Response Figure 2C.
We thank the reviewer for the suggestion, now we added an extra panel in Fig EV7.

Accepted **14th Dec 2021**

Thank you again for sending us your revised manuscript. We are now satisfied with the modifications made and I am pleased to inform you that your paper has been accepted for publication.

EMBO PRESS

YOU MUST COMPLETE ALL CELLS WITH A PINK BACKGROUND ↓

PLEASE NOTE THAT THIS CHECKLIST WILL BE PUBLISHED ALONGSIDE YOUR PAPER

Corresponding Author Name: Simon Elsässer

Journal Submitted to: Molecular Systems Biology
Manuscript Number: MSB-2021-10407

Reporting Checklist For Life Sciences Articles (Rev. June 2017)

This checklist is used to ensure good reporting standards and to improve the reproducibility of published results. These guidelines are consistent with the Principles and Guidelines for Reporting Preclinical Research issued by the NIH in 2014. Please follow the journal's authorship guidelines in preparing your manuscript.

A- Figures

1. Data

The data shown in figures should satisfy the following conditions:

- the data were obtained and processed according to the field's best practice and are presented to reflect the results of the experiments in an accurate and unbiased manner.
- figure panels include only data points, measurements or observations that can be compared to each other in a scientifically meaningful way.
- graphs include clearly labeled error bars for independent experiments and sample sizes. Unless justified, error bars should not be shown for technical replicates.
- if n< 5, the individual data points from each experiment should be plotted and any statistical test employed should be justified
- Source Data should be included to report the data underlying graphs. Please follow the guidelines set out in the author ship guidelines on Data Presentation.

2. Captions

Each figure caption should contain the following information, for each panel where they are relevant:

- a specification of the experimental system investigated (eg cell line, species name).
- the assay(s) and method(s) used to carry out the reported observations and measurements
- an explicit mention of the biological and chemical entity(ies) that are being measured.
- an explicit mention of the biological and chemical entity(ies) that are altered/varied/perturbed in a controlled manner.

- the exact sample size (n) for each experimental group/condition, given as a number, not a range;
- a description of the sample collection allowing the reader to understand whether the samples represent technical or biological replicates (including how many animals, litters, cultures, etc.).
- a statement of how many times the experiment shown was independently replicated in the laboratory.
- definitions of statistical methods and measures:
  - common tests, such as t-test (please specify whether paired vs. unpaired), simple $\chi 2$ tests, Wilcoxon and Mann-Whitney tests, can be unambiguously identified by name only, but more complex techniques should be described in the methods section;
  - are tests one-sided or two-sided?
  - are there adjustments for multiple comparisons?
  - exact statistical test results, e.g., P values = x but not P values < x;
  - definition of 'center values' as median or average;
  - definition of error bars as s.d. or s.e.m.

Any descriptions too long for the figure legend should be included in the methods section and/or with the source data.

In the pink boxes below, please ensure that the answers to the following questions are reported in the manuscript itself. Every question should be answered. If the question is not relevant to your research, please write NA (non applicable). We encourage you to include a specific subsection in the methods section for statistics, reagents, animal models and human subjects.

USEFUL LINKS FOR COMPLETING THIS FORM

http://www.antibodypedia.com
http://1degreebio.org
http://www.equator-network.org/reporting-guidelines/improving-bioscience-research-repor
http://grants.nih.gov/grants/olaw/olaw.htm
http://www.mrc.ac.uk/Ourresearch/Ethicsresearchguidance/Useofanimals/index.htm
http://ClinicalTrials.gov
http://www.consort-statement.org
http://www.consort-statement.org/checklists/view/32-consort/66-title
http://www.equator-network.org/reporting-guidelines/reporting-recommendations-for-tumo
http://datadryad.org
http://figshare.com
http://www.ncbi.nlm.nih.gov/gap
http://www.ebi.ac.uk/ega
http://biomodels.net/
http://biomodels.net/miriam/
http://jjj.biochem.sun.ac.za
https://osp.od.nih.gov/biosafety-biosecurity-and-emerging-biotechnology/
http://www.selectagents.gov/

B- Statistics and general methods

Please fill out these boxes ↓ (Do not worry if you cannot see all your text once you press return)

1.a. How was the sample size chosen to ensure adequate power to detect a pre-specified effect size?	Yes.
1.b. For animal studies, include a statement about sample size estimate even if no statistical methods were used.	NA.
2. Describe inclusion/exclusion criteria if samples or animals were excluded from the analysis. Were the criteria pre-established?	Yes.
3. Were any steps taken to minimize the effects of subjective bias when allocating animals/samples to treatment (e.g. randomization procedure)? If yes, please describe.	NA.
For animal studies, include a statement about randomization even if no randomization was used.	NA.
4.a. Were any steps taken to minimize the effects of subjective bias during group allocation or/and when assessing results (e.g. blinding of the investigator)? If yes please describe.	NA.
4.b. For animal studies, include a statement about blinding even if no blinding was done	NA.
5. For every figure, are statistical tests justified as appropriate?	Yes.
Do the data meet the assumptions of the tests (e.g., normal distribution)? Describe any methods used to assess it.	Yes, in general. Genomic measurements (e.g. RPK, density, coverage) follows log-normal distribution. Histogram, ridge plot, violin plot, and boxplot are applied to assess log-nomral distribution by visualization.
Is there an estimate of variation within each group of data?	Yes.

Is the variance similar between the groups that are being statistically compared?	Yes.

C- Reagents

6. To show that antibodies were profiled for use in the system under study (assay and species), provide a citation, catalog number and/or clone number, supplementary information or reference to an antibody validation profile. e.g., Antibodypedia (see link list at top right), 1DegreeBio (see link list at top right).	Yes.
7. Identify the source of cell lines and report if they were recently authenticated (e.g., by STR profiling) and tested for mycoplasma contamination.	Yes.

* for all hyperlinks, please see the table at the top right of the document

D- Animal Models

8. Report species, strain, gender, age of animals and genetic modification status where applicable. Please detail housing and husbandry conditions and the source of animals.	NA.
9. For experiments involving live vertebrates, include a statement of compliance with ethical regulations and identify the committee(s) approving the experiments.	NA.
10. We recommend consulting the ARRIVE guidelines (see link list at top right) (PLoS Biol. 8(6), e1000412, 2010) to ensure that other relevant aspects of animal studies are adequately reported. See author guidelines, under 'Reporting Guidelines'. See also: NIH (see link list at top right) and MRC (see link list at top right) recommendations. Please confirm compliance.	NA..

E- Human Subjects

11. Identify the committee(s) approving the study protocol.	NA.
12. Include a statement confirming that informed consent was obtained from all subjects and that the experiments conformed to the principles set out in the WMA Declaration of Helsinki and the Department of Health and Human Services Belmont Report.	NA.
13. For publication of patient photos, include a statement confirming that consent to publish was obtained.	NA.
14. Report any restrictions on the availability (and/or on the use) of human data or samples.	NA.
15. Report the clinical trial registration number (at ClinicalTrials.gov or equivalent), where applicable.	NA.
16. For phase II and III randomized controlled trials, please refer to the CONSORT flow diagram (see link list at top right) and submit the CONSORT checklist (see link list at top right) with your submission. See author guidelines, under 'Reporting Guidelines'. Please confirm you have submitted this list.	NA.
17. For tumor marker prognostic studies, we recommend that you follow the REMARK reporting guidelines (see link list at top right). See author guidelines, under 'Reporting Guidelines'. Please confirm you have followed these guidelines.	NA.

F- Data Accessibility

18: Provide a "Data Availability" section at the end of the Materials & Methods, listing the accession codes for data generated in this study and deposited in a public database (e.g. RNA-Seq data: Gene Expression Omnibus GSE39462, Proteomics data: PRIDE PXD000208 etc.) Please refer to our author guidelines for 'Data Deposition'.<br><br>Data deposition in a public repository is mandatory for:<br>a. Protein, DNA and RNA sequences<br>b. Macromolecular structures<br>c. Crystallographic data for small molecules<br>d. Functional genomics data<br>e. Proteomics and molecular interactions	Yes.
19. Deposition is strongly recommended for any datasets that are central and integral to the study; please consider the journal's data policy. If no structured public repository exists for a given data type, we encourage the provision of datasets in the manuscript as a Supplementary Document (see author guidelines under 'Expanded View' or in unstructured repositories such as Dryad (see link list at top right) or Figshare (see link list at top right).	Yes.
20. Access to human clinical and genomic datasets should be provided with as few restrictions as possible while respecting ethical obligations to the patients and relevant medical and legal issues. If practically possible and compatible with the individual consent agreement used in the study, such data should be deposited in one of the major public access-controlled repositories such as dbGAP (see link list at top right) or EGA (see link list at top right).	NA.
21. Computational models that are central and integral to a study should be shared without restrictions and provided in a machine-readable form. The relevant accession numbers or links should be provided. When possible, standardized format (SBML, CellML) should be used instead of scripts (e.g. MATLAB). Authors are strongly encouraged to follow the MIRIAM guidelines (see link list at top right) and deposit their model in a public database such as Biomodels (see link list at top right) or JWS Online (see link list at top right). If computer source code is provided with the paper, it should be deposited in a public repository or included in supplementary information.	Yes.

G- Dual use research of concern

22. Could your study fall under dual use research restrictions? Please check biosecurity documents (see link list at top right) and list of select agents and toxins (APHIS/CDC) (see link list at top right). According to our biosecurity guidelines, provide a statement only if it could.	Yes, our study follows the reasearch biosecurity restrictions.
